# Expansion of the Chemical Space in UiO-Type Metal–Organic Frameworks via the Ugi Four-Component Reaction for Tuned Pore Accessibility

**DOI:** 10.1021/acs.inorgchem.6c02585

**Published:** 2026-07-24

**Authors:** Erik Uribe Vega, Ma Carmen Garcia-González, Axel Uribe Vega, Salvador López Morales, Joelis Rodríguez Hernández, Erick Hernández-Santiago, Eréndira García-Ríos, Luis D. Miranda, Braulio Rodríguez-Molina

**Affiliations:** † Instituto de Química, Universidad Nacional Autónoma de México, Circuito Exterior, Ciudad Universitaria, 04510 Ciudad de México, México; ‡ Instituto de Investigaciones en Materiales, Universidad Nacional Autónoma de México, Circuito Exterior, Ciudad Universitaria, 04510 Ciudad de México, México; § Centro de Investigación en Química Aplicada, Blvd. Ing. Enrique Reyna Hermosillo No.140, Col. San José de los Cerritos, 25294 Saltillo, Coahuila, México

## Abstract

Pore functionalization of metal–organic frameworks (MOFs) remains a superb challenge for controlling intermolecular interactions and the resulting diffusion of guests. Herein, we report a modular presynthetic strategy to expand the chemical space of UiO-67 by incorporating structurally diverse dicarboxylate linkers synthesized via the Ugi four-component reaction (Ugi-4CR). This approach introduces amide-rich functionalities while preserving the *fcu* topology of the Zr-based framework, as confirmed by PXRD and solid-state ^13^C NMR spectroscopy. The Ugi-derived functionalization systematically modulates porosity and pore environment, which was further tuned through a multivariate (MTV) mixed-linker strategy. Nitrogen adsorption analysis reveals systematic modulation of surface area and pore accessibility with increasing functionalization. The prepared MOFs were used to adsorb catechol, a prototypical phenolic pollutant, which was quantified by HPLC. The MTV material exhibited the highest uptake, highlighting the interplay between pore accessibility and host–guest interactions, where excessive functionalization hinders adsorption. Overall, this work establishes multicomponent reactions as a versatile platform for tuning pore chemistry in MOFs while emphasizing the need to balance functionality to optimize performance.

## Graphical abstract

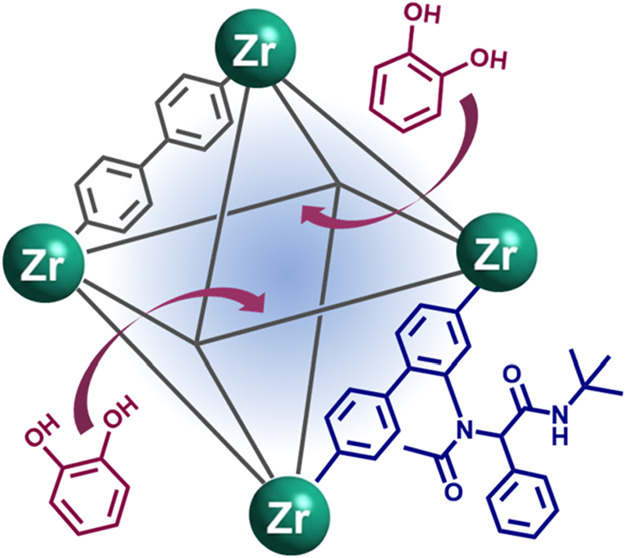

## 1 Introduction

Metal–organic frameworks (MOFs) are crystalline porous materials that have been extensively explored for applications in adsorption, separation, and molecular sensing.^1–3^ Their high surface areas and structural versatility arise from the rational design of organic linkers with tailored chemical functionalities.^4–7^ In this context, isoreticular synthesis enables the generation of functional MOF architectures while preserving the underlying framework topology, allowing systematic modulation of pore environments and material properties.^8–11^ Among the various MOFs reported to date, the UiO series represents one of the most versatile classes owing to its high thermal and chemical stability and its pronounced tolerance toward linker modification.^12–14^ In particular, UiO-67, based on a biphenyl-4,4′-dicarboxylate (BPDC) linker, provides a robust platform for structural diversification, while amino-functionalized derivatives offer a convenient synthetic handle for presynthetic modification.^15–17^

Functionalization of UiO-67 linkers has been shown to significantly influence framework properties and expand their application scope.^18–21^ For instance, sulfonated derivatives exhibit modified ion transport and electrochemical behavior, while chiral amine–alcohol functionalities enable enantioselective adsorption.^22^ Similarly, the incorporation of imidazolium-based motifs enhances CO_2_ affinity and catalytic performance through cooperative interactions with the Zr_6_ nodes.^23^ These examples highlight the critical role of linker design in tuning host–guest interactions within UiO-type frameworks.

In this context, designing MOFs with tailored pore environments is particularly attractive because it aims to improve the adsorption of organic molecules by establishing specific host–guest interactions.^24^ Catechol and related phenolic compounds are common pollutants in industrial effluents (e.g., petrochemical, pharmaceutical, and dye industries), where their toxicity and persistence raise environmental concerns.^25,26^ Consequently, understanding their adsorption behavior is also relevant for the development of advanced materials for water purification and environmental remediation.^27^ Furthermore, catechol is a representative model compound due to its ability to engage in hydrogen bonds or π-π interactions, and is highly relevant in environmental and industrial processes.^28,29^ Importantly, its ortho-dihydroxy functionality could enable hydrogen bond interactions with appropriate groups within the MOFs, making it suitable for probing the impact of linker functionalization.^30^ In contrast to monofunctional phenols, catechol provides a more sensitive platform to probe subtle changes in pore polarity and interaction strength because its vicinal hydroxyl groups promote stronger intermolecular interactions.^31^

Reported catechol uptakes in MOFs vary widely with pore size, surface chemistry, and host–guest interactions. Hydroxyl-functionalized frameworks are especially promising: Ma et al. reported a water-stable terbium-organic framework reaching 260 mg g^–1^,^26^ attributed to hydrogen bonding between framework hydroxyl groups and the ortho-dihydroxy functionality of catechol. Zr-based UiO-type frameworks, though widely studied for gas adsorption and separation, remain underexplored for catechol capture, particularly with rationally designed functional linkers. The present work addresses this gap using the Ugi four-component reaction to introduce amide-rich functionalities into UiO-67, establishing directional hydrogen bonds with catechol while preserving the robustness of Zr-based MOFs.

One of the central challenges to developing MOFs with affinity to a specific pollutant is the limited structural diversity and synthetic accessibility of functional dicarboxylate linkers.^32,33^ Conventional synthetic routes are often multistep and time-consuming, restricting access to chemically complex structures and limiting the systematic exploration of pore chemistry.^34–38^ In this regard, expanding the accessible chemical space is essential to achieve precise control over adsorption, catalysis, and guest interactions in MOFs.^39–43^

Multicomponent reactions (MCRs) represent a powerful and largely underexploited strategy for linker diversification in MOF chemistry. These reactions enable the rapid and atom-economical assembly of structurally complex molecules from simple building blocks in a single step, offering exceptional modularity and functional-group tolerance.^44–47^ In particular, the Ugi four-component reaction (Ugi-4CR) provides access to vast chemical space, with the number of possible products scaling combinatorially.^48–50^ Despite its widespread use in organic and medicinal chemistry, its application to the presynthetic design of MOF linkers remains significantly less explored.^51^

Here, we exploit the aniline functionality of a biphenyl-based dicarboxylate ester as a synthetic handle for linker modification via the Ugi multicomponent reaction.^52^ This approach combines an amine, an aldehyde, a carboxylic acid, and an isocyanide (Figure 1a), enabling direct access to a series of structurally diverse and chemically complex dicarboxylate ligands while preserving the geometry required for UiO-type framework assembly (Figure 1b). By varying individual components, a broad range of linker functionalities can be systematically introduced. In this way, dimethyl 2-amino-[1,1′-biphenyl]-4,4′-dicarboxylate is transformed into a family of Ugi-derived linkers compatible with zirconium-based MOF synthesis, enabling systematic control over pore functionality within the UiO-67 platform.

**Figure 1:**
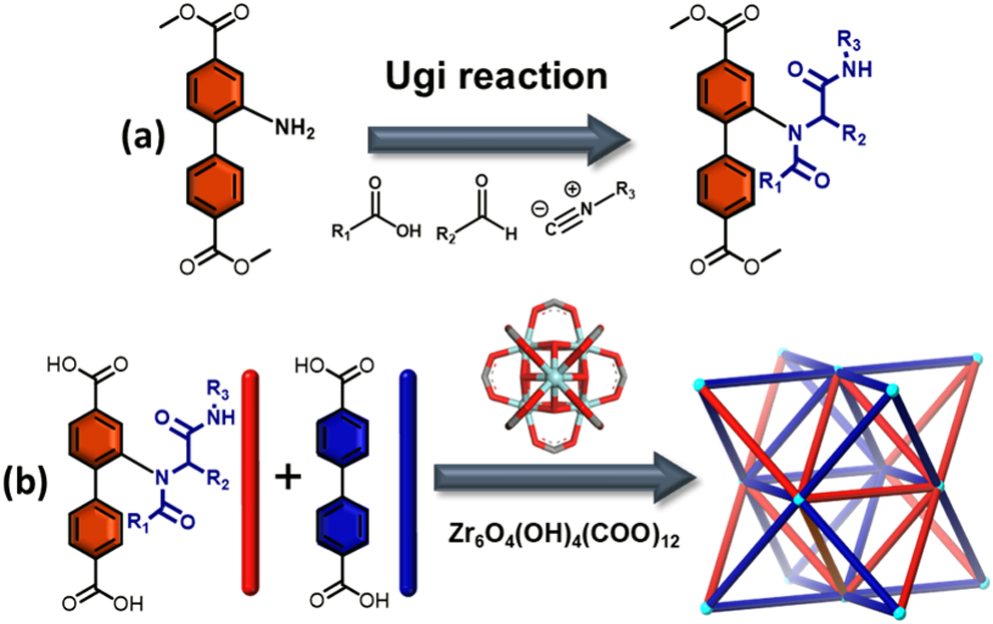
Overview of the presynthetic Ugi-based functionalization strategy employed in this work. (a) The Ugi four-component reaction U-4CR for the chemical modification of the aniline portion, (b) Mixed-linker approach to prepare a MOF isoreticular to UiO-67, featuring the Ugi functionalization.

The presynthetic functionalization strategy was selected rather than the postsynthetic modification because the Ugi four-component condensation is most effectively controlled in homogeneous solution, where all reactants are freely accessible. This route allows each functionalized linker to be isolated and fully characterized at the molecular level before framework assembly, and by simply varying the aldehyde, the acid and the isocyanide components, it grants modular access to a structurally diverse linker family from a single amino-dicarboxylate precursor, thereby expanding the accessible chemical space of UiO-type frameworks.

## 2 Experimental Section

### 2.1 Synthesis of Ugi-Derived Dicarboxylate Linkers

All chemicals and solvents were obtained commercially and used without further purification. Detailed synthetic procedures and characterization data are provided in the Supporting Information.

The first Ugi four-component reaction was carried out in methanol using benzaldehyde, acetic acid, *tert*-butyl isocyanide, and a biphenyl-based amino dicarboxylate ester. The reaction mixture was stirred at room temperature for 24 h to afford a peptide-like precursor incorporating amide functionalities and a quaternary carbon center. The resulting Ugi adduct was subjected to basic hydrolysis to yield the corresponding dicarboxylated ligand (**L-Ugi-1**). All products belonging to this set were isolated by acidification and filtration. All linkers produced using this approach were characterized by ^1^H and ^13^C NMR spectroscopy, fourier transform infrared spectroscopy (FT-IR) spectroscopy, high-resolution mass spectrometry, and solid-state ^13^C NMR spectroscopy (Figure 2).

**Figure 2:**
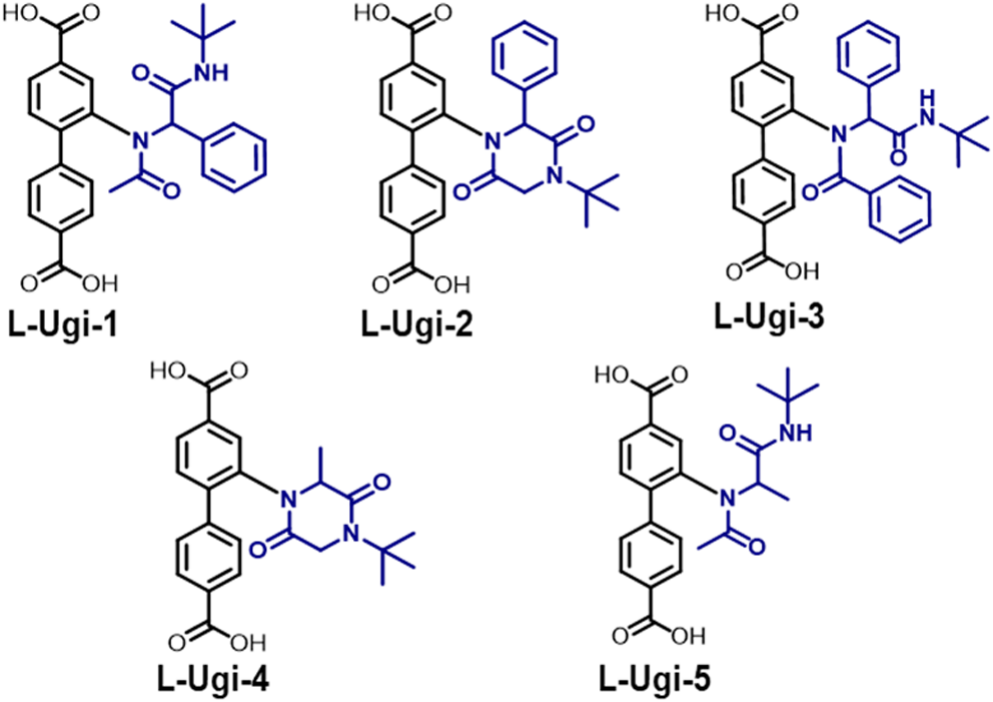
A series **(1–5)** of dicarboxylated ligands **(L-Ugi)** was prepared by using the Ugi multicomponent reaction.

### 2.2 Synthesis of UiO-67-Type MOFs

Once the Ugi-derived linkers were produced with good yields, the UiO-67-type MOFs were synthesized under solvothermal conditions using ZrCl_4_ as the metal precursor and DMF as the solvent in the presence of a modulating agent. Considering the potential steric bulk of the Ugi functionalities, synthesis parameters were optimized to preserve excellent crystallinity and phase purity. Among the conditions examined, the highest crystallinity was obtained using trifluoroacetic acid as the modulator at 120 °C for 72 h. The resulting solids were isolated by centrifugation, washed with fresh DMF, and activated prior to characterization. A series of materials, denoted MOFs **Ugi-XA** (X = 1–5), were obtained and characterized by PXRD, FT-IR spectroscopy, TGA, and solid-state ^13^C NMR spectroscopy.

### 2.3 Catechol Adsorption Experiments

Adsorption experiments were performed using catechol as a model dihydroxy aromatic compound. MOF samples (20 mg) were dispersed in 10 mL of a 2.5 mM catechol solution in acetonitrile and stirred at room temperature for 24 h. The suspensions were centrifuged, and the supernatants were analyzed by high-performance liquid chromatography (HPLC) using an external calibration curve. Adsorption capacities were calculated from the difference between initial and final concentrations and expressed as mg g^–1^. All experiments were performed under identical conditions to enable direct comparison between materials.

## 3 Results and Discussion

### 3.1 Structural Characterization of MOF Ugi-1A

The powder X-ray diffraction (PXRD) pattern of **MOF Ugi-1A** exhibited well-defined reflections that coincide with the Bragg positions reported for UiO-67, consistent with a three-dimensional cubic lattice constructed from Zr_6_O_4_(OH)_4_ nodes.^53^ The similarity in peak positions and relative intensities indicates that incorporation of the bulky Ugi-modified linker does not induce significant changes in lattice symmetry or long-range periodicity. To further support structural preservation, the experimental PXRD data was analyzed using Le Bail fitting with the cubic *Fm3̅m* space group. The refined lattice parameters for MOF **Ugi-1A** (*a* = 26.674(4) Å, *V* = 18 979(5) Å^3^) are in good agreement with those of pristine UiO-67 (*a* = 26.813(1) Å, *V* = 19 276(1) Å^3^) and previously reported values,^53–55^ indicating only a slight lattice contraction upon functionalization, corroborating the phase and structural integrity of the functionalized material. Altogether, these results confirm retention of the characteristic *fcu* topology and structural integrity of the framework.

All three materials display the reflection set expected for the cubic UiO-67 structure, with no low-angle superstructure reflections of correlated missing-cluster (*reo*) domains, which, together with elemental analyses consistent with predominantly linked formulations, indicates 12-connected *fcu* frameworks with a low defect concentration. Consistently, the porosity trends upon Ugi functionalization are opposite to those expected from defect incorporation, supporting pore blocking by the bulky linker, rather than missing-linker porosity, as the dominant structural phenomenon in MOF Ugi-1A. A detailed assessment of defect incorporation is provided in the Supporting Information.

The morphological features of MOF Ugi-1A were examined by optical microscopy alongside those of the parent UiO-67 material (Figure 3c). Both materials exhibit the characteristic octahedral morphology typical of UiO-type crystals; however, MOF Ugi-1A shows a slight loss of facet definition and increased surface roughness.

**Figure 3:**
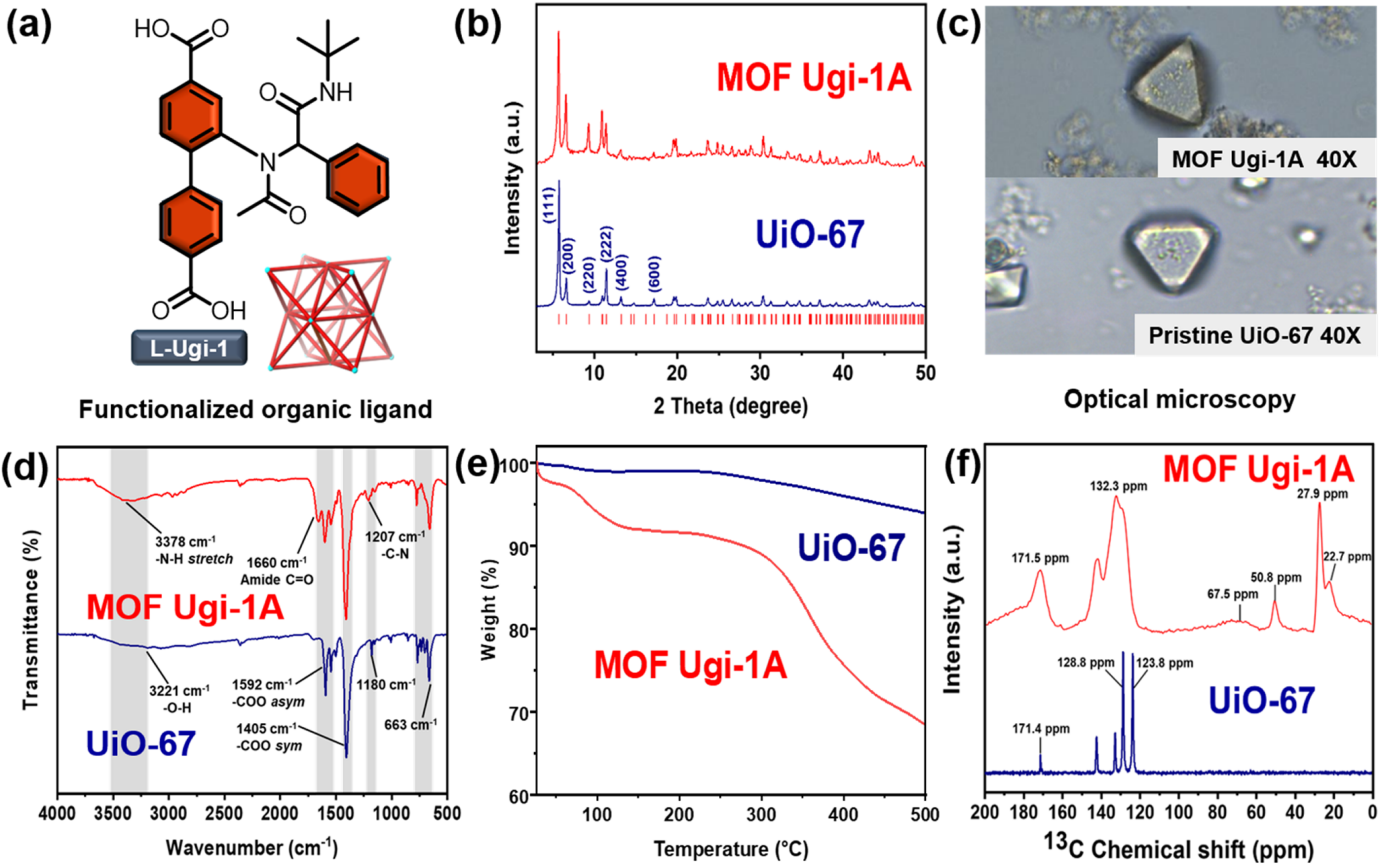
Comparison of the **MOF Ugi-1A** and **UiO-67**. (a) Organic linker used for the synthesis of **MOF Ugi-1A**, (b) PXRD, (c) Optical microscopy of **UiO-67** and **MOF Ugi-1A**, (d) FT-IR spectra, (e) TGA and (f) solid-state ^13^C NMR spectra.

Despite this well-defined crystal habit, attempts to obtain single crystals of sufficient size and quality for synchrotron analysis were unsuccessful; the framework structures were therefore validated by Rietveld refinement against the powder diffraction data (see Supporting Information). In addition, the molecular structure of the Ugi-functionalized linker DBPDC-Ugi-1 established by single-crystal X-ray diffraction (Figure S3) confirmed the connectivity and identity of the dicarboxylate building block prior to framework assembly.

Consequently, structural characterization was established through the convergent multitechnique approach described above. FT-IR spectroscopy (Figure 3d) further supports preservation of the UiO-67 framework after functionalization. Both MOF **Ugi-1A** and pristine UiO-67 exhibit a broad absorption band in the 3400–3200 cm^–1^ region, attributed to O–H stretching vibrations associated with μ_3_–OH groups of the Zr_6_ clusters and adsorbed water.^56,57^ Aromatic C–H stretching bands at 3060–3020 cm^–1^, together with the characteristic asymmetric and symmetric stretching modes of coordinated carboxylate groups at approximately 1592 and 1405 cm^–1^, respectively, are observed in both materials, confirming preservation of the UiO-type coordination environment.^58,59^

In contrast, the IR spectrum of the Ugi-functionalized ligand displays distinct additional features, including a broad N–H stretching band around 3378 cm^–1^, consistent with hydrogen-bonded amide groups, and new carbonyl vibrations in the 1650–1700 cm^–1^ region characteristic of amide functionalities. Additional absorption in 1207 cm^–1^ further supports the presence of C–N vibrations associated with the Ugi-derived moieties, while changes in the aromatic out-of-plane bending region are consistent with substitution effects on the biphenyl backbone.^60,61^

Thermogravimetric analysis (TGA) (Figure 3e) highlights the impact of Ugi functionalization on the thermal stability of the framework. Both materials exhibit the characteristic multistep mass-loss profile of Zr-based UiO-type MOFs, though it is significantly more pronounced in the modified MOF, including the removal of residual solvent and physisorbed species below 150 °C, followed by framework decomposition at higher temperatures.^62–64^ Thus, the pristine UiO-67 exhibits greater thermal robustness, with significant degradation occurring only above 500 °C. In contrast, the MOF **Ugi-1A** displays a mass loss between 250 and 450 °C, attributed to partial decomposition of the Ugi portions, containing thermally less stable C–N and C–O bonds.^65,66^ This behavior is consistent with previous reports on functionalized UiO-type frameworks and reflects the incorporation of flexible organic fragments that alter the organic–inorganic balance of the structure.^67^ The full TGA traces of UiO-67 and MOF Ugi-1A, together with the detailed assignment of the mass-loss events and the interpretation of the residual masses, are provided in the Supporting Information.

Solid-state ^13^C NMR spectroscopy provided direct insights into the local chemical and crystallographic environment within the functionalized framework^68,69^ (Figure 3f). The spectrum of pristine UiO-67 exhibits characteristic sharp resonances corresponding to carboxylate carbonyl carbons at 171.4 ppm and aromatic carbons at 142.5 and 123.8 ppm, in agreement with literature reports.^70,71^ Conversely, in the spectrum of MOF **Ugi-1A**, the carbonyl signal at 171.4 ppm is significantly broadened, likely due to strong homonuclear dipolar coupling arising from the proximity of the Ugi functionalities.^68^ Meanwhile, the initial aromatic carbon signals were observed, confirming the preservation of the biphenyl backbone within the UiO framework.^72^ Additional resonances observed at 67.5 and 50.8 ppm were assigned to tertiary and quaternary carbon centers of the Ugi adduct, while the intense signal at 27.9 ppm is characteristic of the methyl groups of the *tert*-butyl substituent. Finally, the signal present at approximately 22.7 ppm corresponds to the substituted methyl group in the adduct.^73–75^ Together, these features provide unambiguous spectroscopic evidence for the successful incorporation of the Ugi-functionalized linker into the UiO-67 framework.^76^

Elemental combustion analysis further corroborated the incorporation of the Ugi-functionalized linker (Supporting Information). The experimentally determined C, H, and N contents closely match the theoretical values, confirming the effectiveness of the synthetic approach.^77^ As expected, functionalized MOFs exhibit a marked increase in nitrogen content relative to pristine UiO-67, consistent with the presence of amide functionalities introduced by the Ugi reaction.^78^ This increase in nitrogen content serves as a quantitative indicator of linker inclusion.^79^

### 3.2 Structural Characterization of MOF Ugi-1B

Once we confirmed the successful incorporation of Ugi groups into MOFs, we considered it possible to further modulate their composition using a multivariate (MTV) approach.^80,81^ In principle, this variable composition would enable us to fine-tune the pore environment and chemical functionality of the material.^82,83^ As a result, the envisioned MTV-MOFs might exhibit properties not accessible in single linker systems, offering enhanced tuning of adsorption, catalytic, and separation behavior.^84–86^ To this end, the multivariate MOFs were planned to contain biphenyldicarboxylic acid (H_2_BPDC) as a second organic component, using an equimolar feed ratio (1:1) of both linkers.^87,88^ The desired mixed-linker materials labeled MOFs **Ugi-XB** (X = 1–5) were synthesized under the same optimized solvothermal conditions as those with pure Ugi linkers, and were characterized by PXRD, FT-IR spectroscopy, solid-state ^13^C NMR, thermogravimetric analysis, and nitrogen adsorption measurements, as provided in the Supporting Information. It should be noted that MOF Ugi-5B could not be obtained under the optimized conditions; characterization of the resulting solid indicated selective incorporation of H_2_BPDC without inclusion of L-Ugi-5, and this material was therefore excluded from further analysis.

For brevity, we include only the description of the MOF **Ugi-1B**, which was obtained by mixing the **Ugi-1A** linker with H_2_BPDC. Solid-state ^13^C NMR spectroscopy was again used to confirm the incorporation of the Ugi-functionalized linkers into the UiO-67-type frameworks and to probe the local chemical environment of the resulting materials (Figure 4a).^89,90^ One of the most significant differences between the **Ugi-1A** and **Ugi-1B** MOFs is the relative signal intensities in the aliphatic region, which are consistent with the lower Ugi functionalization content in **Ugi-1B** (Figure 4c).^89,90^

**Figure 4:**
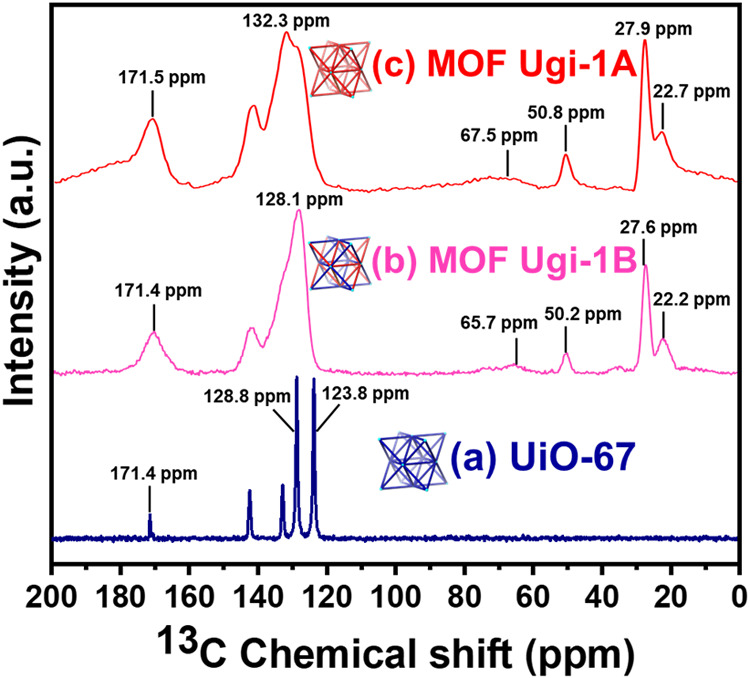
Solid-state ^13^C CPMAS NMR spectra of **(a) UiO-67**, **(b) MOF Ugi-1 B** and **(c) MOF Ugi-1 A**.

Furthermore, the carbon signals of the MOF **Ugi-1B** in the aromatic region are narrower, resulting from the presence of H_2_BPDC, which effectively dilutes the concentration of the bulky Ugi-functionalized linker within the framework, reducing homonuclear dipolar coupling (Figure 4b).^91,92^ Additionally, slight variations in the chemical shifts observed between **Ugi-1A** and **Ugi-1B** MOFs reflect differences in the local electronic environments of the linkers arising from the statistical distribution of functionalized and pristine ligands within the mixed-linker framework. The preservation of the characteristic resonances associated with the UiO-67 framework, together with the appearance of signals corresponding to the Ugi-derived moieties, confirms the successful incorporation of the functionalized linker while maintaining the structural integrity of the UiO-67 topology.^88,89^

PXRD showed that the mixed linker MOF **Ugi-1B** displays sharp reflections, which we attributed to the use of H_2_BPDC linker.^93,94^ Optical microscopy images (Figure 5c) further highlight the influence of the mixed-linker approach on crystal morphology.^95,96^ MOF **Ugi-1B** forms well-defined octahedral crystals with sharp facets, characteristic of highly ordered Zr-based UiO-type frameworks.^97^ This pronounced improvement in crystal morphology directly correlates with the enhanced crystallinity observed by PXRD, confirming that partial substitution with the biphenylcarboxylate linker promotes improved structural organization during framework assembly.^98,99^ Given these structural differences, the impact of Ugi functionalization on porosity was subsequently investigated.

**Figure 5:**
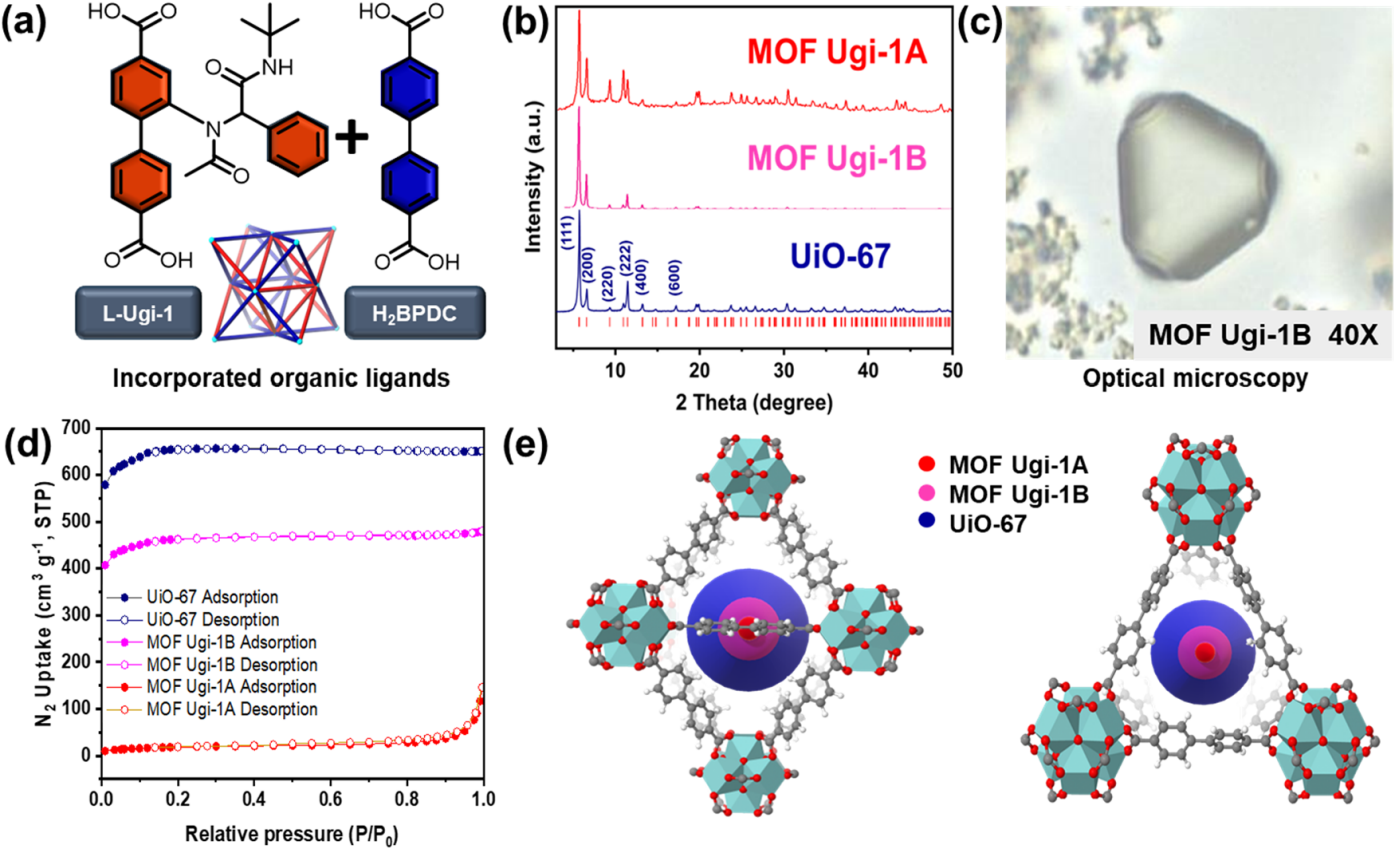
Characterization of MOF **Ugi-1B**: (a) organic ligands, (b) PXRD, (c) Optical microscopy, (d) Nitrogen adsorption, and (e) Approximate decrease in pore size compared to **UiO-67**.

### 3.3 Textural Properties (N_2_ Adsorption)

To demonstrate the extent of Ugi functionalization and the mixed-linker approach, we measured and compared N_2_ adsorption–desorption isotherms and corresponding textural parameters for MOFs UiO-67, **Ugi-1A**, and **Ugi-1B** (Figure 5d).^100^ Pristine UiO-67 exhibits a typical type I isotherm with a pronounced uptake at low relative pressures, consistent with a highly microporous structure.^94^ Upon incorporation of the Ugi-functionalized linker, a progressive decrease in N_2_ uptake is observed, reflecting reduced pore accessibility arising from the presence of bulky organic fragments within the framework.^101,102^

Accordingly, the Brunauer–Emmett–Teller (BET) surface area decreases from 2000 m^2^ g^–1^ for UiO-67 (consistent with the reported value), to 1417 m^2^g^–1^ for MOF **Ugi-1B** and is drastically reduced to 63 m^2^ g^–1^ for MOF **Ugi-1A**.^103,104^ A similar trend is observed for the Langmuir surface area (2905 → 2063 → 97 m^2^ g^–1^) and total pore volume (1.00 → 0.73 → 0.11 cm^3^ g^–1^).^105,106^ These results clearly indicate that increasing incorporation of the Ugi-derived linker progressively restricts internal porosity, as schematically illustrated in (Figure 5e).^107,108^

The average pore diameter follows an opposite trend. While pristine UiO-67 displays an average pore diameter of ∼2.6 nm, MOF **Ugi-1B** exhibits a modest increase to 3.3 nm, and MOF **Ugi-1A** shows a markedly larger apparent pore diameter of 22.3 nm.^109,110^ This apparent pore expansion, together with the broader pore size distribution and the presence of hysteresis at intermediate relative pressures, suggests the formation of secondary mesoporosity.^111–113^ Such features are commonly associated with defect-induced cavities, partial pore blockages, or interparticle voids rather than with the uniform enlargement of the intrinsic microporous channels.^114,115^

A detailed analysis of the pore size distribution, confirming that this apparent enlargement arises from external-framework voids rather than from expansion of the intrinsic cages, is provided in the Supporting Information.^116^

### 3.4 Catechol Adsorption Studies

Catechol adsorption studies were carried out to investigate how Ugi-derived functionalization modulates host–guest interactions in UiO-67 frameworks. Quantitative HPLC analysis revealed a clear dependence of adsorption performance on both the nature and extent of linker functionalization. As shown in (Figure 6 (a)), **MOF Ugi-1B** exhibited the highest catechol uptake (37.7 mg g^–1^), followed by pristine **UiO-67** (30.3 mg g^–1^), whereas **MOF Ugi-1A** showed significantly lower adsorption capacity (19.3 mg g^–1^).

**Figure 6:**
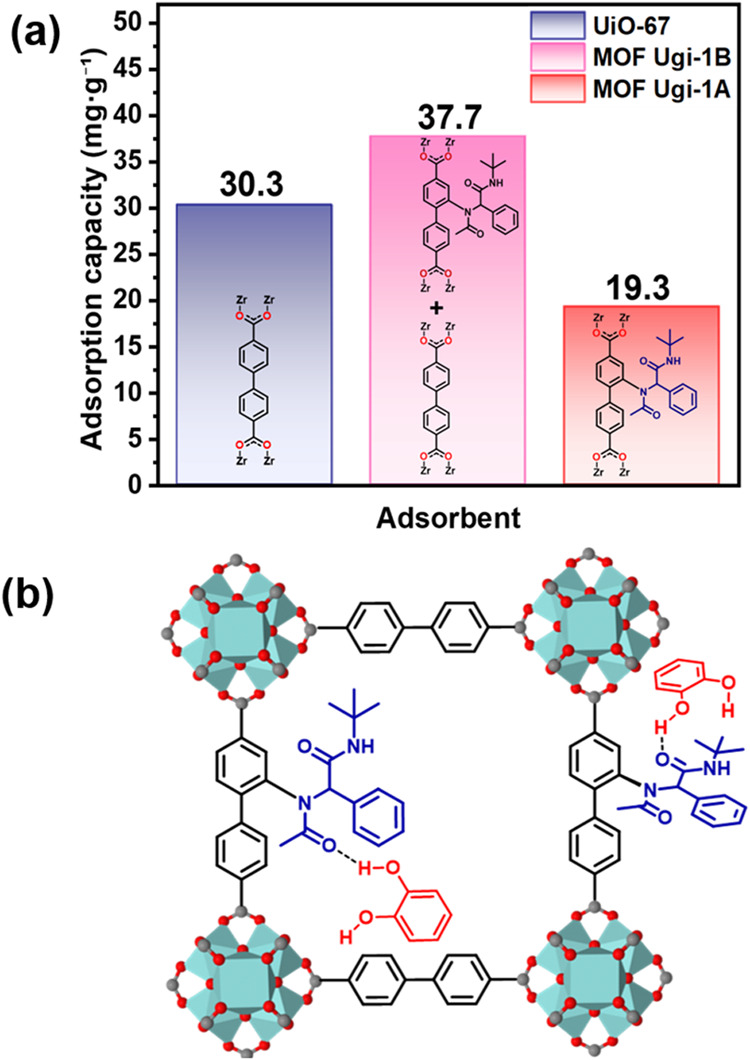
(a) Catechol adsorption capacity of UiO-67, MOF Ugi-1B and MOF Ugi-1A, with the corresponding linker structures shown for each material. (b) Schematic representation of catechol adsorption within the Ugi-functionalized UiO-67 framework.

The uptake reported here (37.7 mg g^–1^ for MOF Ugi-1B) is a subsaturating measurement in acetonitrile, a hydrogen-bond-accepting solvent that provides no solvophobic driving force and thus isolates specific host–guest interactions; even under these demanding conditions it matches or exceeds several aqueous-phase adsorbents (e.g., TiO_2_, 11.2 mg g^–1^; multiwalled carbon nanotubes, 14.2 mg g^–1^). Beyond capacity, the Zr-based platform offers decisive practical advantages over rare-earth frameworks: the strong Zr–O bonding in Zr_6_ clusters confers high thermal and hydrolytic robustness suited to aqueous or chemically demanding environments, while zirconium is far more abundant and available as a low-cost precursor (ZrCl_4_) than other metals such as terbium. Finally, the Ugi multicomponent strategy provides a modular synthetic handle for tuning functionality that a single-linker material does not.

The enhanced adsorption observed for **MOF Ugi-1B** suggests that an intermediate degree of functionalization provides the most favorable balance between pore accessibility and specific host–guest interactions. Catechol possesses two phenolic hydroxyl groups with reported *pK*_a_ values of approximately 9.2 and 13.0, indicating that under the experimental conditions it is expected to remain predominantly in its neutral form.^117^

Consequently, adsorption is likely governed primarily by noncovalent interactions rather than electrostatic effects. In the Ugi-functionalized frameworks, the amide groups introduced through the multicomponent linker may serve as hydrogen-bond acceptors for the phenolic OH groups of catechol (Figure 6 (b)), while the aromatic rings present in both the framework and the guest molecule can contribute additional π–π interactions.^72^ These cooperative interactions provide adsorption sites that are absent in pristine **UiO-67** and likely account for the improved catechol uptake observed for **MOF Ugi-1B**; spectroscopic evidence for this hydrogen-bonding interaction, from FTIR experiments on the catechol-loaded framework before and after adsorption, is provided in the Supporting Information.

The influence of pore size and architecture can be rationalized by considering the molecular dimensions of catechol, estimated from its crystal structure geometry to be approximately 7.8 × 7.1 × 3.4 Å. Comparison with the pore window apertures of UiO-67 (ca. 6 and 8 Å for tetrahedral and octahedral windows, respectively) indicates that catechol can access the larger cavities of the framework but may experience steric constraints during diffusion through narrower windows.

Therefore, efficient adsorption requires not only the presence of favorable interaction sites but also sufficient preservation of the intrinsic microporous network. The dimensions of catechol approach the size of the octahedral pore windows, implying that even moderate reductions in accessible pore volume or window accessibility caused by linker functionalization can significantly influence molecular transport within the framework.^118^

This relationship is reflected in the textural properties of the three materials. Pristine **UiO-67** possesses a high pore volume (1.00 cm^3^ g^–1^) and an average pore diameter of 2.6 nm, providing adequate accessibility for catechol molecules but lacking functional groups capable of establishing strong directional interactions, resulting in a moderate adsorption capacity of 30.3 mg g^–1^. In contrast, **MOF Ugi-1B** retains a largely accessible pore system, as evidenced by its average pore diameter of 3.3 nm and pore volume of 0.73 cm^3^ g^–1^, while simultaneously introducing amide-containing adsorption sites. The combination of preserved pore accessibility and enhanced intermolecular interactions results in the highest catechol uptake observed (37.7 mg g^–1^).

A different behavior is observed for **MOF Ugi-1A**, although this material contains a higher density of Ugi-derived functionalities that could potentially promote hydrogen-bonding interactions with catechol, extensive incorporation of the bulky linker significantly reduces the accessible microporosity. The drastic decrease in BET surface area (63 m^2^ g^–1^) and pore volume (0.11 cm^3^ g^–1^), together with the apparent increase in pore diameter to 22.3 nm, indicates that the measured porosity is dominated by interparticle mesoporosity rather than the intrinsic microporous network of the framework. As a result, diffusion and access to internal adsorption sites become severely restricted, and the loss of accessible porosity outweighs any potential gain arising from the higher density of functional groups, leading to the lowest catechol uptake observed (19.3 mg g^–1^).

Overall, the adsorption data demonstrate that catechol uptake is governed by a balance between chemical affinity and pore accessibility. Insufficient functionalization provides limited opportunities for specific host–guest interactions, whereas excessive functionalization compromises transport through the porous network due to steric congestion and partial pore blockage. The superior performance of **MOF Ugi-1B** therefore arises from an optimal combination of accessible pore space and strategically distributed amide-containing adsorption sites. These results highlight that maximizing adsorption performance in functionalized **UiO-67** materials requires not the highest density of functional groups, but rather a balance between framework accessibility and the availability of interaction sites. The multivariate mixed-linker strategy offers a modular approach to achieve this balance by simultaneously tuning pore chemistry and pore accessibility.

### 3.5 Post-Adsorption Characterization

The structural integrity of the MOFs after catechol adsorption was evaluated by PXRD. As shown in (Figure 7), the diffraction patterns of **Catechol@UiO-67** and **Catechol@MOF Ugi-1B** remain consistent with the corresponding as-synthesized materials, confirming that the crystalline framework is largely preserved after the adsorption process. The retention of the characteristic low-angle reflections associated with the UiO-67 topology indicates that catechol uptake does not induce significant structural degradation in these materials under the adsorption conditions employed.^96^ Minor variations in relative peak intensities were observed, which are attributed to guest incorporation within the pore environment and changes in electron density within the pore cavities following catechol uptake.^96^

**Figure 7:**
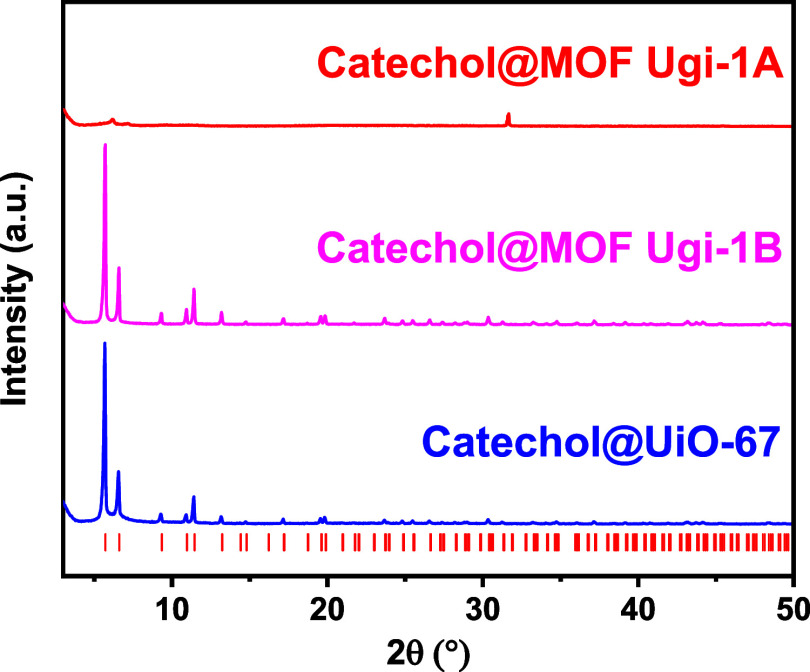
PXRD patterns of **Catechol@UiO-67**, **Catechol@MOF Ugi-1A**, and **Catechol@MOF Ugi-1B** after catechol adsorption.

In contrast, **Catechol@MOF Ugi-1A** shows a pronounced loss of diffraction intensity and disappearance of the characteristic Bragg reflections after the adsorption process. The resulting broad, nearly featureless diffraction profile indicates a substantial loss of long-range crystalline order, suggesting a severe loss of crystallinity and possible partial structural collapse upon catechol incorporation.^119^ This behavior is likely associated with the complete incorporation of the bulky Ugi-functionalized linker throughout the structure, which may reduce framework robustness and increase susceptibility to structural destabilization during adsorption.^119^

## 4 Conclusions

This study demonstrates that the Ugi four-component reaction is an effective presynthetic strategy for the modular modification of UiO-67 frameworks. The incorporation of structurally complex Ugi-derived linkers while preserving the characteristic *fcu* topology highlights the robustness and synthetic versatility of Zr-based MOFs. Moreover, the modular nature of multicomponent reactions enables rapid diversification of pore environments, providing a versatile platform for the rational engineering of pore chemistry.

However, increasing incorporation of bulky Ugi-derived fragments leads to reduced crystallinity, decreased porosity, and diminished adsorption performance, as observed for **MOF Ugi-1A**. In contrast, the mixed-linker **MOF Ugi-1B** achieves an optimal balance between pore accessibility and chemical functionality, exhibiting the highest catechol uptake among the materials studied. These findings underscore the delicate interplay between structural accessibility and pore functionalization, demonstrating that controlled multivariate linker incorporation is an effective strategy for tuning host–guest interactions in UiO-type MOFs. More broadly, this work establishes multicomponent chemistry as a promising approach for expanding the chemical space of functional porous materials with tunable host–guest properties.

## Supplementary Material



## Notes

Experimental procedures; PXRD patterns; FT-IR spectra; TGA curves; nitrogen adsorption data; HPLC chromatograms; and NMR spectra (PDF)
