# Supplementary material for: Expansion of the Chemical Space in UiO-Type Metal–Organic Frameworks via the Ugi Four-Component Reaction for Tuned Pore Accessibility
Source: Inorg Chem. 2026 Jul 24;65(31):18185–95. doi: 10.1021/acs.inorgchem.6c02585 (PMC13463678; doi:10.1021/acs.inorgchem.6c02585)
Supplement: Supplementary file 1 [file ic6c02585_si_001.pdf]

Supporting Information for

Expansion of the Chemical Space in UiO-Type Metal-Organic Frameworks via the Ugi Four-Component Reaction for Tuned Pore Accessibility

Erik Uribe Vega,[1] Ma Carmen Garcia-González,[1*] Axel Uribe Vega,[1] Salvador López Morales,[2] Joelis Rodríguez Hernández,[3] Erick Hernández-Santiago,[1] Eréndira García-Ríos,[1] Luis D. Miranda,[1] Braulio Rodríguez-Molina[1]*

[1]*Instituto de Química, Universidad Nacional Autónoma de México, Circuito Exterior, Ciudad Universitaria, 04510 Ciudad de México, México.*
[2]*Instituto de Investigaciones en Materiales, Universidad Nacional Autónoma de México, Circuito Exterior, Ciudad Universitaria, 04510 Ciudad de México, México.*
[3]*Centro de Investigación en Química Aplicada, Blvd. Ing. Enrique Reyna Hermosillo No. 140, Col. San José de los Cerritos, 25294, Saltillo, Coahuila, México.*

*** Corresponding Authors:**

brodriguez@iquimica.unam.mx, carmen.garcia@iquimica.unam.mx

Table of Contents

1. Chemicals and Instrumentation

1.1 Chemicals……………………………………………………………...………….………….…......4
1.2 Instrumentation ………………………………………………………………………………….4

2. Synthesis of Ugi adducts and organic linkers
2.1 Synthesis of DBPDC-NO_2……………………………………….……………………….…5
2.2 Synthesis of DBPDC-NH_2…………………………………………………………..….5
2.3 Synthesis of H_2BPDC………………………………………………………….................5
2.4 General procedure for the synthesis of DBPDC-Ugi adducts ……….……….…………...…….6
2.4.1 Synthesis of DBPDC-Ugi-1……………………………………………………………………7
2.4.2 Synthesis of DBPDC-Ugi-2……………………………………………………................7
2.4.3 Synthesis of DBPDC-Ugi-3……………………………………………………................8
2.4.4 Synthesis of DBPDC-Ugi-4………………………………………….……………………8
2.4.5 Synthesis of DBPDC-Ugi-5……………………………………………………................8
2.5 General procedure for the synthesis of organic linkers……………………………..................9
2.5.1 Synthesis of H_2BPDC-Ugi-1…………………………….………………...……..……….…9
2.5.2 Synthesis of H_2BPDC-Ugi-2………………………………………………..….……………9
2.5.3 Synthesis of H_2BPDC-Ugi-3 …………………………………………...……………..10
2.5.4 Synthesis of H_2BPDC-Ugi-4 ………………………………………………....................10
2.5.5 Synthesis of H_2BPDC-Ugi-5 ………………………………………………………….....10

3. Ugi adducts and organic linkers characterizations
3.1 DBPDC Ugi adducts ^{1}H and ^{13}C NMR spectra……….…………………………..…...11-17
3.2 Ugi functionalized linkers ^{1}H and ^{13}C NMR spectra……………………………..……….18-22
3.3 DPBDC Ugi adducts and organic linkers FT-IR spectra…………………………..……..……23
3.4 Solid-state ^{13}C CPMAS NMR spectra of organic linkers……………………………………….24

4. MOFs Synthetic Procedures

4.1 Synthesis of UiO-67……………………………………………………………………..………25
4.2 General procedure for the synthesis of Ugi-functionalized MOFs…………………….…..…25
4.3 General procedure for the synthesis of Ugi MTV-MOFs…………………….……………...26

5. Characterization of MOFs

5.1 Ugi-functionalized MOFs characterizations…………………………………………………......27-29
5.2 Ugi MTV-MOFs characterizations……………………………………………………………29-31
5.3 Le Bail Refinement of the Ugi-1 Series of MOFs……………………………………………..31-32
5.4 Rietveld Refinements of MOF Ugi-1 Series……………………………………………………33
5.5 Nitrogen adsorption analysis ……………………………………………………...…..............34-35
5.6 Reduction in accessible porosity ………..………………………………….……………………...36
5.7 Elemental analysis of MOFs ……………………………………...………………….................37
5.8 MOFs digestion and ^{1}H NMR analysis………………………………………...…………...38-40

6. Catechol Adsorption and HPLC Quantification

6.1 Calibration curve of catechol ……………………………………………………………………40-41
6.2 Quantification methodology ……………………………………………………………………...41
6.3 Adsorption experiments and HPLC analysis……………………………………………………...41-42

7. Post-Adsorption Characterization

7.1 ATR-FTIR spectra of Catechol-Loaded MOFs…………………………………………………......42-43
7.2 TGA Analysis of Catechol-Loaded MOFs…………………………………………………………44-45
7.3 Morphological Characterization of UiO-67-Based Materials after Catechol Adsorption…………….……45

8. References

1. Chemicals and instrumentation

1.1 Chemicals

Dimethyl biphenyl-4,4′-dicarboxylate, nitric acid (65 wt % in H_2O), tin(II) chloride dihydrate ($SnCl_2·2H_2O$, 98%), 2,2,2-trifluoroethanol (99%), benzaldehyde (≥99%), tert-butyl isocyanide (98%), ethanol (≥99.8%), trifluoroacetic acid (99%), and zirconium(IV) chloride ($ZrCl_4$, 99.5%) were purchased from Sigma-Aldrich. Acetic acid (glacial, ≥99.7%), sulfuric acid (95–98%), methanol (≥99.9%), hydrochloric acid (37 wt % in H_2O), sodium hydroxide (≥98%), chloroform (≥99.8%), tetrahydrofuran (THF, ≥99.5%), and dimethylformamide (DMF, ≥99.8%) were purchased from Tecsiquim. Deionized water was obtained from a Milli-Q water purification system (18.2 MΩ·cm). All chemical reagents and solvents were used as received without further purification unless otherwise specified.

1.2 Instrumentation

Sonication.

Sonication was carried out using an ultrasonic bath operating at a frequency of 40 kHz. Samples were sonicated under room conditions for the required time (typically 5–15 min) to ensure adequate dispersion or dissolution.

Fourier-Transform Infrared (FT-IR) Spectroscopy.

Infrared spectra were collected using a Bruker Tensor series FT-IR spectrometer equipped with a standard DTGS detector. Spectra were recorded in the range of 4000–400 cm^{-1} using attenuated total reflectance (ATR) or KBr pellet techniques, as appropriate.

Thermogravimetric Analysis (TGA).

Thermogravimetric analyses were performed using a Netzsch STA 449 F3 Jupiter thermal analyzer. Measurements were carried out over a temperature range of 25–500 °C under a continuous nitrogen flow, using a heating rate of 10 °C min^{-1}. An empty Al_2O_3 crucible was used as the reference.

Powder X-ray Diffraction (PXRD).

Powder X-ray diffraction patterns were collected on a Bruker D8 Advance diffractometer equipped with a LynxEye detector using Cu Kα radiation (λ = 1.5406 Å). Data were acquired at a tube voltage of 30 kV and a current of 25 mA, with a step size of 0.03°, a counting time of 0.3 s per step, and a 2θ range of 4–80°.

Solution Nuclear Magnetic Resonance (NMR) Spectroscopy.

^1H and ^{13}C NMR spectra were recorded at 25 °C on Bruker Avance III (400 MHz) and JEOL (300 MHz) spectrometers. Chemical shifts (δ) are reported in parts per million (ppm) and referenced to residual solvent signals ($CDCl_3$: δ = 7.26 ppm for ^1H and δ = 77.00 ppm for ^{13}C) and (DMSO-d_6: δ = 2.50 ppm for ^1H and δ = 39.52 ppm for ^{13}C).

Optical Microscopy.

Optical microscopy images were obtained using an Olympus BX43 microscope. Samples were dispersed in ethanol or acetone and drop-cast onto glass slides prior to analysis.

Gas Sorption Isotherms.

Nitrogen adsorption isotherms were measured using a Micromeritics ASAP 2020 surface area analyzer at 77 K using a liquid nitrogen bath. Prior to analysis, samples (ca. 30 mg) were degassed under vacuum (< 1.3 Pa) at 50 °C for 12 h. After degassing, free-space volumes were determined using helium. Specific surface areas were calculated using the Brunauer–Emmett–Teller (BET) method.[1]

Solid-State ^{13}C MAS NMR Spectroscopy.

Room-temperature magic angle spinning (MAS) ^{13}C solid-state NMR experiments were performed on a Bruker 500 MHz spectrometer equipped with an Avance III HD console and a 4 mm double-resonance ^{1}H–^{13}C MAS probe. Spectra were acquired using cross-polarization (CP) to enhance signal sensitivity.[2] CP matching conditions were optimized using an external adamantane standard. Typical CP contact times of 2 ms were employed, with 65,536 scans accumulated per spectrum and a recycle delay of 1.0 s. The MAS spinning frequency was set to 10 kHz. Linearly ramped ^{1}H spin-lock fields (90–100%) were applied to improve Hartmann–Hahn matching.[3] SPINAL-64 heteronuclear decoupling with a ^{1}H RF field of approximately 100 kHz was used during acquisition.[4]

High-Resolution Mass Spectrometry (HRMS).

High-resolution mass spectra were recorded using a JEOL AccuTOF JMS-T100LC mass spectrometer equipped with a source operating in positive ion mode.

High-Performance Liquid Chromatography (HPLC).

HPLC analyses were performed using an Agilent 1260 Infinity II system equipped with a diode array UV–Vis detector. Separations were carried out on an Eclipse Plus C18 column. The mobile phase consisted of acetonitrile and water (0.1% acetic acid) under isocratic conditions. The flow rate was 0.2 mL min^{-1}, and detection was performed at 275 nm. Samples were prepared in acetonitrile.

2. Synthesis of Ugi adducts and organic linkers

2.1 Synthesis of Dimethyl 2-nitro-[1,1′-biphenyl]-4,4′-dicarboxylate (DBPDC-NO_2)

Dimethyl [1,1′-biphenyl]-4,4′-dicarboxylate (1.00 g, 3.7×10^{-3} mol) was dissolved in concentrated H_2SO_4 (5 mL) under stirring in a round-bottom flask. The solution was cooled in an ice bath, and a sulfonitric mixture (0.3 mL of HNO_3 and 1.5 mL of H_2SO_4) was added dropwise. The reaction mixture was stirred for 1 h at 0 °C and then poured onto crushed ice to induce precipitation. The resulting solid was collected by vacuum filtration and dried with Na_2SO_4 to afford DBPDC-NO_2 as a light yellow powder (730 mg, 73%).[5]

2.2 Synthesis of Dimethyl 2-amino-[1,1′-biphenyl]-4,4′-dicarboxylate (DBPDC-NH_2)

Dimethyl 2-nitro-[1,1′-biphenyl]-4,4′-dicarboxylate (730 mg, 2.3×10^{-3} mol) was dissolved in methanol (50 mL), and tin powder (3.6 g) and aqueous HCl (1 M, 10 mL) were added. The resulting suspension was heated under reflux with vigorous stirring for 2 h. After cooling, the reaction mixture was poured onto crushed ice and basified with aqueous NaOH (1 M) to pH 10. The precipitated solid was collected by filtration and extracted with hot ethyl acetate. Insoluble byproducts were removed by filtration through Celite, and the solvent was removed under reduced pressure, yielding DBPDC-NH_2 as a light yellow powder (664 mg, 91%).[6]

2.3 Synthesis of [1,1′-biphenyl]-4,4′-dicarboxylic acid (H_2BPDC)

[1,1′-Biphenyl]-4,4′-dicarboxylate dimethyl ester (500 mg, 2.06×10^{-3} mol) was placed in a 100 mL round-bottom flask. NaOH (1.65 g, 41 mmol), THF (30 mL), ethanol (10 mL), and Milli-Q water (10 mL) were added sequentially, and the flask was sealed with a Teflon cap. The reaction mixture was heated to reflux with stirring for 12 h. After cooling, the product was precipitated by dropwise addition of aqueous HCl until pH = 1. The solid was collected by vacuum filtration and washed with water to afford H_2BPDC as a white powder (450 mg, 92%).[7]

2.4 General procedure for the synthesis of DBPDC-Ugi adducts

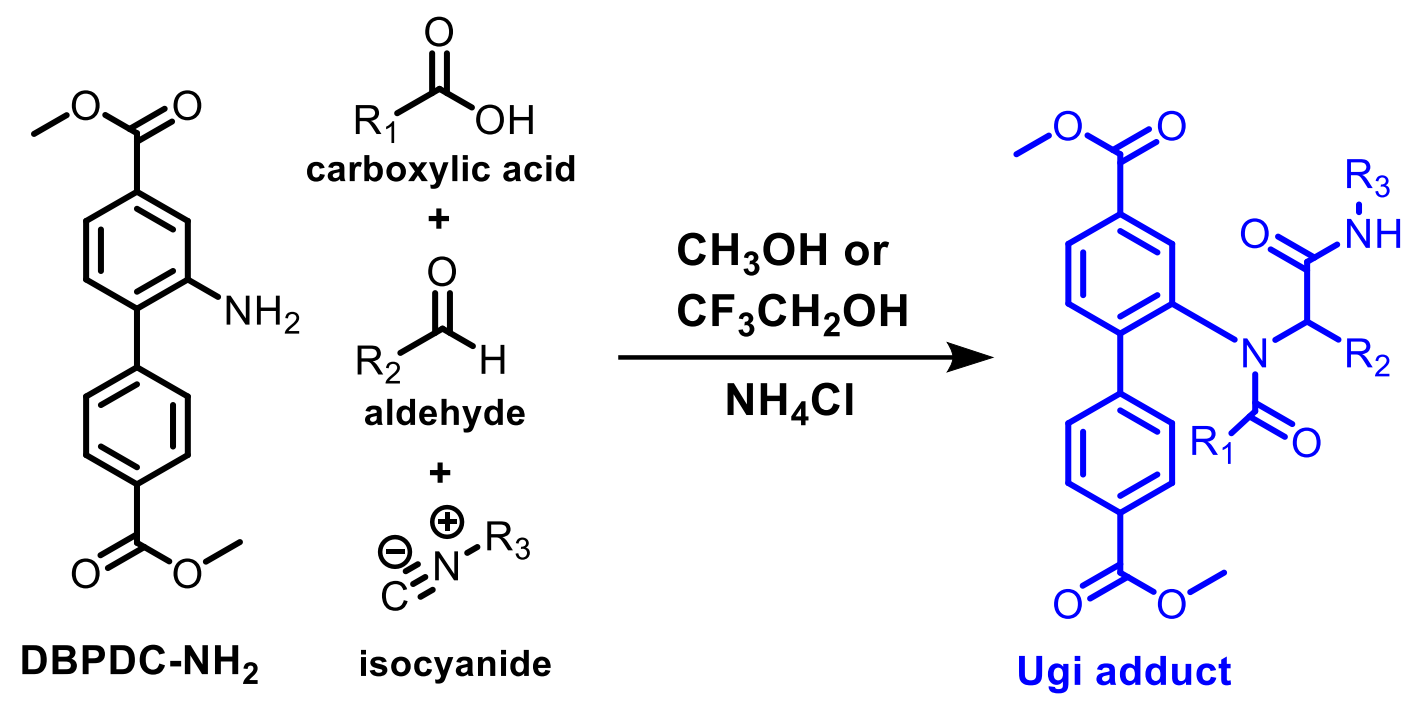

Scheme S1. General synthesis of Ugi adducts from DBPDC-NH_2.

Dimethyl 2-amino-[1,1′-biphenyl]-4,4′-dicarboxylate (DBPDC-NH_2) (200 mg, 0.701 mmol) was placed in a 50 mL round-bottom flask, followed by the addition of methanol (10 mL) and equimolar amounts of aldehyde, carboxylic acid, and isocyanide (1.0 equiv each). Finally, NH_4Cl (20 mg) was added as a catalyst. The flask was sealed with a Teflon stopper, and a nitrogen atmosphere was maintained through a needle connection. The reaction mixture was stirred at room temperature until complete consumption of the starting material, as monitored by thin-layer chromatography (TLC) using hexane/ethyl acetate (8:2) as the eluent. The crude product was extracted with dichloromethane (3 × 20 mL) and purified by ultrafast column chromatography using hexane/ethyl acetate as the eluent. The purified compound was dissolved in dichloromethane (10 mL), and the solvent was removed under reduced pressure at room temperature. This synthetic procedure was adapted from previously reported protocols for Ugi adduct formation. [8]

Condition optimization study

To optimize the reaction conditions for Ugi adduct formation, a systematic study was conducted by varying key experimental parameters, including the amount of the amine-containing dicarboxylated linker, the solvent, the nature and loading of the catalyst, the reaction temperature, and the reaction time. All reactions were performed under otherwise identical conditions, and products were isolated following the same workup and purification procedures. The yields reported in Table S1 correspond to isolated yields and represent the average of at least two independent experiments. This study enabled the identification of the optimal reaction conditions, improving both the efficiency and reproducibility of the Ugi adduct formation.

Table S1. Optimization of the reaction to obtain the DBPDC-Ugi-1 adduct.

Initial amount of DBPDC-NH_2(g)	Solvent	Catalyst	Amount of catalyst (g)	Temperature (°C)	Reaction time (h)	Yield (%)
0.1	Methanol	----	----	25°C	24	----
0.1	Methanol	NH_4Cl	0.01	25°C	96	**58**
0.1	Methanol	NH_4Cl	0.01	25°C	60	**67**
0.1	Methanol	$Sc(OTf)_3$	0.01	25°C	24	**23**
0.1	Methanol	NH_4Cl	0.01	45°C	72	**10**
0.1	Methanol	NH_4Cl	0.01	25°C	72	**81**
0.1	Toluene	$InCl_3$	0.01	25°C	48	**38**
0.1	Trifluoroethanol	----	----	25°C	72	**50**

Signal Duplication Due to Rotameric Conformations

The additional minor resonances observed in the ^{1}H and ^{13}C NMR spectra are attributed to the presence of conformational isomers in solution. Ugi-derived products typically contain amide or pseudopeptidic moieties that exhibit restricted rotation around the C–N bond due to its partial double-bond character. This restricted rotation can generate slowly interconverting rotamers on the NMR timescale, leading to signal duplication or broadening as chemically equivalent nuclei experience slightly different magnetic environments. Such behavior has been widely reported for Ugi reaction products and structurally related amide-containing systems.[9,10]

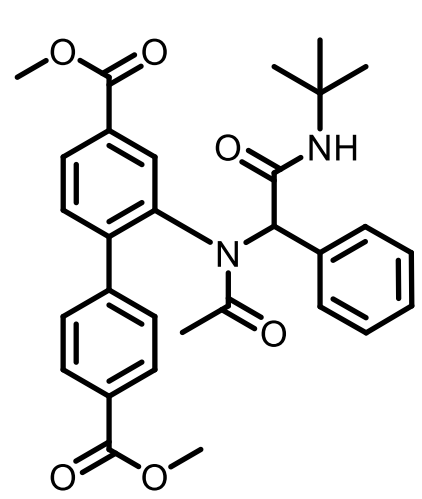

DBPDC-Ugi-1

Synthesis of dimethyl 2-(N-(2-(tert-butylamino)-2-oxo-1-phenylethyl)acetamido)-[1,1'-biphenyl]-4,4'-dicarboxylate (DBPDC-Ugi-1). Dimethyl 2-amino-[1,1'-biphenyl]-4,4'-dicarboxylate (DBPDC-NH_2) (200 mg, 0.701 mmol) was placed together with 10 mL of methanol and benzaldehyde (71.5 µL, 0.701 mmol), acetic acid (40.1 µL, 0.701 mmol), and *tert*-butyl isocyanide (79.3 µL, 0.701 mmol) in a 50 mL round-bottom flask. The reaction was stirred for 24 h at room temperature. The product was purified by flash column chromatography in a 7:3 ratio (hexane/AcOEt). (241 mg, 67%), white solid. mp 167-170 °C.

HRMS (DART+) calculated for $C_{30}H_{33}N_2O_6$ [M + H]$^{+}$ 517.2339; found 517.2319. **^{1}H NMR** (400 MHz; $CDCl_3$) δ: 8.59 (d, *J* = 4 Hz, 1H), 7.91 (d, *J* = 8 Hz, 2H), 7.21 (d, *J* = 8 Hz, 1H), 7.12 – 7.05 (m, 2H), 6.89 (t, *J* = 8 Hz, 2H), 6.83 (d, *J* = 8 Hz, 2H), 6.51 (d, *J* = 8 Hz, 2H), 5.45 (s, 1H), 5.28 (s, 1H), 3.97 (d, *J*= 8 Hz , 6H), 2.06 (s, 3H), 1.25 (s, 9H). **^{13}C NMR** (100 MHz, $CDCl_3$) δ: 171.9, 169.1, 166.9, 166.2, 144.3, 142.4, 138.1, 133.9, 132.8, 131.1, 130.8, 130.6, 130.1, 129.5, 128.9, 128.4, 128.1, 65.7, 52.6, 52.4, 51.7, 28.7, 24.3. **IR (ATR, νmax, cm^{-1}):** 3298, 1720, 1647, 1281, 1113, 762, 698, 547.

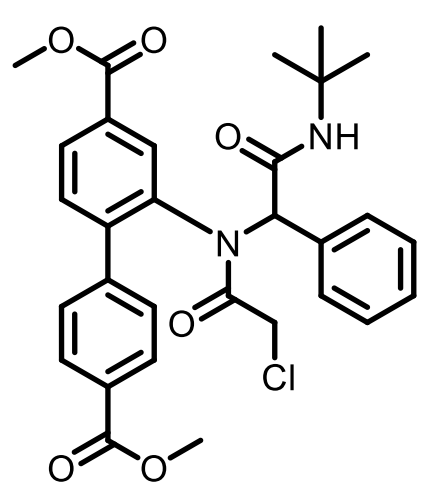

DBPDC-Ugi-2

Synthesis of dimethyl 2-(N-(2-(tert-butylamino)-2-oxo-1-phenylethyl)-2-chloroacetamido)-[1,1'-biphenyl]-4,4'-dicarboxylate (DBPDC-Ugi-2). Dimethyl 2-amino-[1,1'-biphenyl]-4,4'-dicarboxylate (DBPDC-NH_2) (200 mg, 0.701 mmol) was placed together with 10 mL of methanol and benzaldehyde (71.5 µL, 0.701 mmol), chloroacetic acid (66.24 mg, 0.7 mmol), and *tert*-butyl isocyanide (79.3 µL, 0.701 mmol) in a 50-mL round-bottom flask. The reaction was stirred for 96 h at room temperature. The product was purified by flash column chromatography in a 65:35 ratio (hexane/AcOEt). (308 mg, 80%), white solid. mp 172-175 °C.

HRMS (DART+) calculated for $C_{30}H_{32}ClN_2O_6$ [M + H]$^{+}$ 551.1948 ; found 551.1926. **^{1}H NMR** (400 MHz; $CDCl_3$) δ: 8.06 (dd, *J* = 8 Hz, 1H), 7.93 (d, *J* = 8 Hz, 2H), 7.24 (d, *J* = 8 Hz, 1H), 7.2-7.06 (m, 2H), 6.91 (t, *J* = 8 Hz, 2H), 6.82 (d, *J* = 8 Hz, 2H), 6.50 (d, *J* = 8 Hz, 2H), 5.46 (s, 1H), 5.26 (s,1H), 4.13 (d, *J* =12 Hz, 1H), 4.02 (d, *J* = 4 Hz, 1H), 3.97 (d, *J* =12 Hz, 6H), 1.26 (s, 9 H). **^{13}C NMR** (100 MHz, $CDCl_3$) δ: 168.3, 167.5, 166.7, 165.9, 144.4, 138.0, 136.1, 135.4, 133.6, 131.9, 131.4, 131.2, 130.8, 129.6, 128.9, 128.6, 128.2, 66.2, 52.6, 52.4, 51.8, 43.0, 28.6**. IR (ATR, νmax, cm^{-1}):** 2957, 1721, 1670, 1273, 1237, 1106, 760, 698.

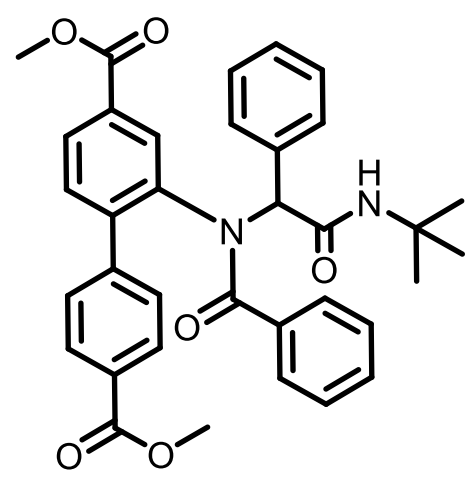

DBPDC-Ugi-3

Synthesis of dimethyl 2-(N-(2-(tert-butylamino)-2-oxo-1-phenylethyl)benzamido)-[1,1'-biphenyl]-4,4'-dicarboxylate (DBPDC-Ugi-3). Dimethyl 2-amino-[1,1'-biphenyl]-4,4'-dicarboxylate (DBPDC-NH_2) (200 mg, 0.701 mmol) was placed together with 10 mL of methanol and benzaldehyde (71.5 µL, 0.701 mmol), benzoic acid (85.6 mg, 7.0 mmol), and *tert*-butyl isocyanide (79.3 µL, 0.701 mmol) in a 50-mL round-bottom flask. The reaction was stirred for 48 h at room temperature. The product was purified by flash column chromatography in a 7:3 ratio (hexane/AcOEt). (210 mg, 52%), white solid. mp 174-178 °C.

HRMS (DART+) calculated for $C_{35}H_{35}N_2O_6$ $[M + H]^+$ 579.2465 ; found 579.2479. **^{1}H NMR** (400 MHz; $CDCl_3$) δ: 8.59 (d, *J* = 4 Hz, 1H), 8.11-8.05 (m, 1H), 8.01 (dd, *J* = 4 Hz, 1H), 7.91 (d, *J* = 8 Hz, 2H), 7.47-7.38 (m, 1H), 7.21-7.07 (m, 4H), 6.89 (t, *J* = 8 Hz, 3H), 6.83 (d, *J* = 8 Hz, 2H), 6.51 (d, *J* = 8 Hz, 2H), 5.45 (s, 1H), 5.29 (s, 1H), 3.96 (d, J=12 Hz, 6H), 1.25 (s, 9H). **^{13}C NMR** (100 MHz, $CDCl_3$) δ: 171.9, 169.0, 166.8, 166.2, 144.3, 142.4, 138.1, 133.9, 132.7, 131.5, 131.0, 130.7, 130.1, 129.6, 129.5, 129.4, 129.0, 128.8, 128.5, 128.3, 128.1, 65.6, 52.5, 52.4, 51.6, 28.7. **IR (ATR, vmax, cm^{-1}):** 3286, 2965, 1722, 1848, 1274, 1112, 759.

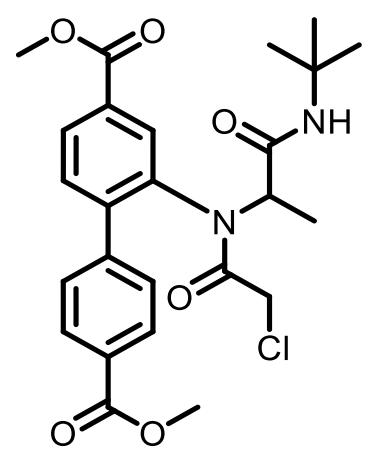

DBPDC-Ugi-4

Synthesis of dimethyl 2-(N-(1-(tert-butylamino)-1-oxopropan-2-yl)-2-chloroacetamido)-[1,1'-biphenyl]-4,4'-dicarboxylate (DBPDC-Ugi-4). Dimethyl 2-amino-[1,1'-biphenyl]-4,4'-dicarboxylate (DBPDC-NH_2) (200 mg, 0.701 mmol) was placed together with 10 mL of acetaldehyde (40 µL, 0.708 mmol), chloroacetic acid (66.2 mg, 7.0 mmol), and *tert*-butyl isocyanide (79.3 µL, 0.701 mmol) in a 50-mL round-bottom flask. The reaction was stirred for 96 h at room temperature. The product was purified by flash column chromatography in a 7:3 ratio (hexane/AcOEt). (298 mg, 87%), white solid. mp 173-176 °C.

HRMS (DART+) calculated for $C_{25}H_{30}Cl_1N_2O_6$ $[M + H]^+$ 489.1792 ; found 489.1782. **^{1}H NMR** (400 MHz; $CDCl_3$) δ: 8.20 (s, 2H), 8.12 (d, *J* = 12 Hz, 2H), 7.59 (d, *J* = 8 Hz, 1H), 7.37 (d, *J* = 8 Hz, 2H), 6.06 (s, 1H), 4.61 (q, *J* = 8 Hz, 1H), 3.95 (s, 6H), 3.83 (d, *J*= 16 Hz, 1H), 3.72 (d, *J* = 12 Hz, 1H), 1.37 (s, 9H), 1.29 (d, J = 8 Hz, 3H). **^{13}C NMR** (100 MHz, $CDCl_3$) δ: 170.1, 167.7, 166.5, 165.6, 144.0, 142.1, 135.7, 132.3, 131.8, 131.7, 131.0, 130.4, 129.0, 128.9, 57.2, 52.7, 52.5, 51.6, 42.4, 28.8, 14.6. **IR (ATR, vmax, cm^{-1}):** 3556, 2965, 1652, 1290, 1230, 1116, 759.

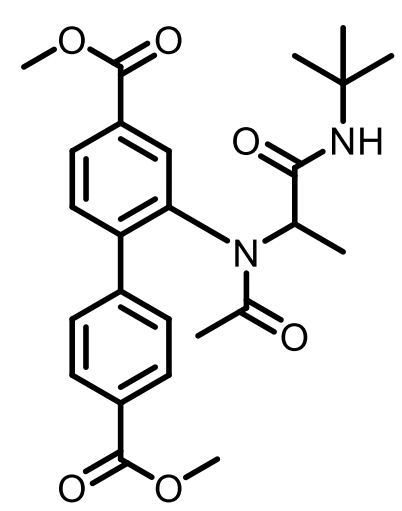

DBPDC-Ugi-5

Synthesis of dimethyl 2-(N-(1-(tert-butylamino)-1-oxopropan-2-yl)acetamido)-[1,1'-biphenyl]-4,4'-dicarboxylate (DBPDC-Ugi-5). Dimethyl 2-amino-[1,1'-biphenyl]-4,4'-dicarboxylate (DBPDC-NH_2) (200 mg, 0.701 mmol) was placed together with 10 mL of acetaldehyde (40 µL, 0.708 mmol), acetic acid (40.1 µL, 0.701 mmol), and *tert*-butyl isocyanide (79.3 µL, 0.701 mmol) in a 50-mL round-bottom flask. The reaction was stirred for 4 h at room temperature. The product was purified by flash column chromatography in a 7:3 ratio (hexane/AcOEt). (233 mg, 68%), white solid. mp 170-175 °C.

HRMS (DART+) calculated for $C_{25}H_{31}N_2O_6$ $[M + H]^+$ 455.1181; found 455.1189. **^{1}H NMR** (400 MHz; $CDCl_3$) δ: 8.12 (t, *J* = *8* Hz, 4H), 7.57 (d, *J* = 8 Hz, 1H), 7.41 (d, *J* = 8 Hz, 2H), 6.19 (s, 1H), 4.61 (q, *J* = 8 Hz, 1H), 3.94 (d, *J* = 4 Hz, 6H), 1.37 (s, 9H), 1.28 (s, 3H), 0.85 (d, *J* = 8 Hz, 3H). **^{13}C NMR** (100 MHz, $CDCl_3$) δ: ^{13}C NMR (76 MHz, CDCl3) δ 171.9, 170.9, 166.6, 165.9, 144.1, 142.8, 137.5, 132.3, 131.6, 131.4, 130.3, 130.2, 130.1, 128.8, 56.6, 52.6, 52.4, 51.3, 28.8, 23.8, 14.9. **IR (ATR, vmax, cm^{-1}):** 2967, 1722, 1663, 1283, 1235, 1111, 762.

2.5 General procedure for the synthesis of organic linkers

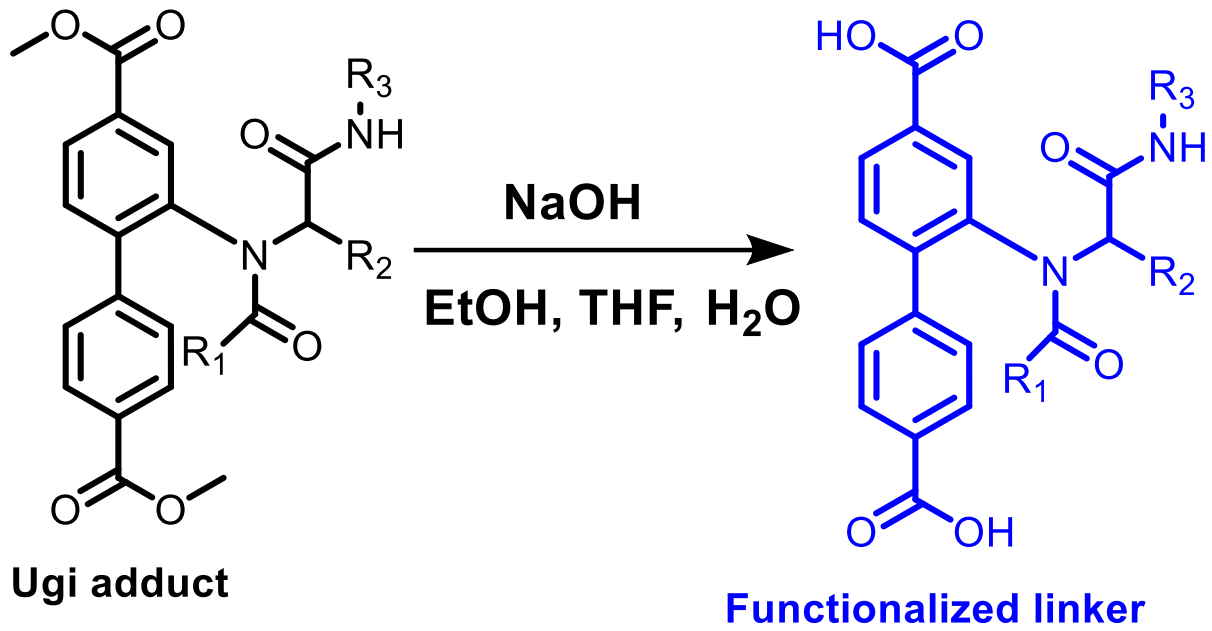

Scheme S2. General reaction of hydrolysis of DBPDC-Ugi adducts with NaOH.

The Ugi ester adduct was placed in a 50 mL round-bottom flask, and the appropriate amount of powdered NaOH was added. A solvent mixture of THF, ethanol, and Milli-Q water (2:1:1 v/v/v; 2 mL THF, 1 mL ethanol, and 1 mL water) was then added, and the flask was sealed with a Teflon stopper. The reaction mixture was stirred at room temperature for 12 h. After completion, the product was precipitated by dropwise addition of aqueous HCl until pH = 1 was reached. The resulting solid was collected by vacuum filtration and washed with water to afford the product as a white powder. This synthetic procedure was adapted from previously reported protocols for organic linkers formation.[11]

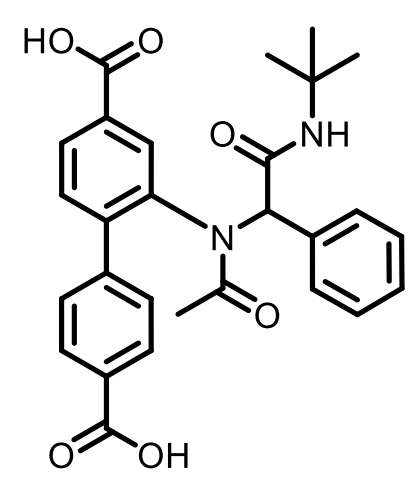

H_2BPDC-Ugi-1

Synthesis of 2-(N-(2-(tert-butylamino)-2-oxo-1-phenylethyl)acetamido)-[1,1'-biphenyl]-4,4'-dicarboxylic acid (H_2BPDC-Ugi-1). The DBPDC-Ugi-1 adduct (0.15 g, 0.307 mmol) was placed in a 50-mL round-bottom flask, and NaOH (245 mg, 6.1 mmol, 20 Eq) was added along with the described solvents. The reaction was stirred for 12 h, and then the compound was precipitated. The product was obtained as a colorless white powder (135 mg, 95%). mp 290-295 °C.

HRMS (DART+) calculated for $C_{28}H_{29}N_2O_6$ $[M + H]^+$ 489.2025; found 489.2012. **^{1}H NMR** (300 MHz; DMSO-d$_6$) δH: 8.52 (s, 1H), 7.92 (d, *J* = 9 Hz, 2H), 7.84 (d, *J* = 9 Hz, 1H), 7.48 (s, 1H), 7.28 (d, *J* = 9 Hz, 1H), 7.05-6.96 (m, 4H), 6.88 (t, *J* = 6 Hz, 2H), 6.52 (d, *J* = 6 Hz, 1H), 5.38 (s, 1H), 1.85 (s, 3H), 1.12 (s, 9H). **^{13}C NMR** (75 MHz, DMSO-d$_6$) δ: 170.6, 170.1, 167.7, 167.2, 143.9, 142.4, 138.7, 134.7, 133.9, 131.5, 131.2, 130.9, 130.5, 129.9, 129.6, 128.2, 127.9, 65.2, 50.6, 28.9, 24.4. **IR (ATR, vmax, cm^{-1}):** 2977, 1687, 1552, 1427, 1294, 927, 760.

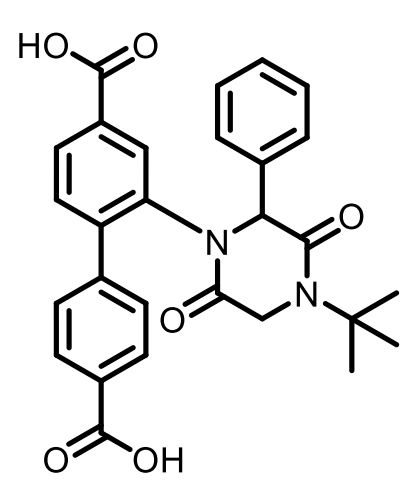

H_2BPDC-Ugi-2

Synthesis of 2-(4-(tert-butyl)-3,6-dioxo-2-phenylpiperazin-1-yl)-[1,1'-biphenyl]-4,4'-dicarboxylic acid (H_2BPDC-Ugi-2). The DBPDC-Ugi-2 adduct (0.15 g, 0.308 mmol) was placed in a 50-mL round-bottom flask, and NaOH (247 mg, 6.2 mmol, 20 Eq) was added along with the described solvents. The reaction was stirred for 24 h, and then the compound was precipitated. The product was obtained as a colorless white powder (66.2 mg, 50%). mp 303-310 °C.

HRMS (DART+) calculated for $C_{28}H_{27}N_2O_6$ $[M + H]^+$ 487.1869; found 487.1856. **^{1}H NMR** (300 MHz; DMSO-d$_6$) δH: 8.06 (d, *J* = 6 Hz, 1H), 7.97 (d, *J* = 9 Hz, 1H), 7.62 (d, *J* = 9 Hz, 1H), 7.53 (d, *J* = 6 Hz, 2H), 7.45-7.23 (m, 7H), 4.48-4.37 (m, 2H), 4.15 (d, *J* = 18 Hz, 1H), 1.25 (s, 9H). **^{13}C NMR** (75 MHz, DMSO-d$_6$) δ: 167, 166.1, 165.3, 164.1, 142.5, 141.4, 136.4, 133.5, 131.1, 130.6, 129.8, 129.4, 128.9, 128.5, 128.3, 127.0, 67.6, 57.4, 46.6, 40.4, 27.0. **IR (ATR, vmax, cm^{-1}):** 2973, 1672, 1427, 1286, 1224, 1130, 760 cm.

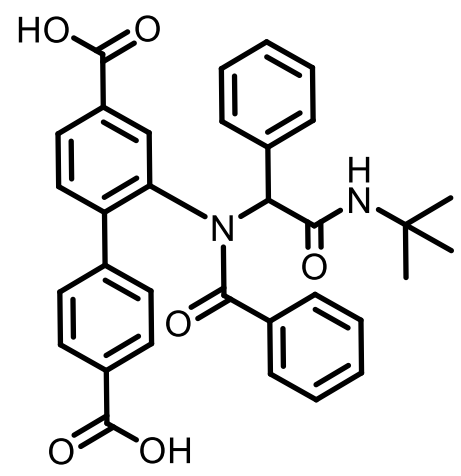

H_2BPDC-Ugi-3

Synthesis of 2-(N-(2-(tert-butylamino)-2-oxo-1-phenylethyl)benzamido)-[1,1'-biphenyl]-4,4'-dicarboxylic acid (H_2BPDC-Ugi-3). The DBPDC-Ugi-3 adduct (0.15 g, 0.272 mmol) was placed in a 50-mL round-bottom flask, and NaOH (218 mg, 5.4 mmol, 20 Eq) was added along with the described solvents. The reaction was stirred for 12 h, and then the compound was precipitated. The product was obtained as a colorless white powder (103 mg, 72%). mp 299-305 °C.

HRMS (DART+) calculated for $C_{33}H_{31}N_2O_6$ $[M + H]^+$ 551.1163; found 551.1170. ^{1}**H NMR** (400 MHz; DMSO-d_6) δH: 8.48 (s, 1H), 7.88 (d, *J* = 8 Hz, 4H), 7.81 (d, *J* = 4 Hz, 1H), 7.41 (s, 1H), 7.24 (d, *J* = 8 Hz, 1H), 7.01 (t, *J* = 8 Hz, 2H), 6.95 (d, *J* = 8 Hz, 3H), 6.84 (t, *J* = 8, 3H), 6.48 (d, *J* = 8 Hz, 2H), 5.35 (s, 1H), 4.31 (s, 1H), 1.09 (s, 9H). ^{13}**C NMR** (100 MHz, DMSO) δ 169.7, 167.5, 166.9, 162.9, 156.4, 148.4, 144.4, 142.3, 137.9, 136.5, 134.3, 134.1, 131.3, 130.6, 130.3, 129.9, 129.6, 129.2, 128.9, 128.5, 128.2, 127.7, 66.8, 50.8, 28.8. **IR (ATR, νmax, cm^{-1}):** 2952, 1716, 1633, 1432, 1274, 1222, 1112 cm.

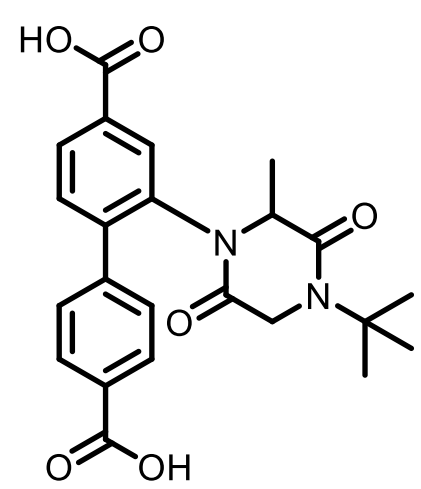

H_2BPDC-Ugi-4

Synthesis of 2-(4-(tert-butyl)-2-methyl-3,6-dioxopiperazin-1-yl)-[1,1'-biphenyl]-4,4'-dicarboxylic acid (H_2BPDC-Ugi-4). The DBPDC-Ugi-4 adduct (0.15 g, 0.353 mmol) was placed in a 50-mL round-bottom flask, and NaOH (283 mg, 7.07 mmol, 20 Eq) was added along with the described solvents. The reaction was stirred for 24 h, and then the compound was precipitated. The product was obtained as a colorless white powder (98 mg, 75%). mp 301-307 °C.

HRMS (DART+) calculated for $C_{23}H_{25}N_2O_6$ $[M + H]^+$ 425.1131; found 425.1136. ^{1}**H NMR** (400 MHz; DMSO-d_6) δH: 8.03-7.99 (m, 1H), 7.94 (d, *J*= 8 Hz, 2H), 7.81 (s, 1H), 7.6 (d, *J* = 8 Hz, 1H), 7.38 (d, *J* = 8 Hz, 2H), 4.19 (d, *J* = 16 Hz, 1H), 3.99 (d, *J* = 16 Hz, 1H), 3.56 (q, *J* = 4 Hz, 1H), 1.25 (s, 9H), 1.18 (d, *J* = 8 Hz, 3H).^{13}**C NMR** (100 MHz, DMSO-d_6) δ: 172.2, 167.5, 167.1, 166.4, 143.6, 142.8, 136.3, 131.4, 130.3, 130.1, 129.7, 129.4, 129, 128.2, 67, 56.9, 50.1, 27.3, 25.1. **IR (ATR, νmax, cm^{-1}):** 2967, 1673, 1438, 1284, 1222, 1139, 850, 761 cm.

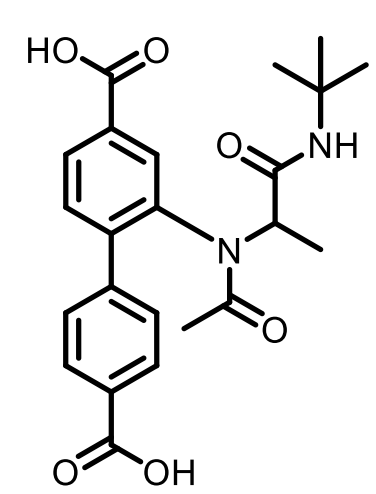

H_2BPDC-Ugi-5

Synthesis of 2-(N-(1-(tert-butylamino)-1-oxopropan-2-yl)acetamido)-[1,1'-biphenyl]-4,4'-dicarboxylic acid (H_2BPDC-Ugi-5). The DBPDC-Ugi-5 adduct (0.15 g, 0.351 mmol) was placed in a 50-mL round-bottom flask, and NaOH (281 mg, 7 mmol, 20 Eq) was added along with the described solvents. The reaction was stirred for 12 h, and then the compound was precipitated. The product was obtained as a colorless white powder (123 mg, 88%). mp 294-301 °C.

HRMS (DART+) calculated for $C_{23}H_{26}N_2O_6$ $[M + H]^+$ 427.1869; found 427.1861. ^{1}**H NMR** (400 MHz; DMSO-d_6) δH: 8.39 (s, 1H), 8.03 (d, *J* = 6 Hz, 2H), 7.66 (d, *J* = 6 Hz, 1H), 7.53 (s, 1H), 7.46 (d, *J* = 9 Hz, 2H), 4.46 (dd, *J* = 8, 6 Hz, 1H), 1.54 (s, 3H), 1.23 (s, 9H), 0.83 (d, *J* = 9 Hz, 3H). ^{13}**C NMR** (100 MHz, DMSO) δ 172.1, 169.6, 167.4, 167.0, 144.2, 142.8, 138.1, 132.9, 131.9, 131.6, 130.8, 130.0, 129.3, 128.9, 56.4, 50.5, 29.0, 23.6, 17.3. **IR (ATR, νmax, cm^{-1}):** 2969, 1725, 1620, 1392, 1181, 767, 699 cm.

3. Ugi adducts and organic linkers characterizations

3.1 DBPDC Ugi adducts ^1H and ^{13}C NMR spectra

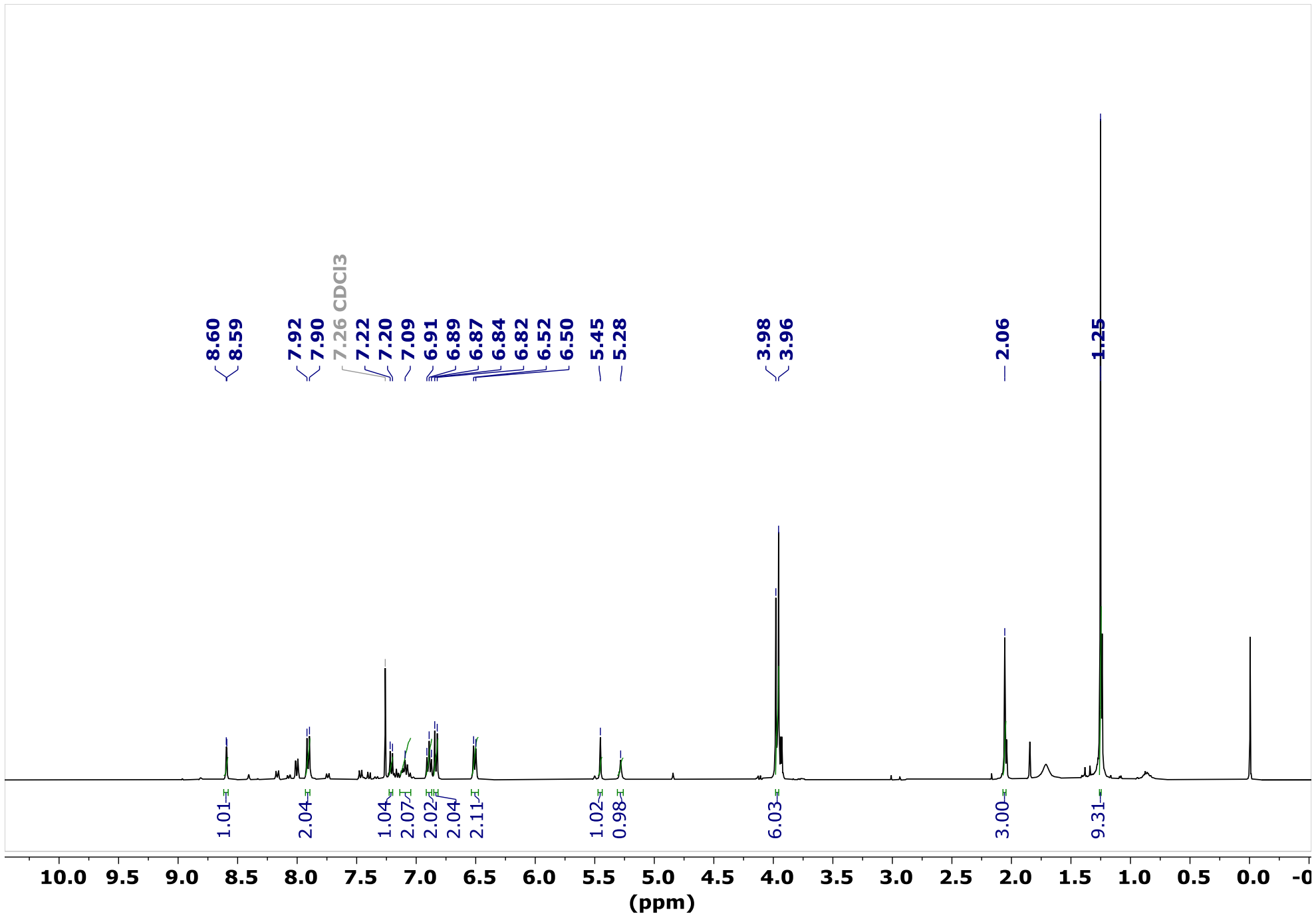

Figure S1. ^1H NMR spectra of DBPDC-Ugi-1 ($CDCl_3$, 400 MHz).

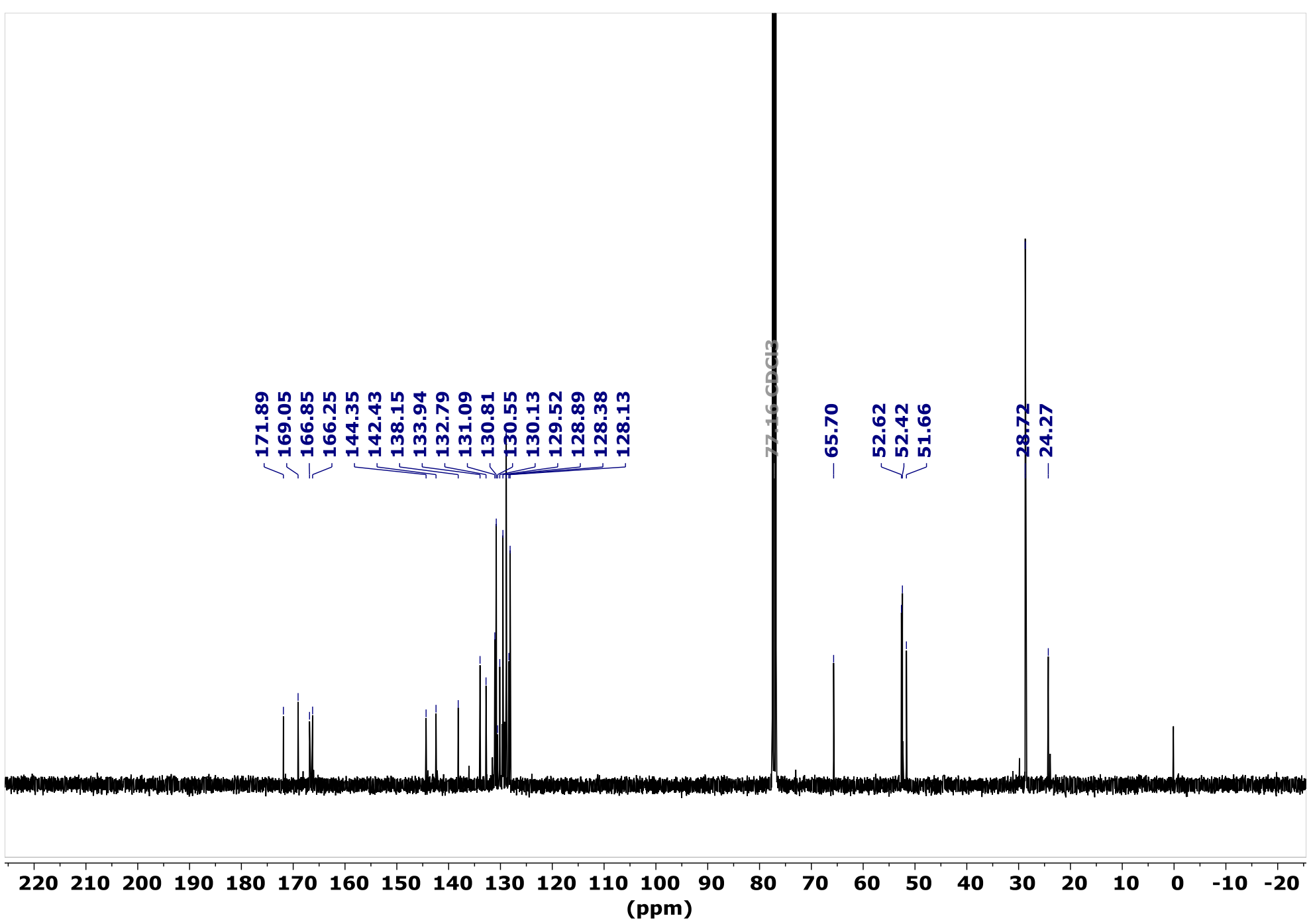

Figure S2. ^{13}C NMR spectra of DBPDC-Ugi-1 ($CDCl_3$, 100 MHz).

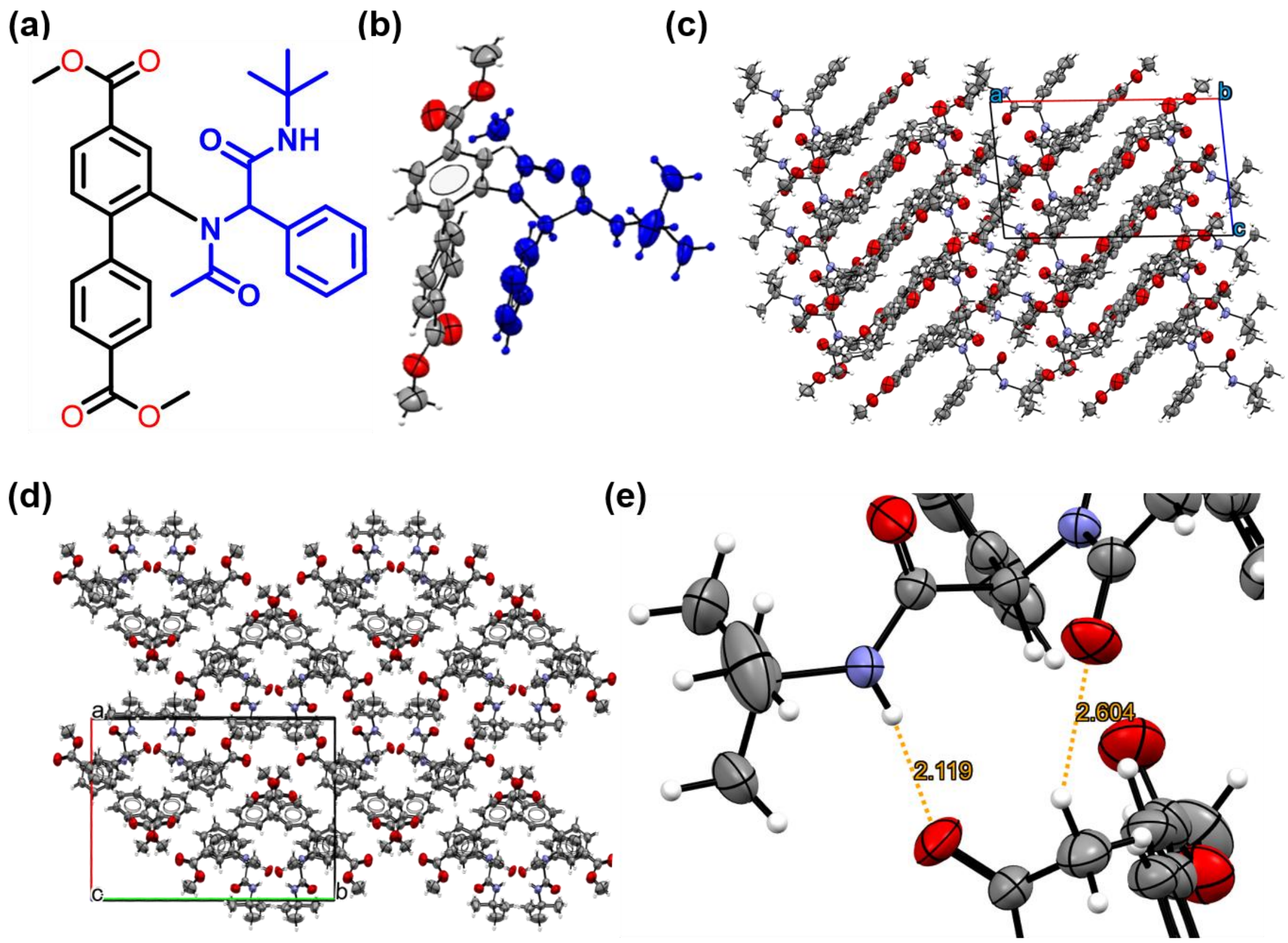

Figure S3. a) Ester of H_2BPDC-Ugi-1, **b)** Visualization of the ester in the crystal. **c)** Packing of the ester H_2BPDC-Ugi-1 as seen by b, **d)** as seen by c, **e)** Hydrogen bonds present in the structure N-H•••O (d = 2.119 Å) and C-H•••O (d = 2.604 Å).

Table S2. Crystal data and structure refinement for JT_UGI_1_4C_0ma_a.

Parameter	Value
Empirical formula	$C_{15}H_{20}N_4O_4$
Formula weight	320.35
Temperature (K)	294(2)
Wavelength (Å)	0.71073 (Mo Kα)
Crystal system	Monoclinic
Space group	$P2_1/c$
a (Å)	15.1572(10)
b (Å)	20.2935(15)
c (Å)	9.0863(6)
α (°)	90
β (°)	95.262(2)
γ (°)	90
Volume (Å³)	2783.1(3)
Z	8
Z′	2
Density (calculated) (g/cm³)	1.529
Absorption coefficient (mm⁻¹)	0.113
F(000)	1360
Crystal size (mm³)	0.120 × 0.080 × 0.050
Crystal description	Not reported
Crystal colour	Not reported
θ range for data collection (°)	1.681 to 26.602
Index ranges	$-18 \le h \le 19$, $-25 \le k \le 25$, $-11 \le l \le 11$
Reflections collected	48042
Independent reflections	5760 [R(int) = 0.3059]
Completeness to θ = 25.242°	99.9%
Refinement method	Full-matrix least-squares on F^2
Data / restraints / parameters	5760 / 0 / 350
Goodness-of-fit on F^2	1.034
Final R indices [I > 2σ(I)]	R_1 = 0.0783, wR_2 = 0.1856
R indices (all data)	R_1 = 0.1269, wR_2 = 0.2207
Extinction coefficient	0.025(3)
Largest diff. peak and hole (e Å⁻³)	0.284 and −0.293

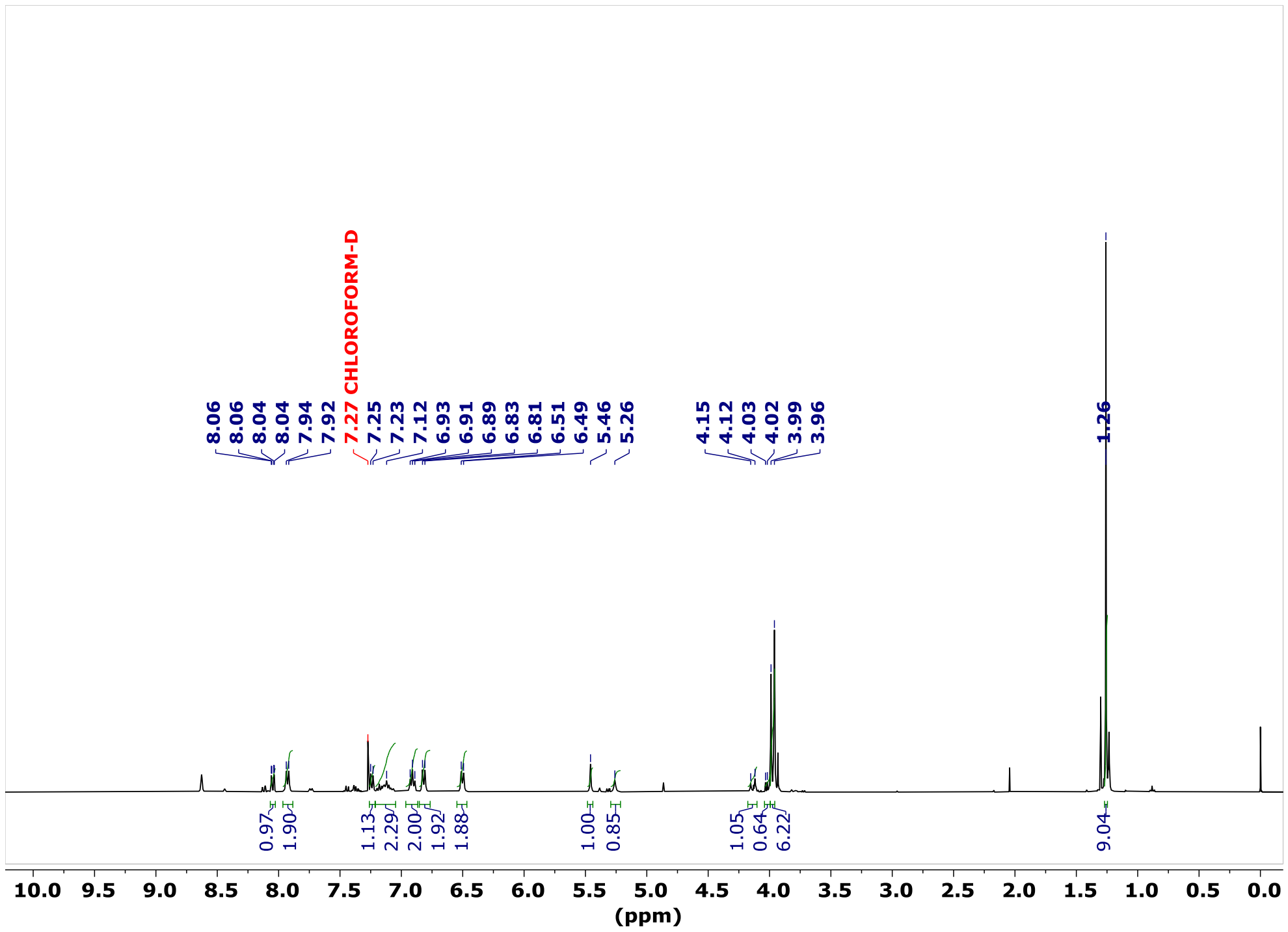

Figure S4. ^{1}H NMR spectra of DBPDC-Ugi-2 ($CDCl_3$, 400 MHz).

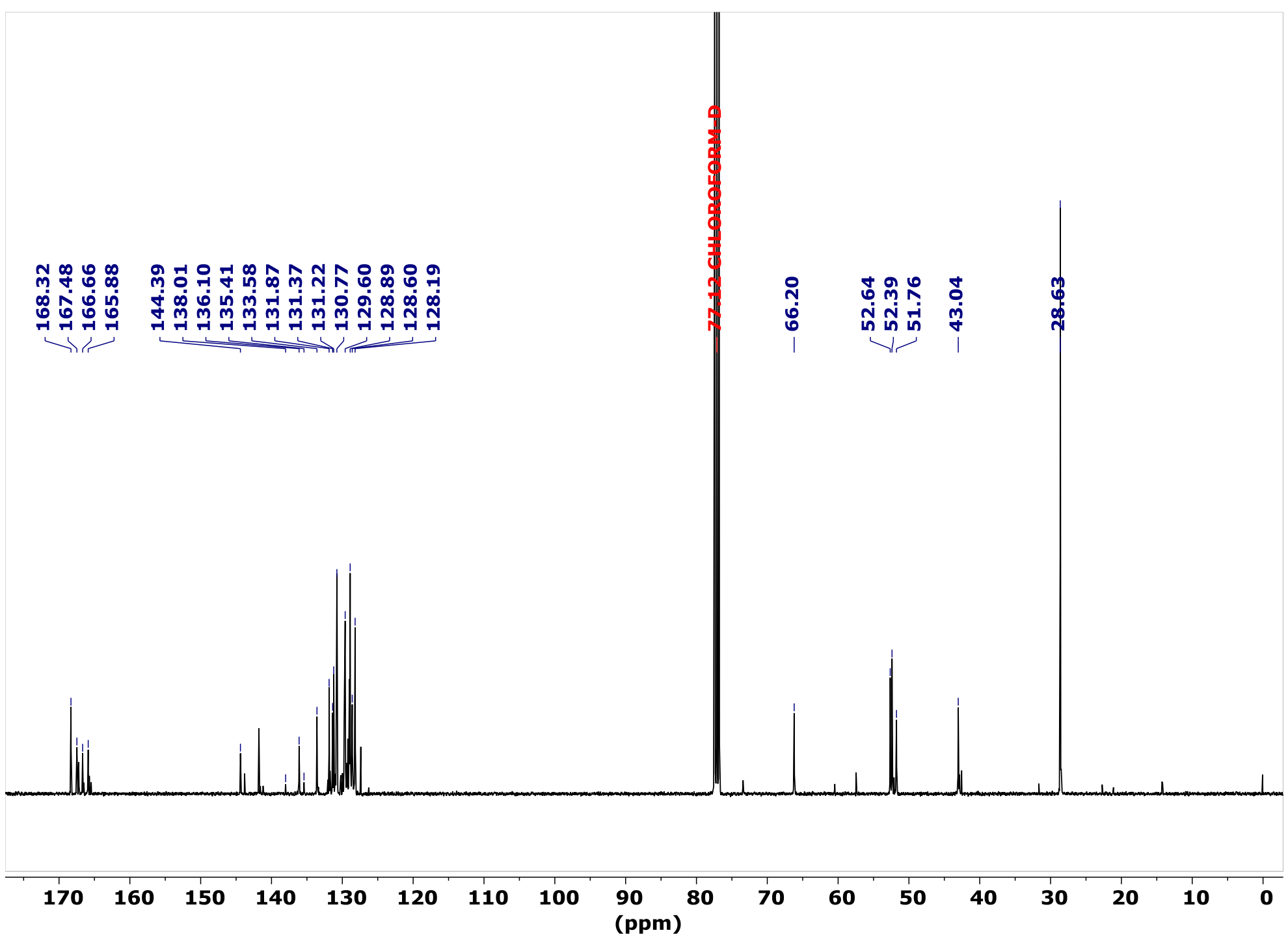

Figure S5. ^{13}C NMR spectra of DBPDC-Ugi-2 ($CDCl_3$, 100 MHz).

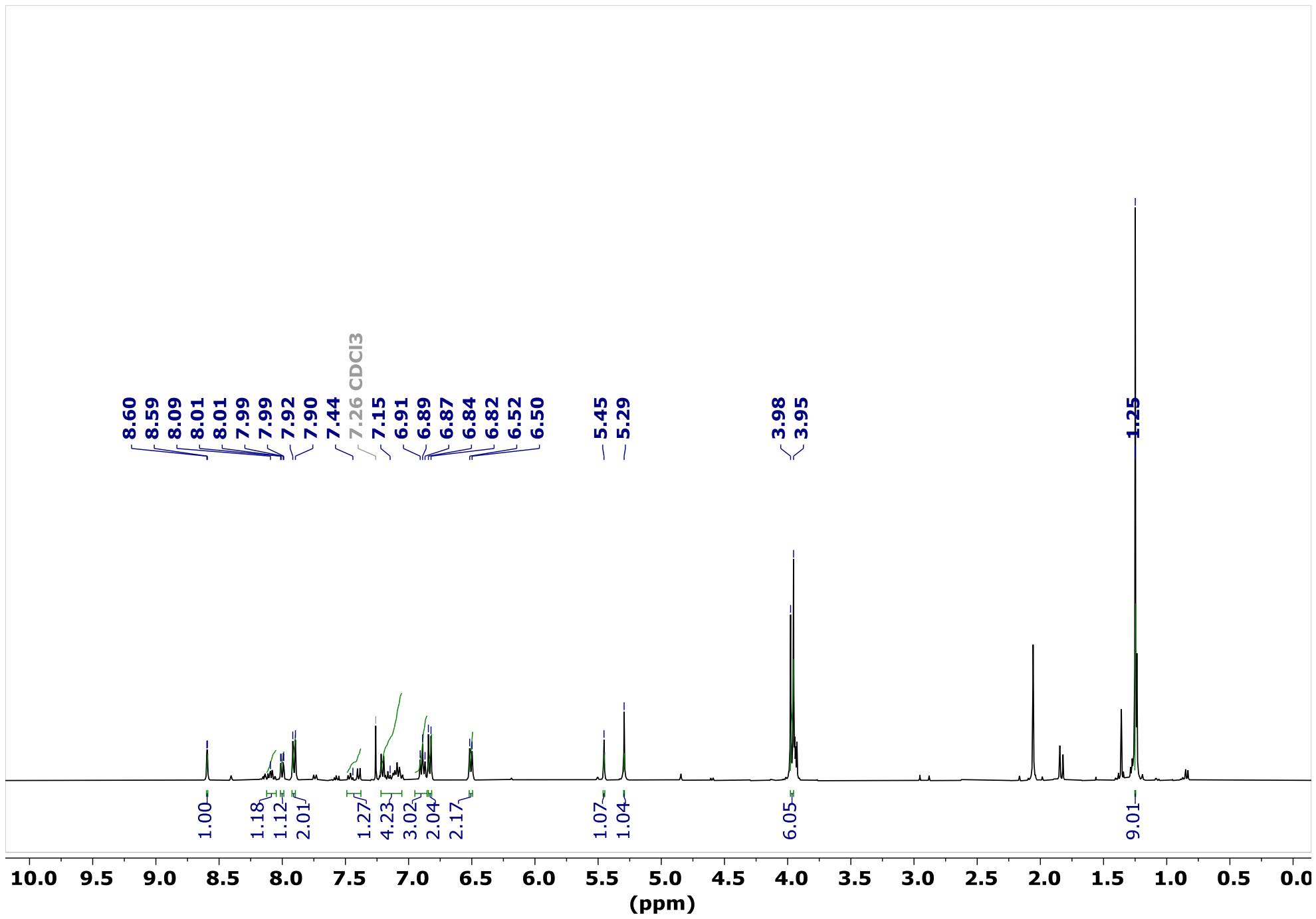

Figure S6. ^{1}H NMR spectra of DBPDC-Ugi-3 ($CDCl_3$, 400 MHz).

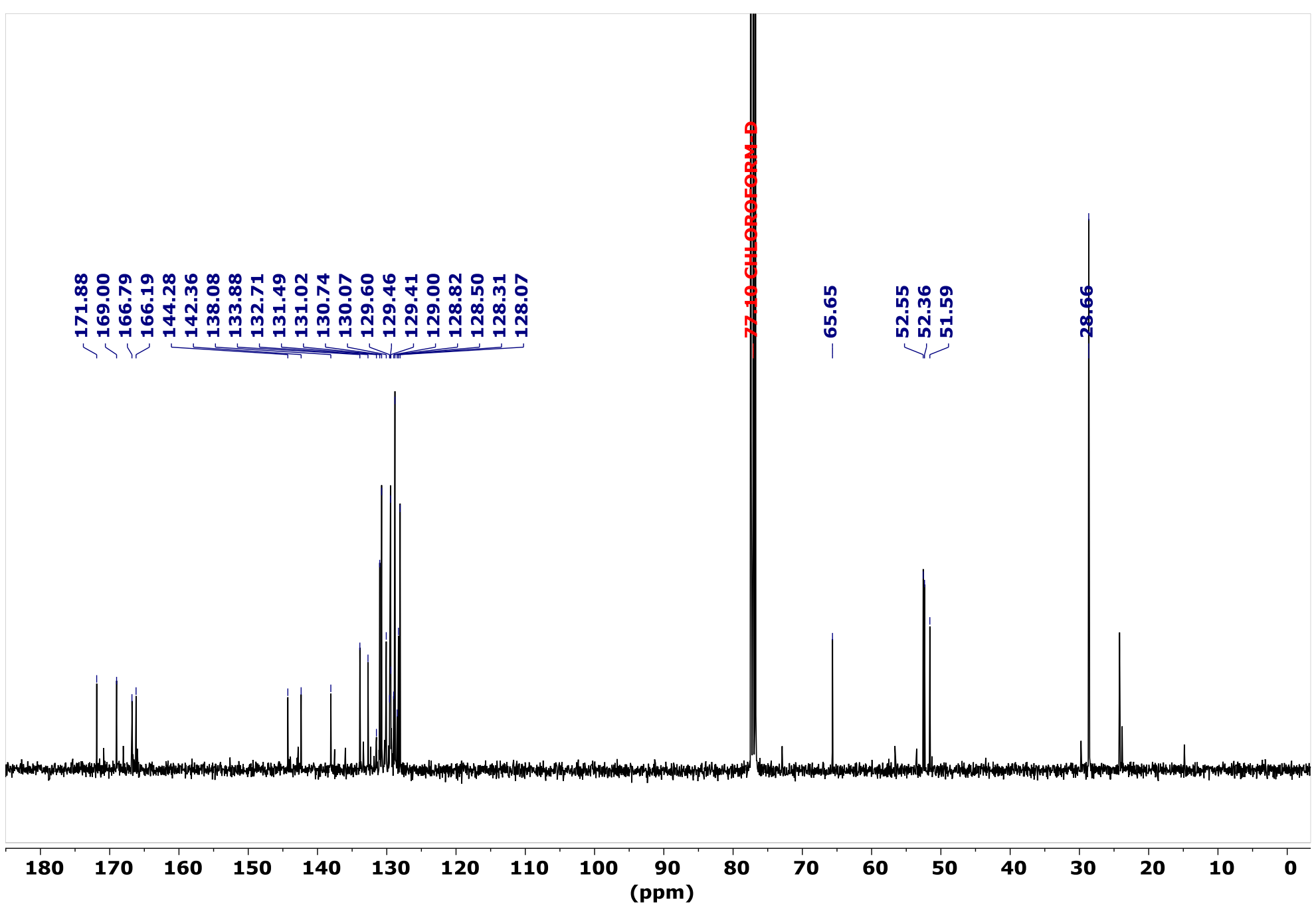

Figure S7. ^{13}C NMR spectra of DBPDC-Ugi-3 ($CDCl_3$, 100 MHz).

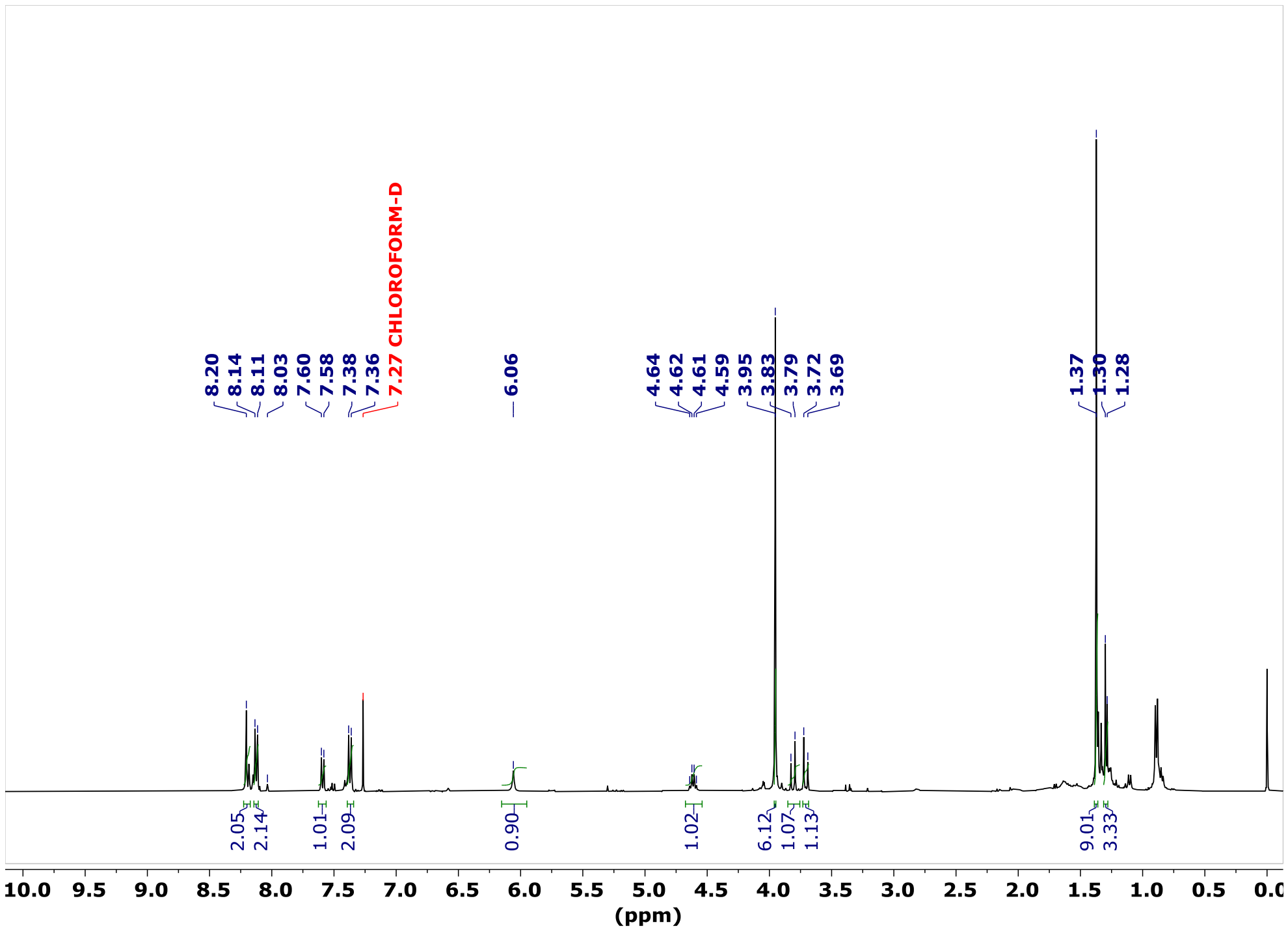

Figure S8. ^{1}H NMR spectra of DBPDC-Ugi-4 ($CDCl_3$, 400 MHz).

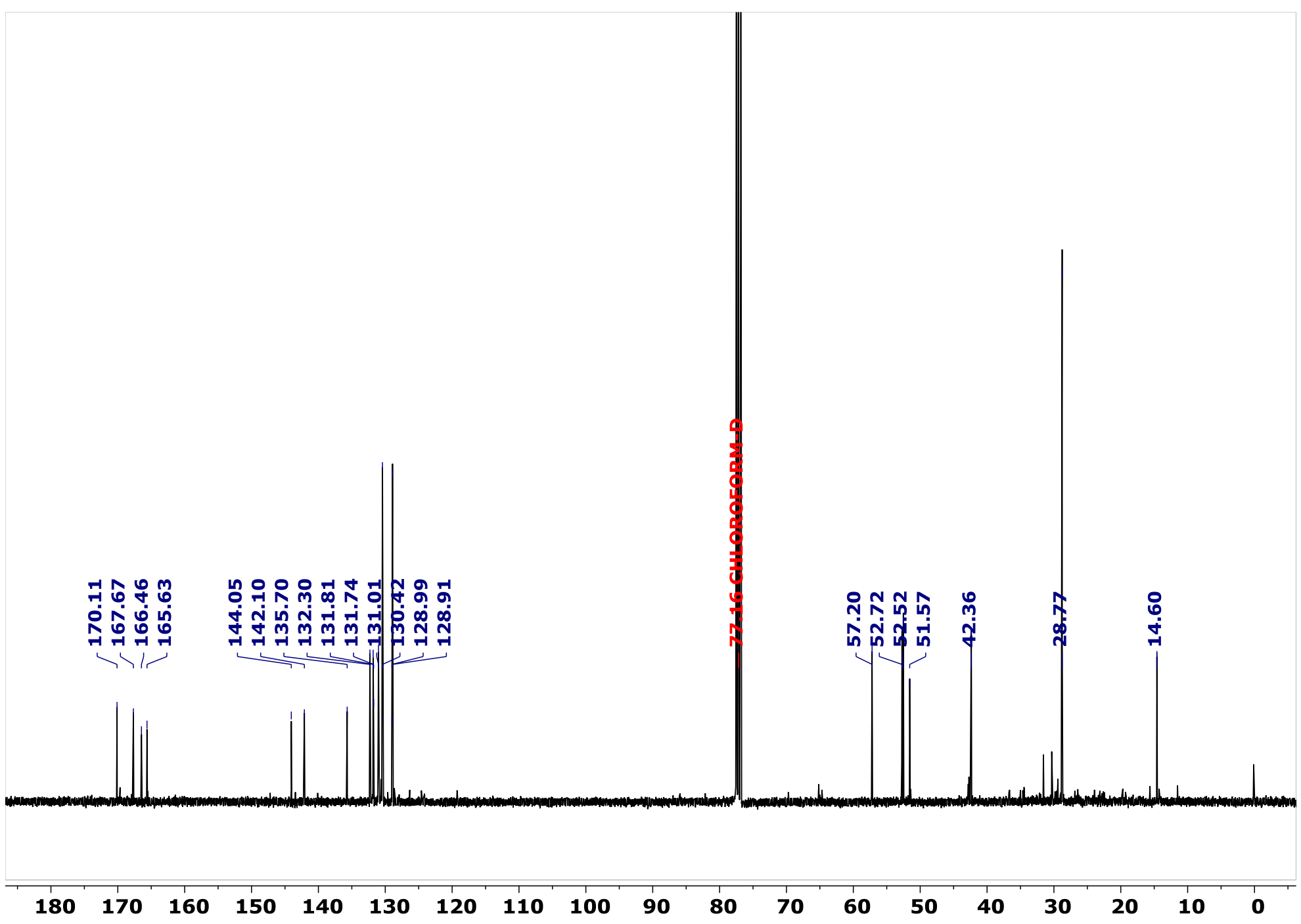

Figure S9. ^{13}C NMR spectra of DBPDC-Ugi-4 ($CDCl_3$, 100 MHz).

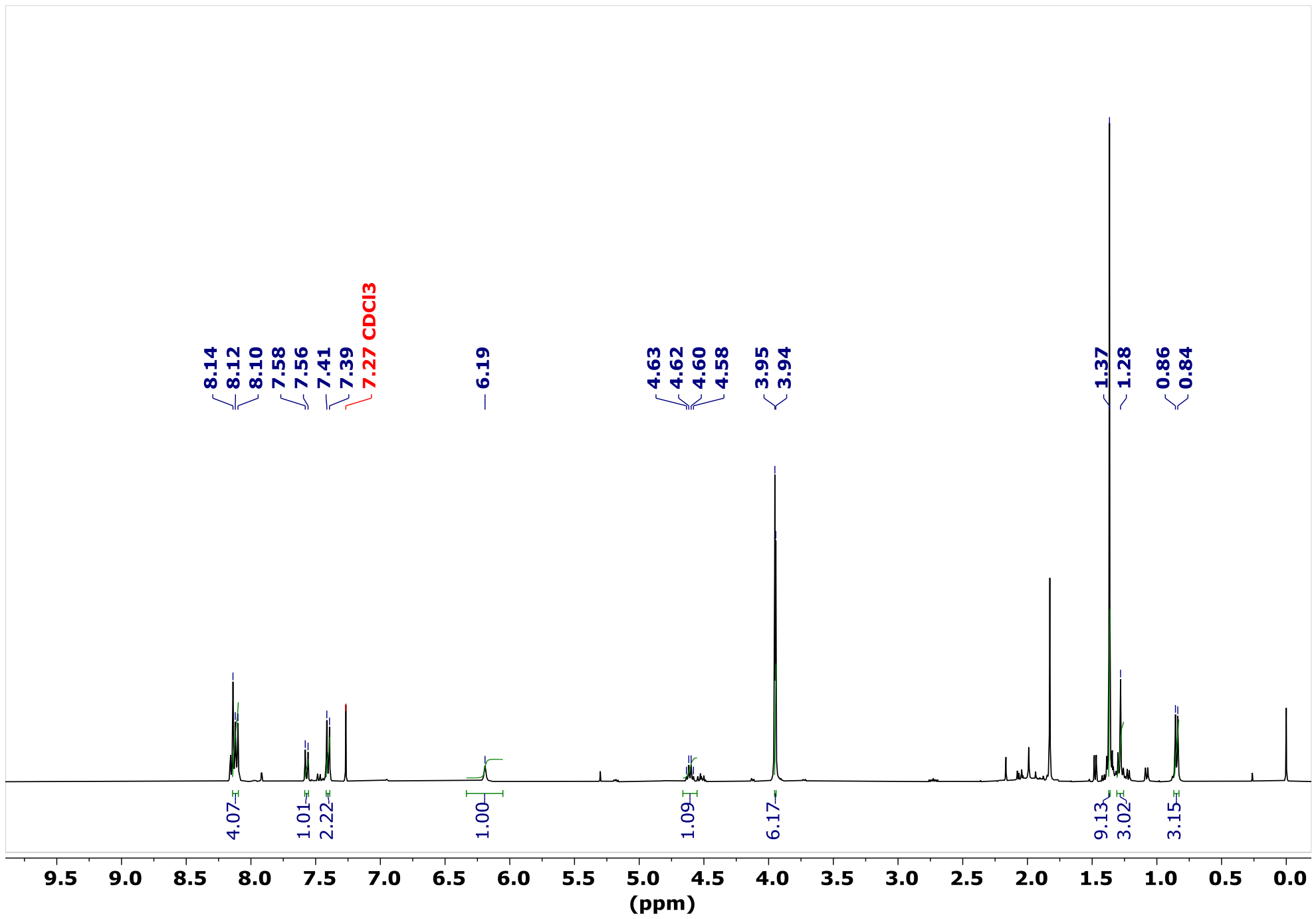

Figure S10. ^{1}H NMR spectra of DBPDC-Ugi-5 ($CDCl_3$, 400 MHz).

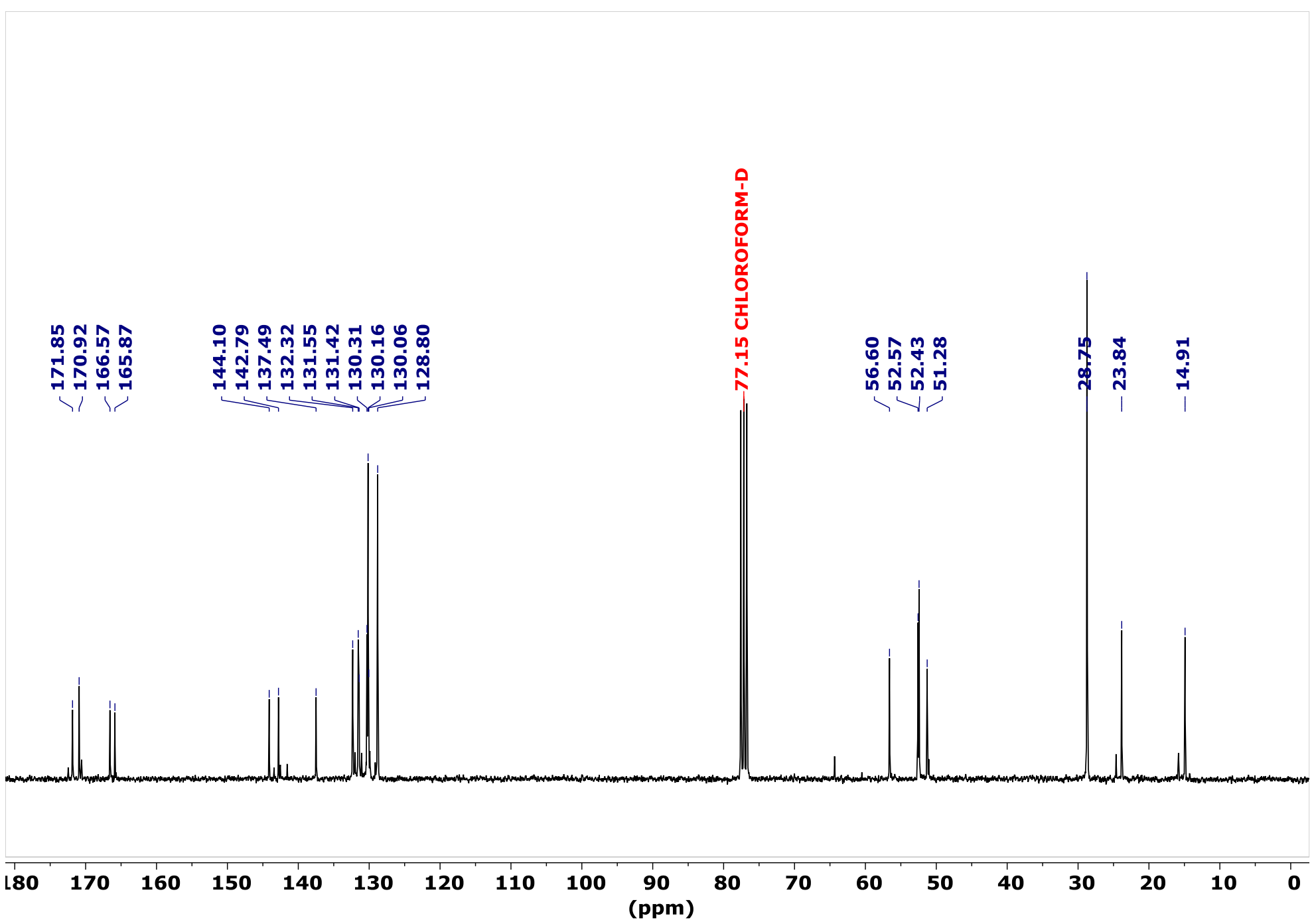

Figure S11. ^{13}C NMR spectra of DBPDC-Ugi-5 ($CDCl_3$, 100 MHz).

3.2 Ugi functionalized linkers ^{1}H and ^{13}C NMR spectra

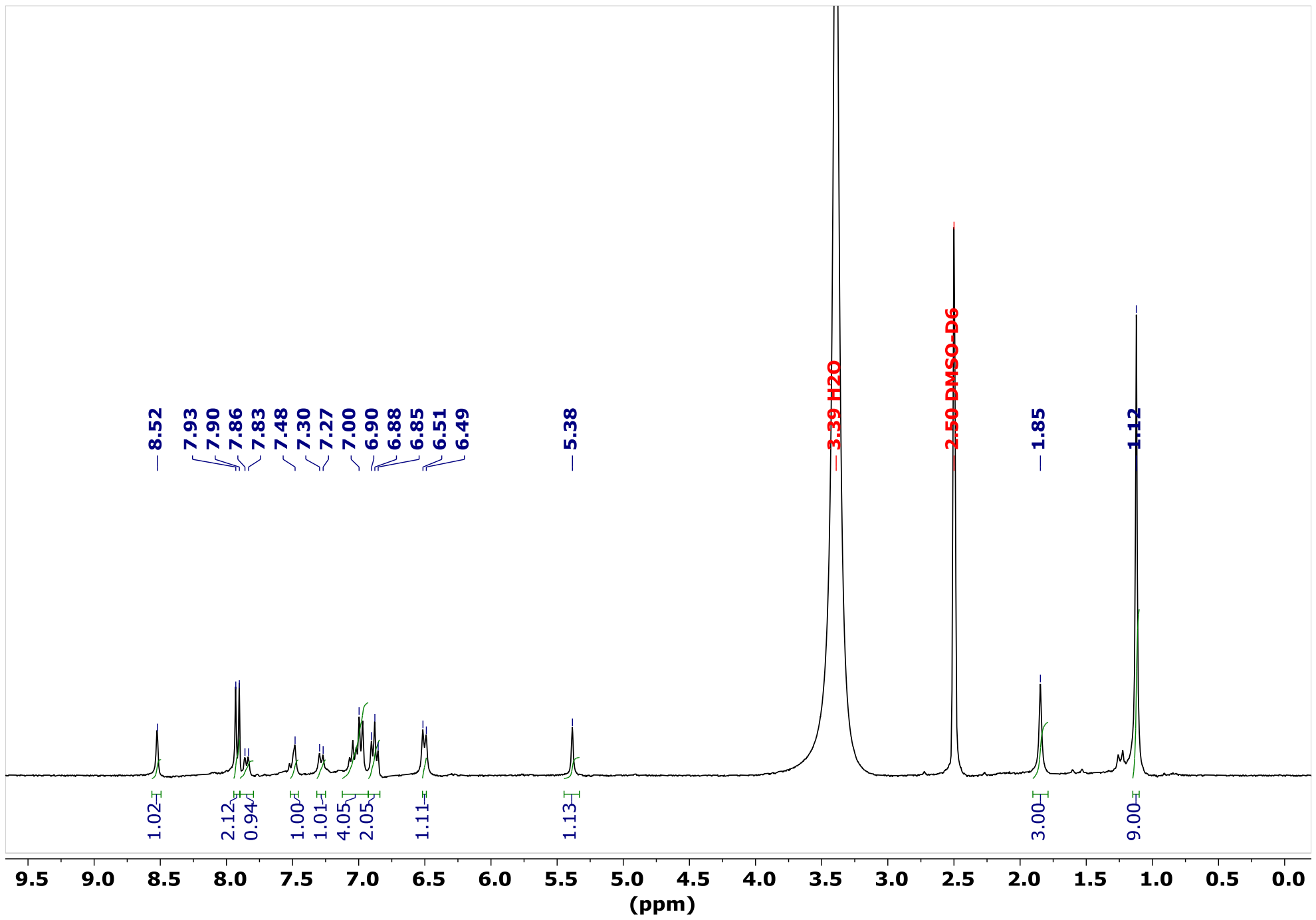

Figure S12. ^{1}H NMR spectra of H_2BPDC-Ugi-1 (DMSO-d_6, 300 MHz)

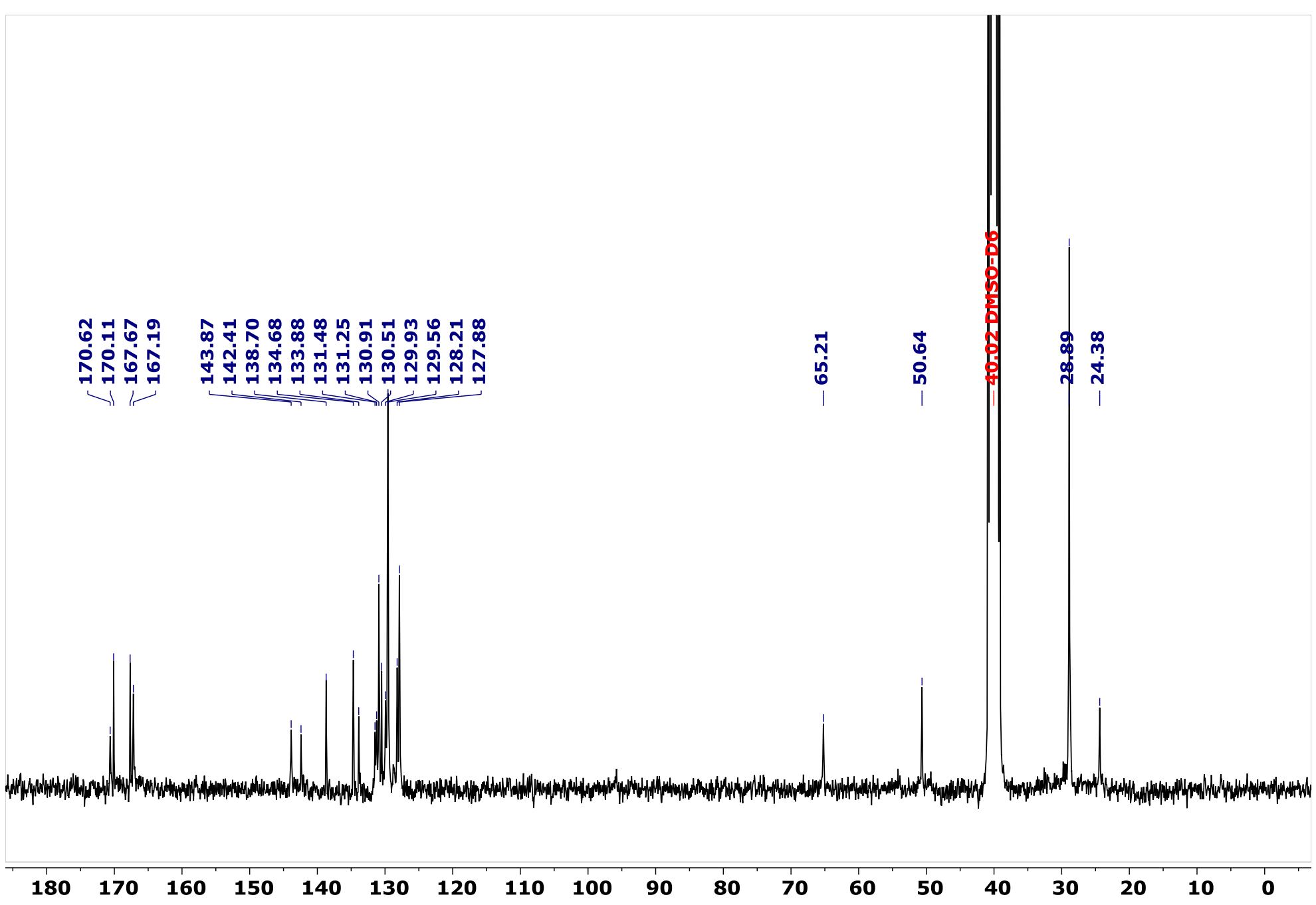

Figure S13. ^{13}C NMR spectra of H_2BPDC-Ugi-1 (DMSO-d_6, 75 MHz)

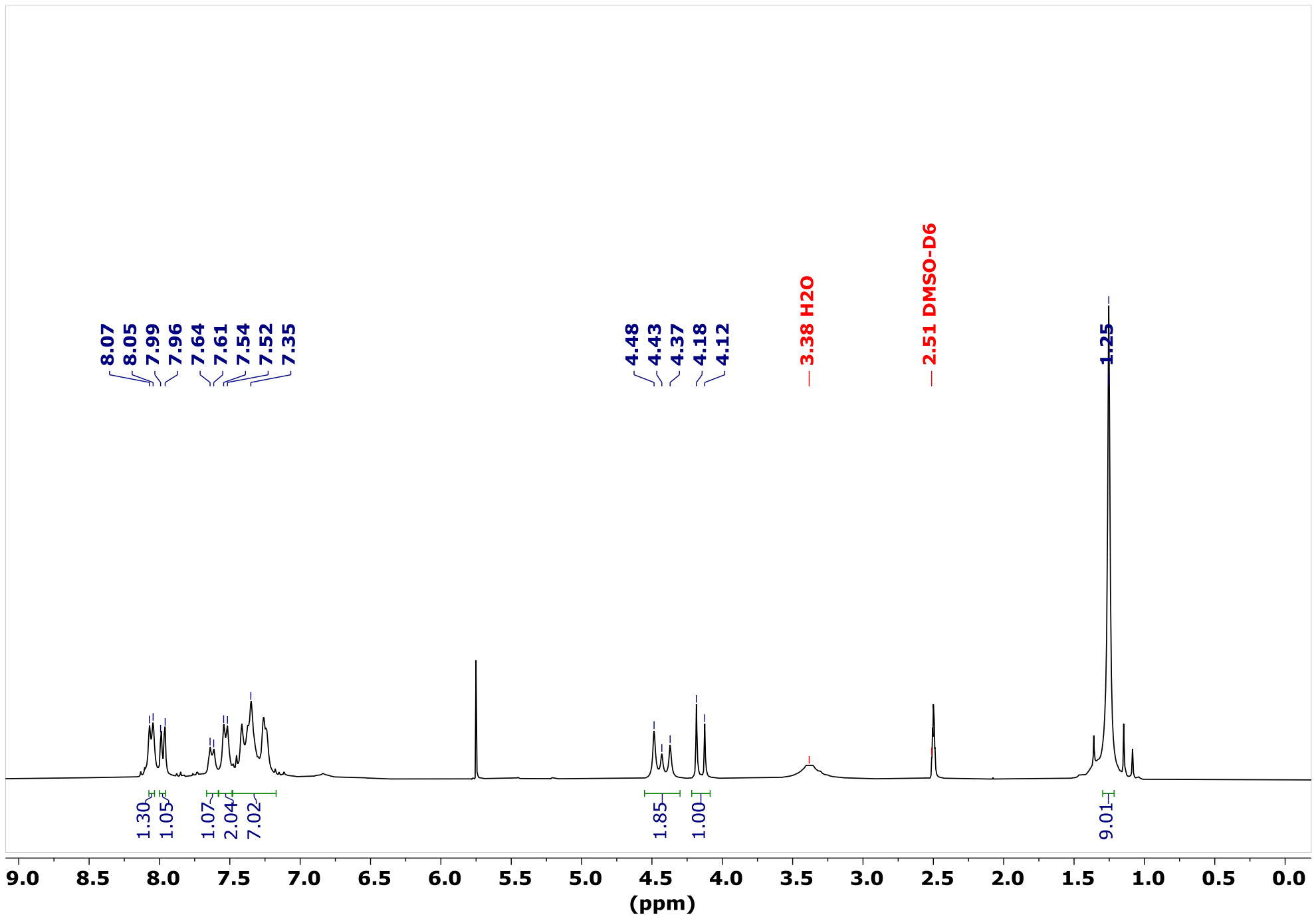

Figure S14. ^{1}H NMR spectra of H_2BPDC-Ugi-2 (DMSO-d_6, 300 MHz)

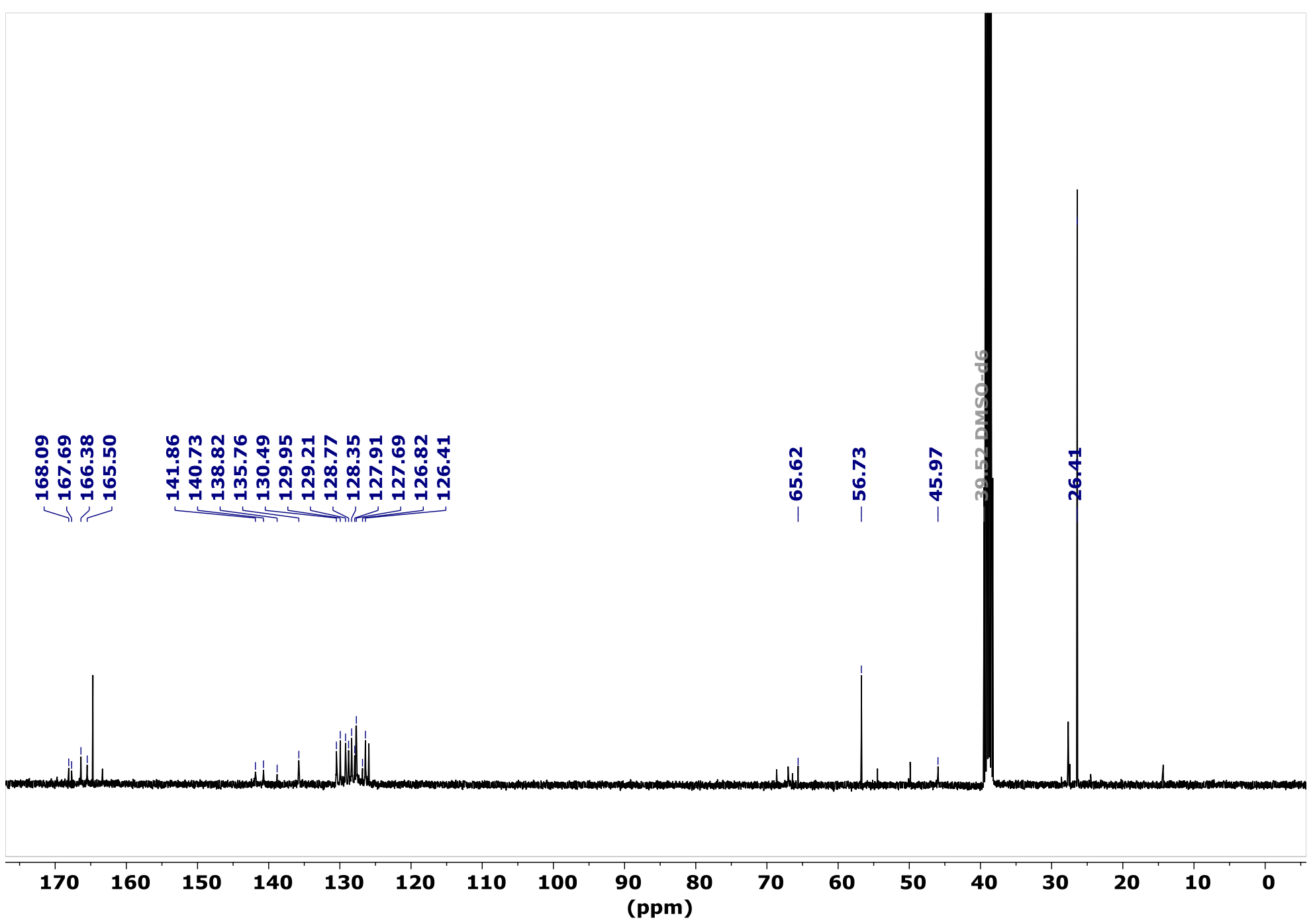

Figure S15. ^{13}C NMR spectra of H_2BPDC-Ugi-2 (DMSO-d_6, 75 MHz)

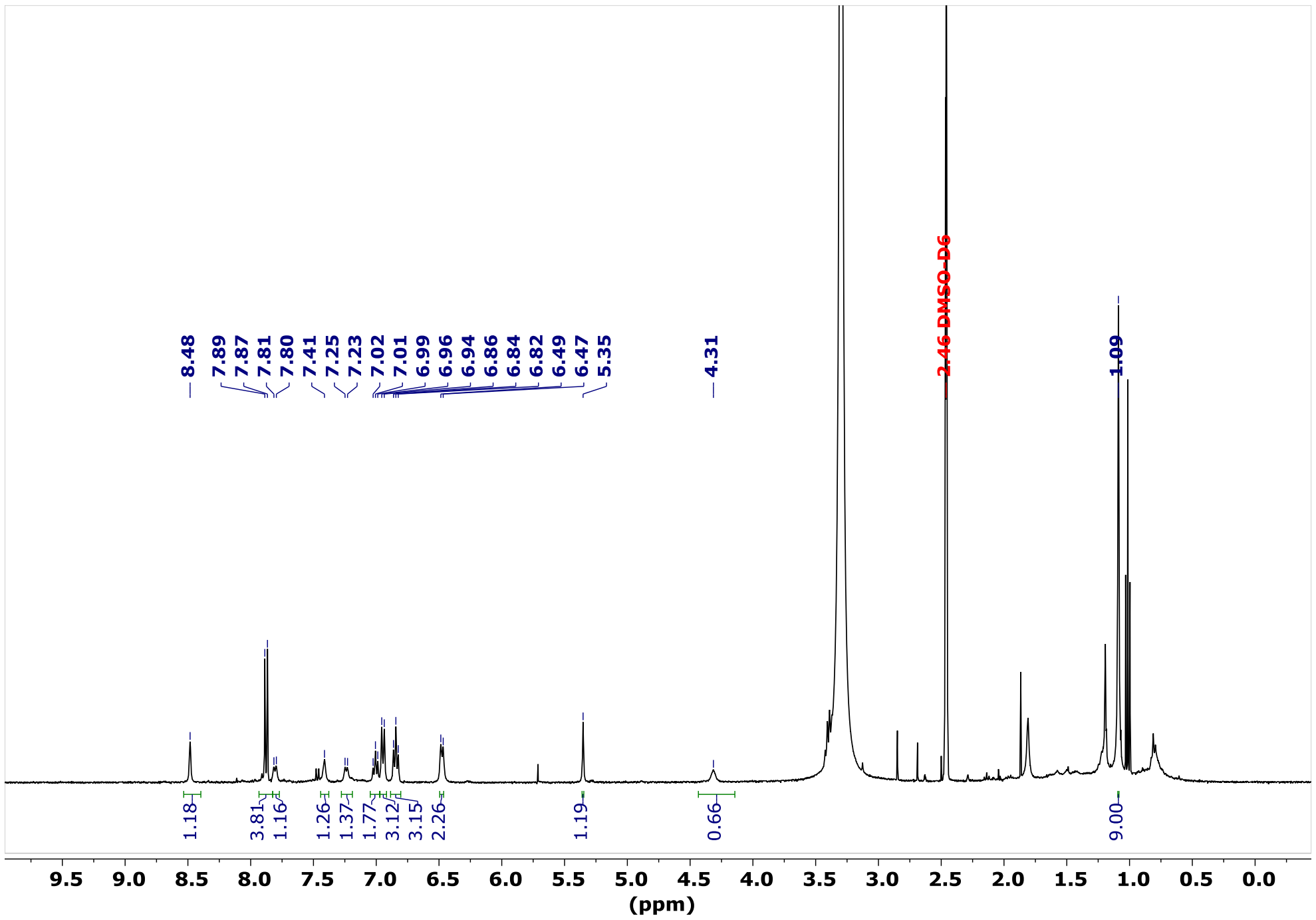

Figure S16. ^{1}H NMR spectra of H_2BPDC-Ugi-3 (DMSO-d_6, 400 MHz,).

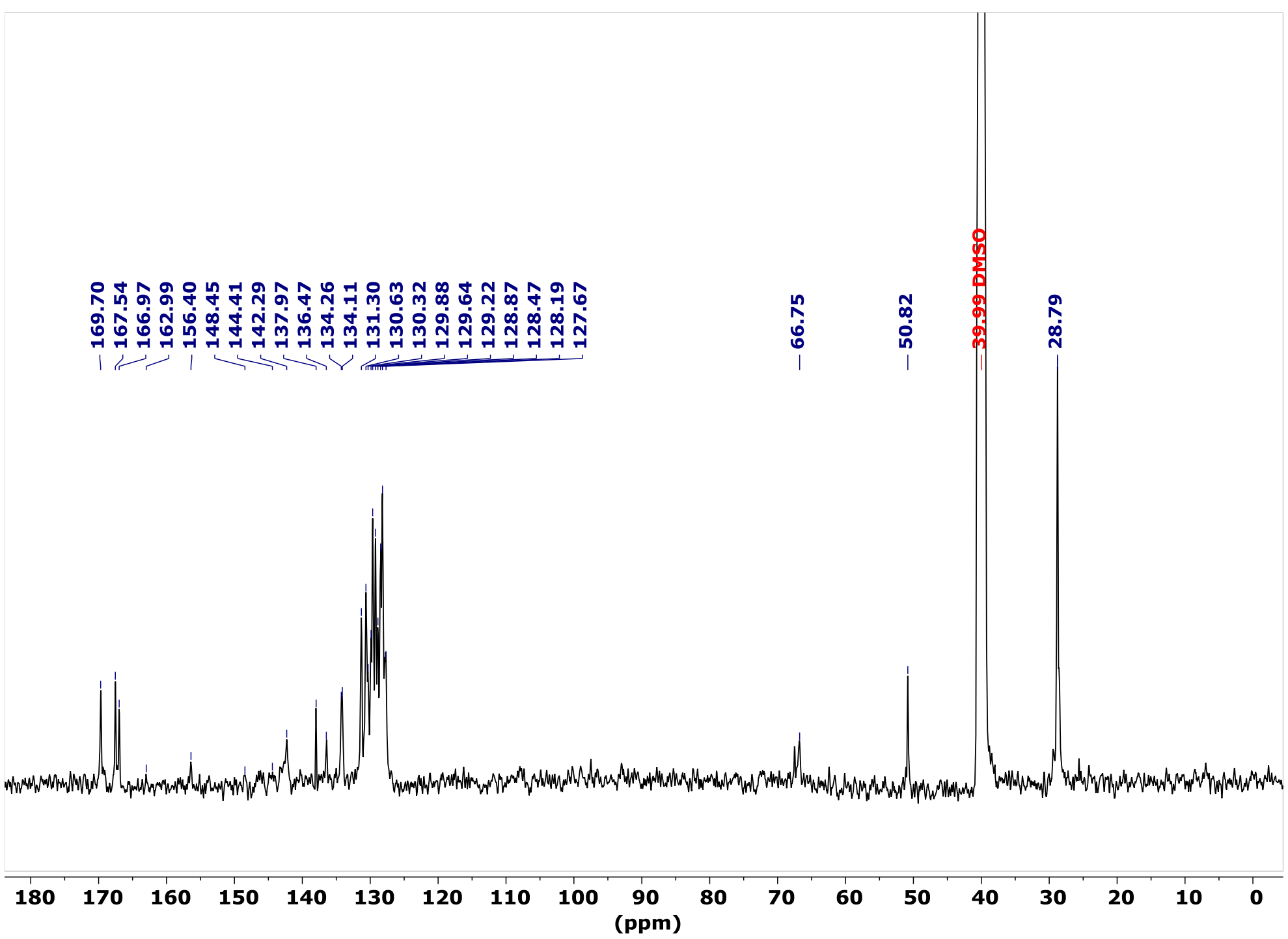

Figure S17. ^{13}C NMR spectra of H_2BPDC-Ugi-3 (DMSO-d_6, 100 MHz).

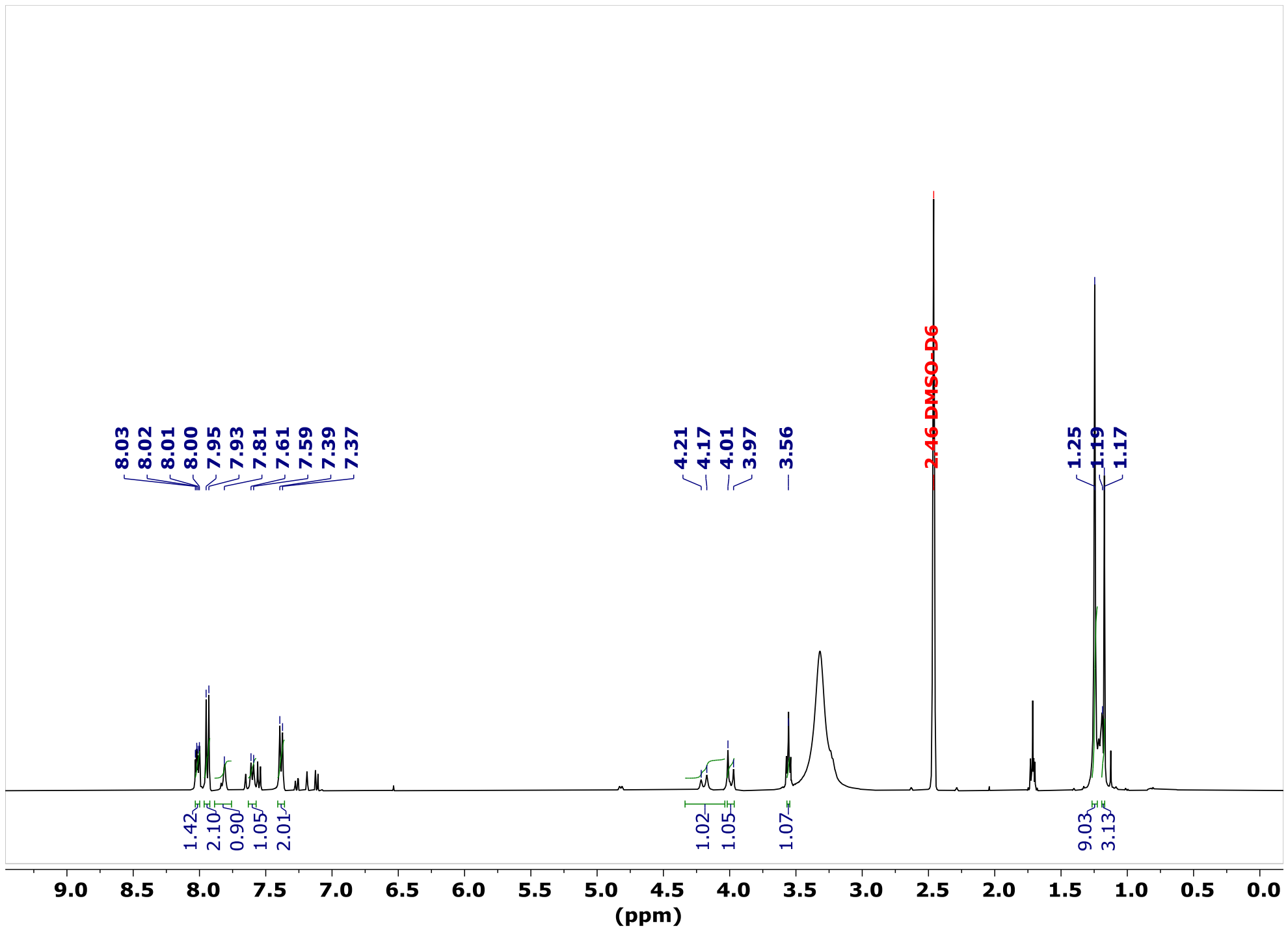

Figure S18. ^{1}H NMR spectra of H_2BPDC-Ugi-4 (DMSO-d_6, 400 MHz).

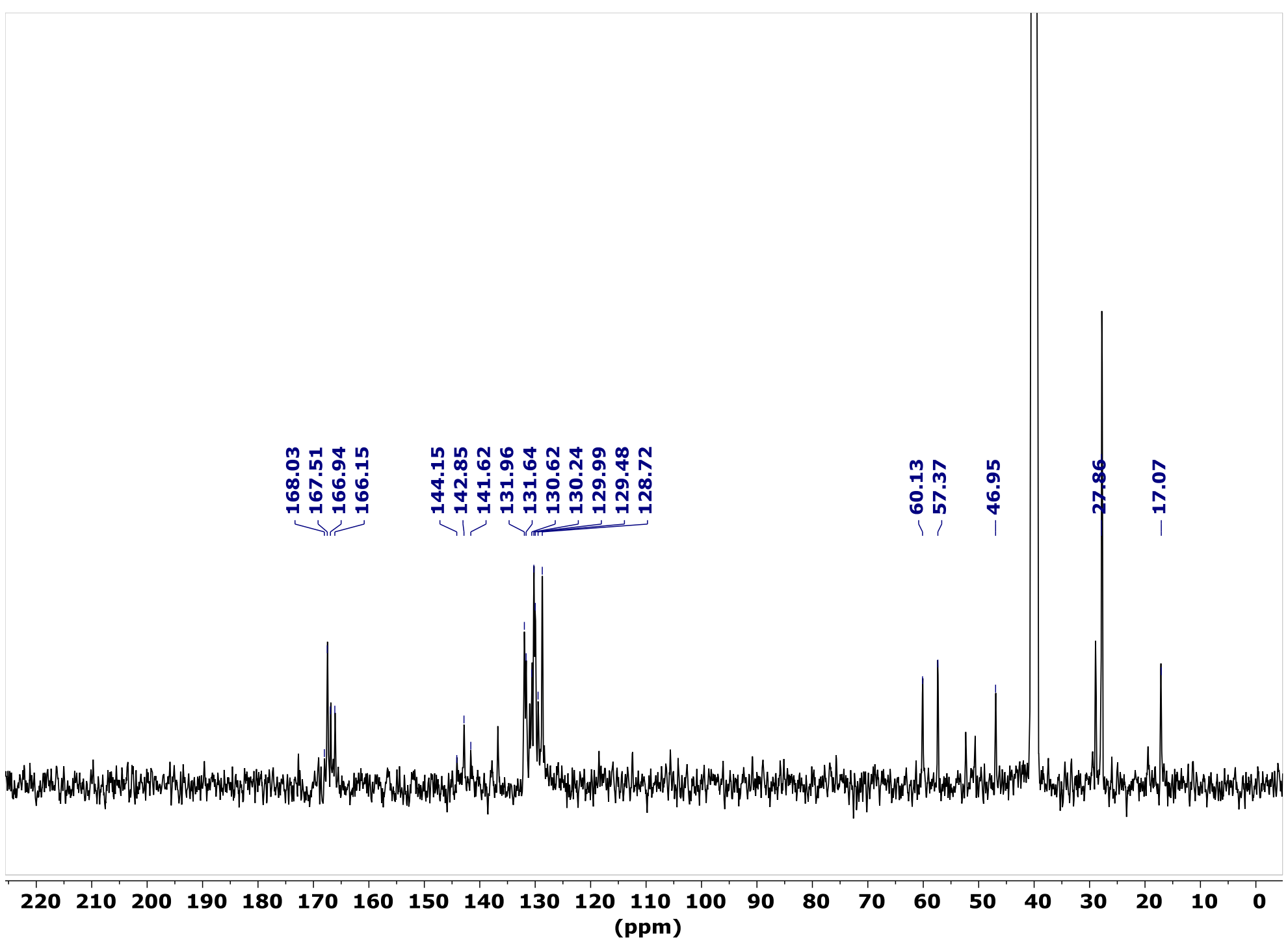

Figure S19. ^{13}C NMR spectra of H_2BPDC-Ugi-4 (DMSO-d_6, 100 MHz).

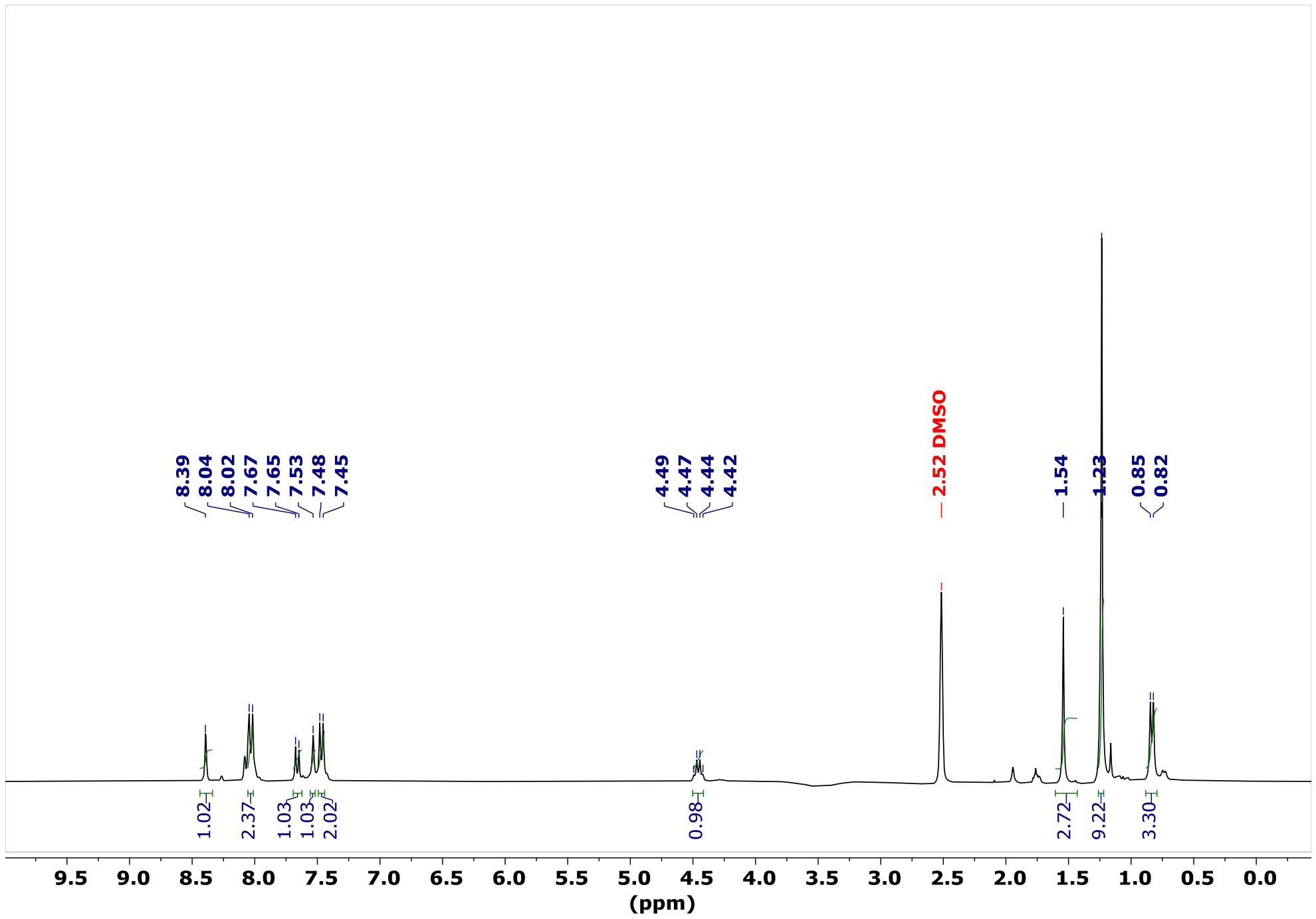

Figure S20. ^{1}H NMR spectra of H_2BPDC-Ugi-5 (DMSO-d_6, 400 MHz).

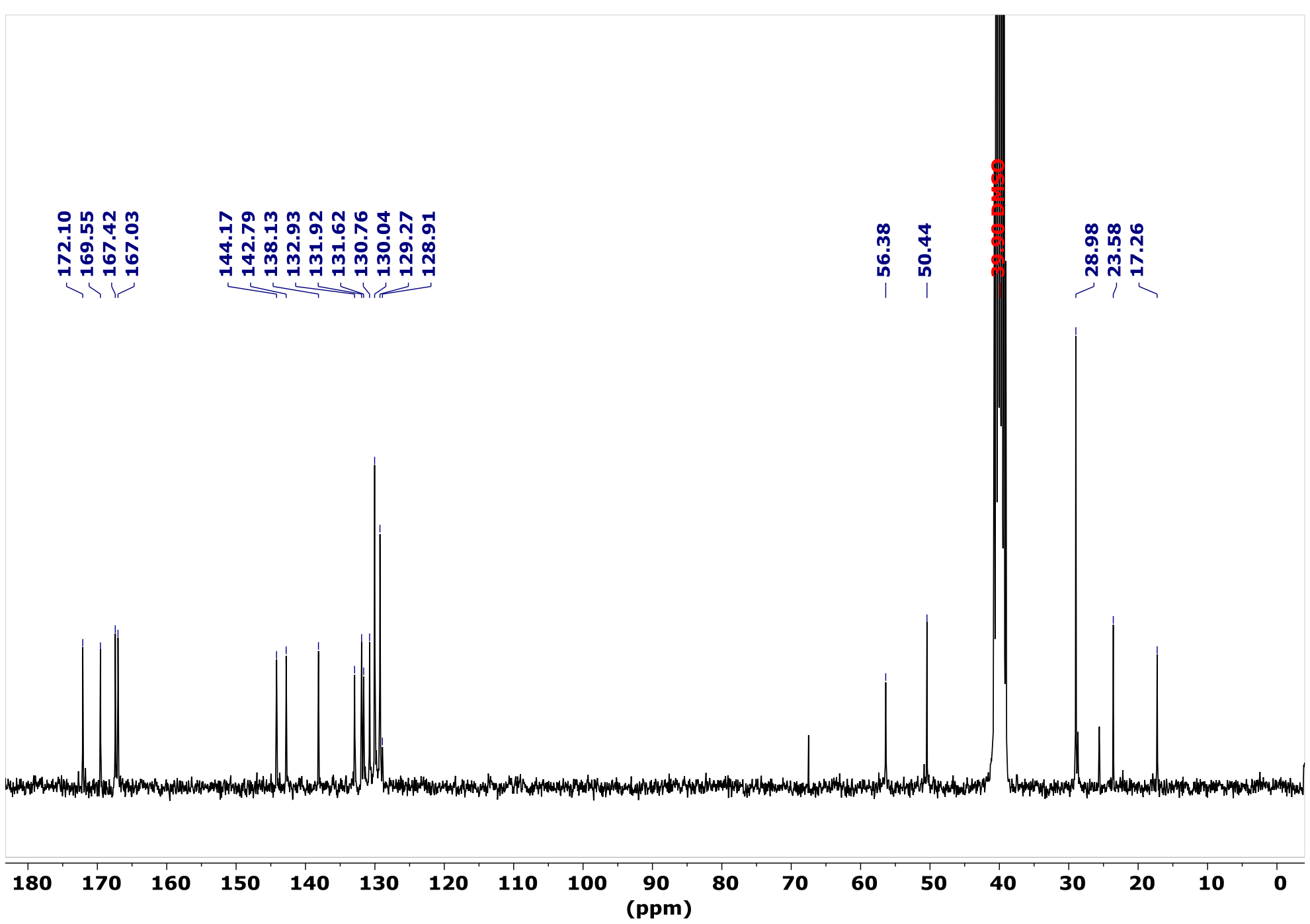

Figure S21. ^{13}C NMR spectra of H_2BPDC-Ugi-5 (DMSO-d_6, 100 MHz).

3.3 DPBDC Ugi adducts and organic linkers FT-IR spectra

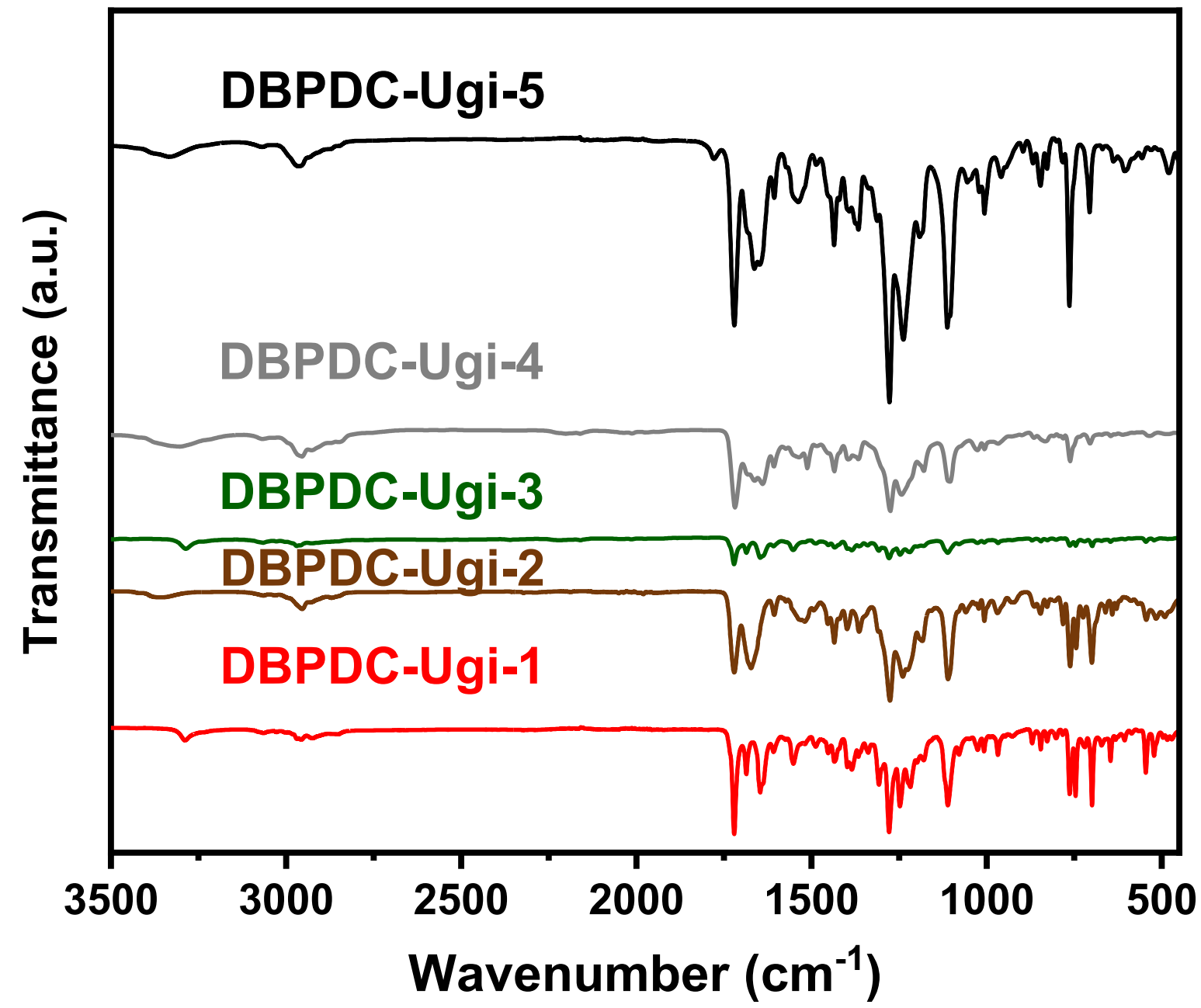

Figure S22. ATR-FTIR spectra of the esters with the DBPDC-Ugi adducts.

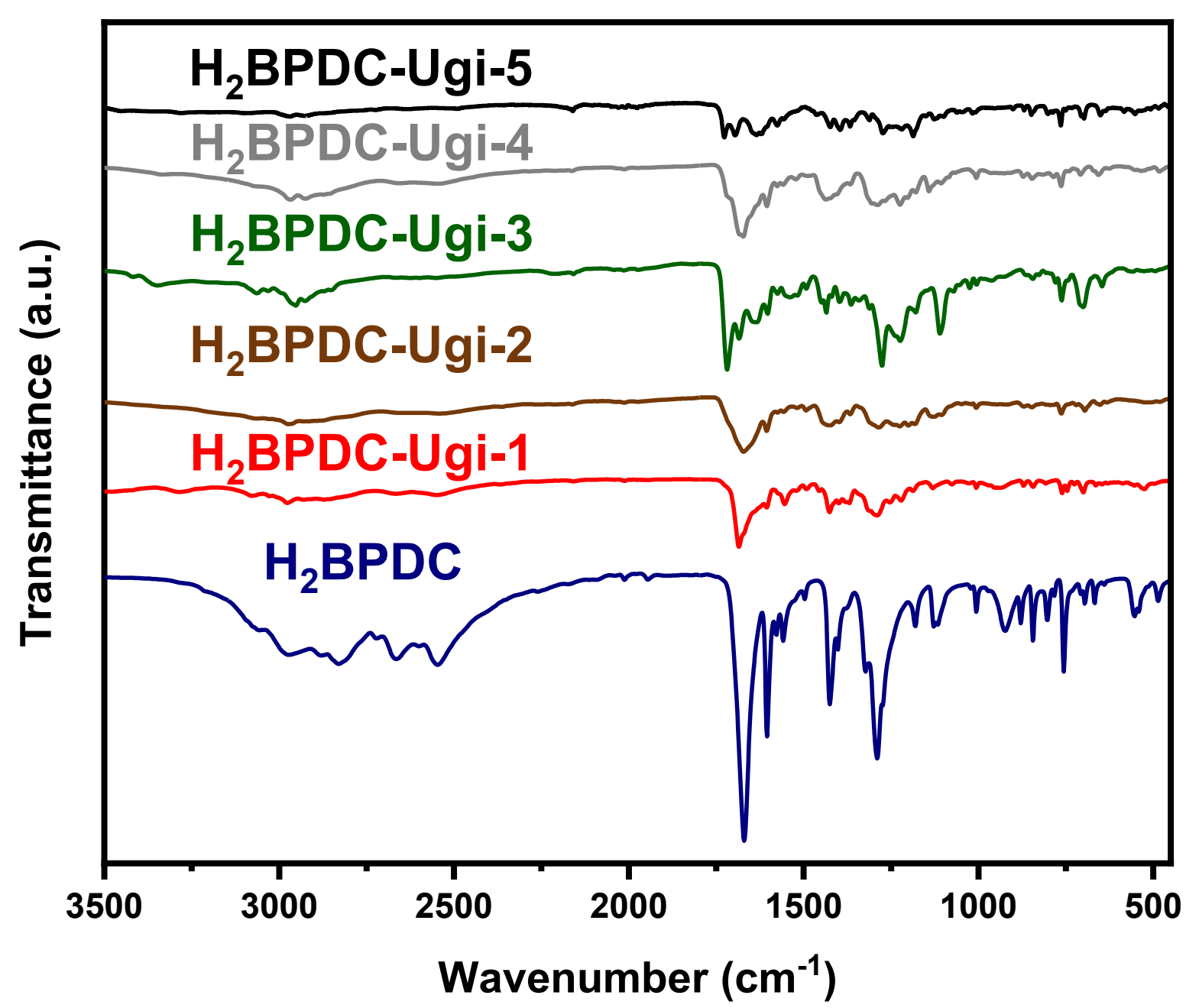

Figure S23. ATR-FTIR spectra of the organic linkers functionalized by the Ugi reaction.

3.4 Solid-state ^{13}C CPMAS NMR spectra of organic linkers.

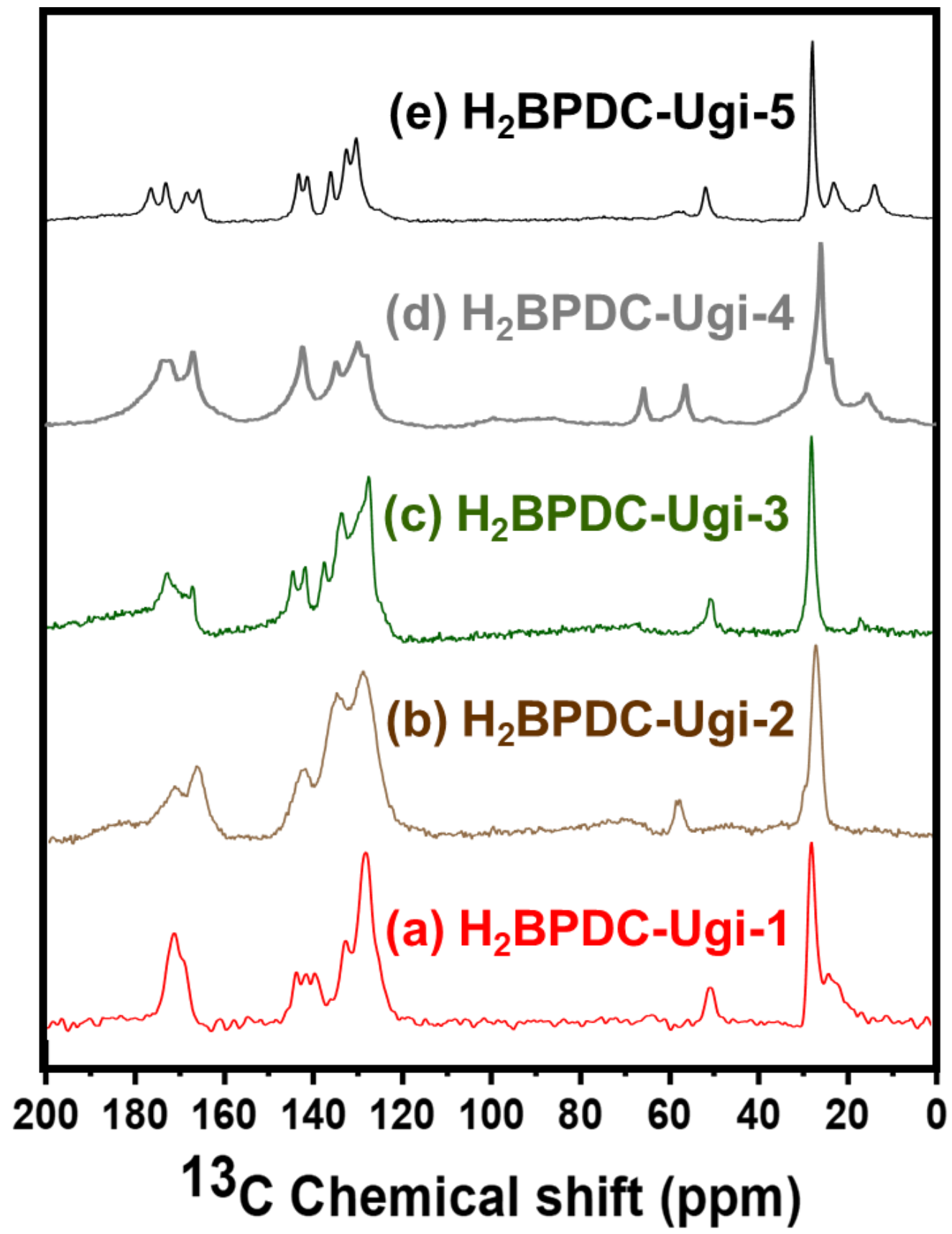

Figure S24. Solid-state ^{13}C CP-MAS NMR spectra of organic linkers.

4. MOFs Synthetic Procedures

4.1 Synthesis of UiO-67.

The synthesis was carried out in 20 mL screw-capped glass vials. $ZrCl_4$ (55.92 mg, 0.24 mmol) and [1,1′-biphenyl]-4,4′-dicarboxylic acid (58.14 mg, 0.24 mmol) were added to the vial, followed by the addition of trifluoroacetic acid (45 equiv, 4.132 mL of a 2.6 M solution in DMF) and hydrochloric acid (4 equiv, 0.800 mL of a 1.2 M solution in DMF). Additional DMF was added to reach a total solvent volume of 15 mL. The vial was sealed and heated in an oven at 120 °C for 72 h. After cooling to room temperature, the supernatant was removed, and the resulting solid was washed with DMF (3×). The washing procedure was repeated using chloroform (3×). The solid was collected by vacuum filtration and subsequently activated under vacuum at 160 °C for 12 h. This synthetic procedure was adapted from previously reported methods for the preparation of UiO-type MOFs.[12,13]

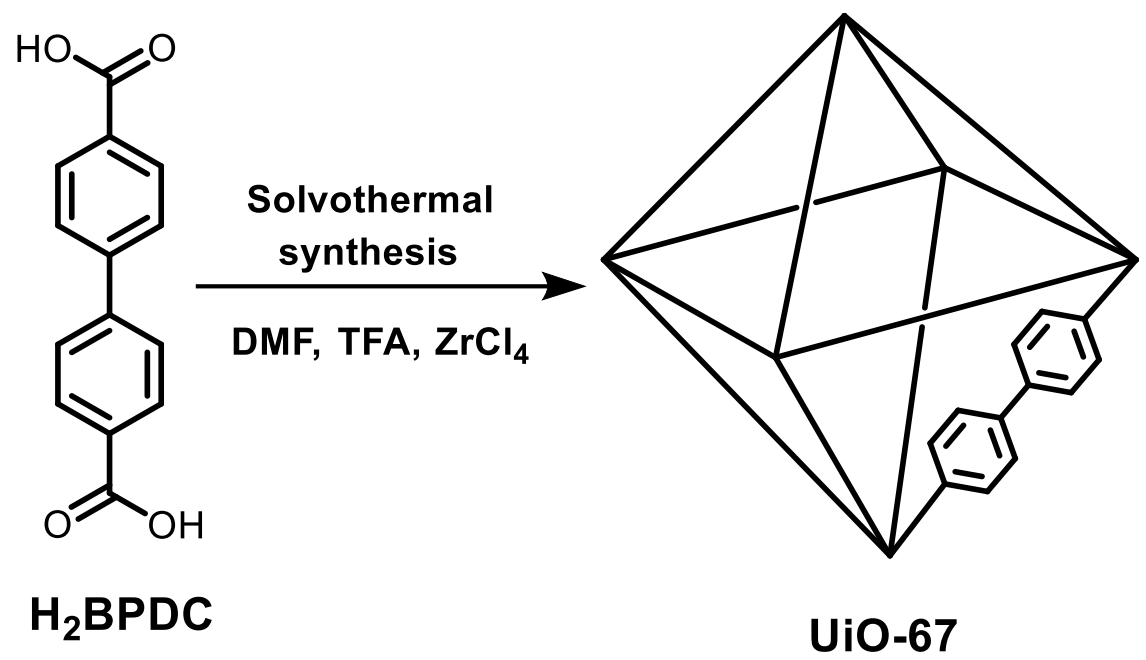

Scheme S3. Synthesis of UiO-67 from the H_2BPDC linker.

4.2 General procedure for the synthesis of Ugi-functionalized MOFs

The synthesis was carried out in 20 mL screw-capped glass tubes. $ZrCl_4$ (55.92 mg, 0.24 mmol) was added, and exclusively functionalized organic linkers were used for MOF formation: H_2BPDC-Ugi-1 (117.25 mg, 0.24 mmol) for MOF Ugi-1A, H_2BPDC-Ugi-2 (116.76 mg, 0.24 mmol) for MOF Ugi-2A, H_2BPDC-Ugi-4 (101.82 mg, 0.24 mmol) for MOF Ugi-4A, and H_2BPDC-Ugi-5 (102.35 mg, 0.24 mmol) for MOF Ugi-5A. Trifluoroacetic acid (45 equiv, 4.132 mL of a 2.6 M solution in DMF) was then added, and additional DMF was used to adjust the total reaction volume to 15 mL. The tubes were sealed and heated in an oven preheated to 120 °C for 72 h. After cooling to room temperature, the supernatant was removed, and the resulting solids were washed with fresh DMF (3×) followed by chloroform (3×). The solids were collected by vacuum filtration and activated under vacuum at 160 °C for 12 h to remove residual solvent. *Attempts to synthesize MOF Ugi-3A using exclusively the H_2BPDC-Ugi-3 linker were unsuccessful due to the low solubility of the linker, which prevented proper crystallization.

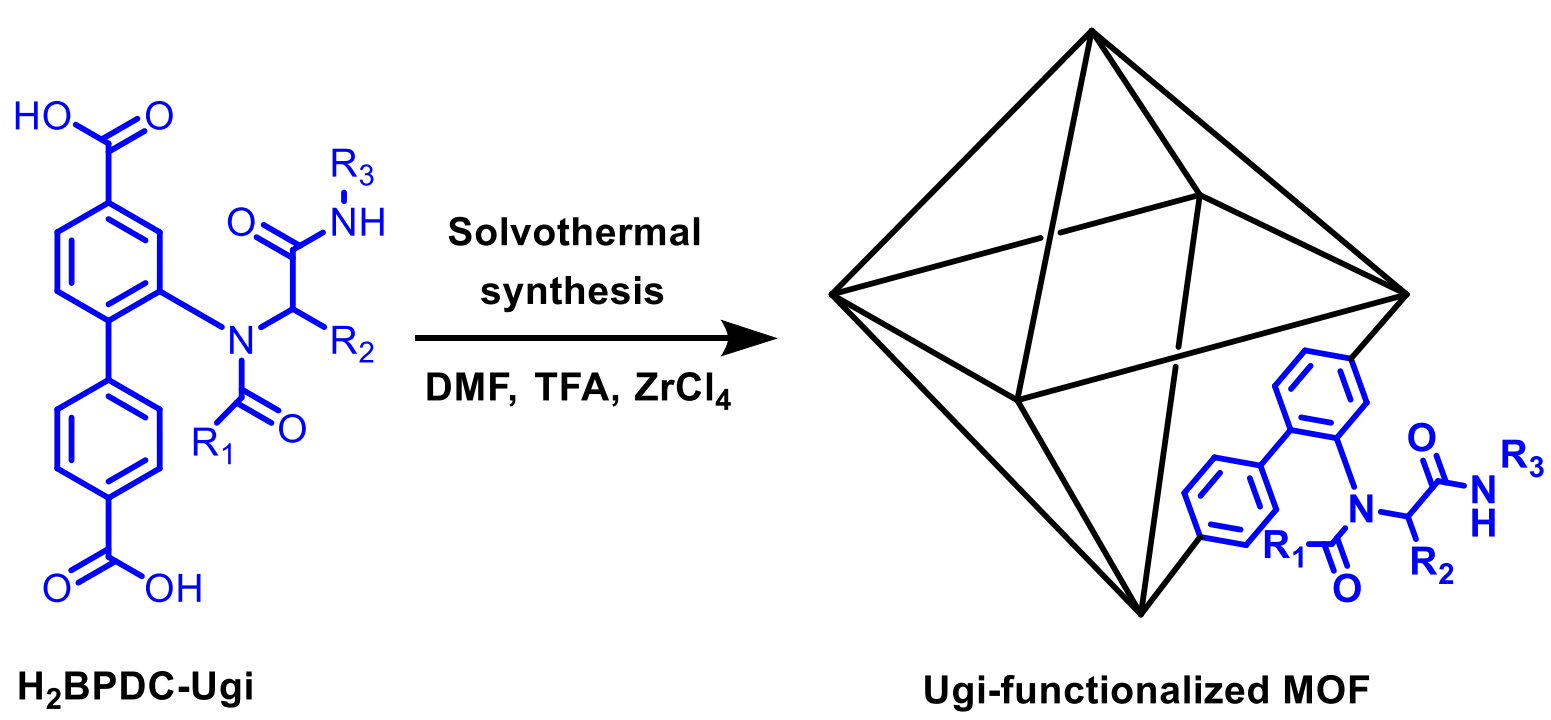

Scheme S4. Synthesis of UiO-67-Ugi from the L-Ugi linker.

4.3 General procedure for the synthesis of Ugi MTV-MOFs

The synthesis was carried out in 20 mL screw-cap glass tubes. For the preparation of the MTV-MOFs, $ZrCl_4$ (55.92 mg, 0.24 mmol) was added, and mixtures of functionalized and nonfunctionalized organic linkers were employed in equimolar amounts (50:50). Specifically, the following linker combinations were used: H_2BPDC-Ugi-1 (58.62 mg, 0.12 mmol) and H_2BPDC (29.07 mg, 0.12 mmol) for MOF Ugi-1B; H_2BPDC-Ugi-2 (58.38 mg, 0.12 mmol) and H_2BPDC (29.07 mg, 0.12 mmol) for MOF Ugi-2B; H_2BPDC-Ugi-3 (66.07 mg, 0.12 mmol) and H_2BPDC (29.07 mg, 0.12 mmol) for MOF Ugi-3B; and H_2BPDC-Ugi-4 (50.91 mg, 0.12 mmol) and H_2BPDC (29.07 mg, 0.12 mmol) for MOF Ugi-4B. Trifluoroacetic acid (45 equiv, 4.132 mL of a 2.6 M solution in DMF) was then added, and additional DMF was used to adjust the total reaction volume to 15 mL. The tubes were sealed and heated in an oven preheated to 120 °C for 72 h. After cooling to room temperature, the supernatant was removed, and the resulting solids were washed with DMF (3×) followed by chloroform (3×). The solids were collected by vacuum filtration and activated under vacuum at 160 °C for 12 h to remove residual solvent from the pores.

It should be noted that MOF Ugi-5B could not be obtained under the optimized conditions; characterization of the resulting solid indicated selective incorporation of H_2BPDC without inclusion of L-Ugi-5, and this material was therefore excluded from further analysis.

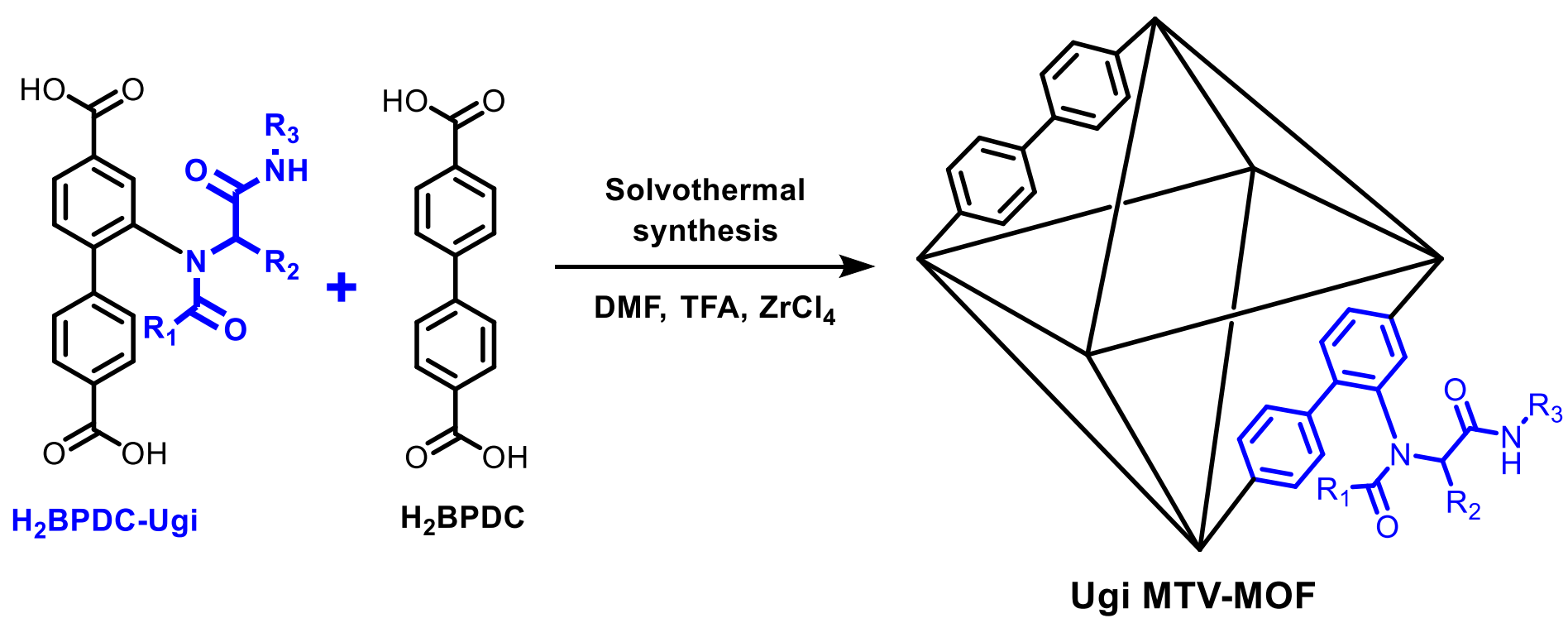

Scheme S5. Synthesis of MTV-UiO-67-Ugi from the H_2BPDC-Ugi and H_2BPDC linkers.

5. Characterization of MOFs

5.1 Ugi-functionalized MOFs characterizations

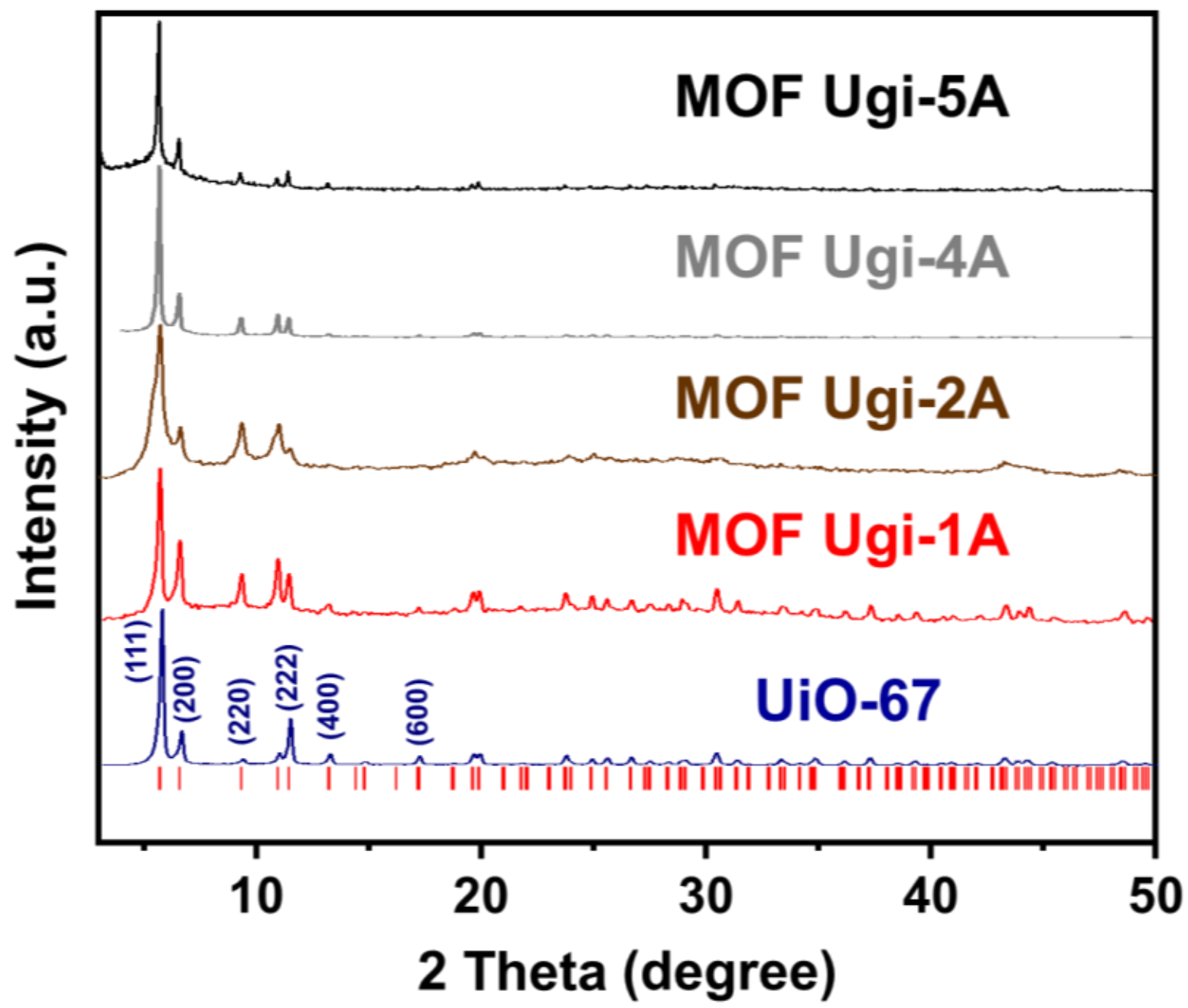

Figure S25. PXRD patterns of Ugi-functionalized MOFs recorded in the 2θ range of 5–50°.

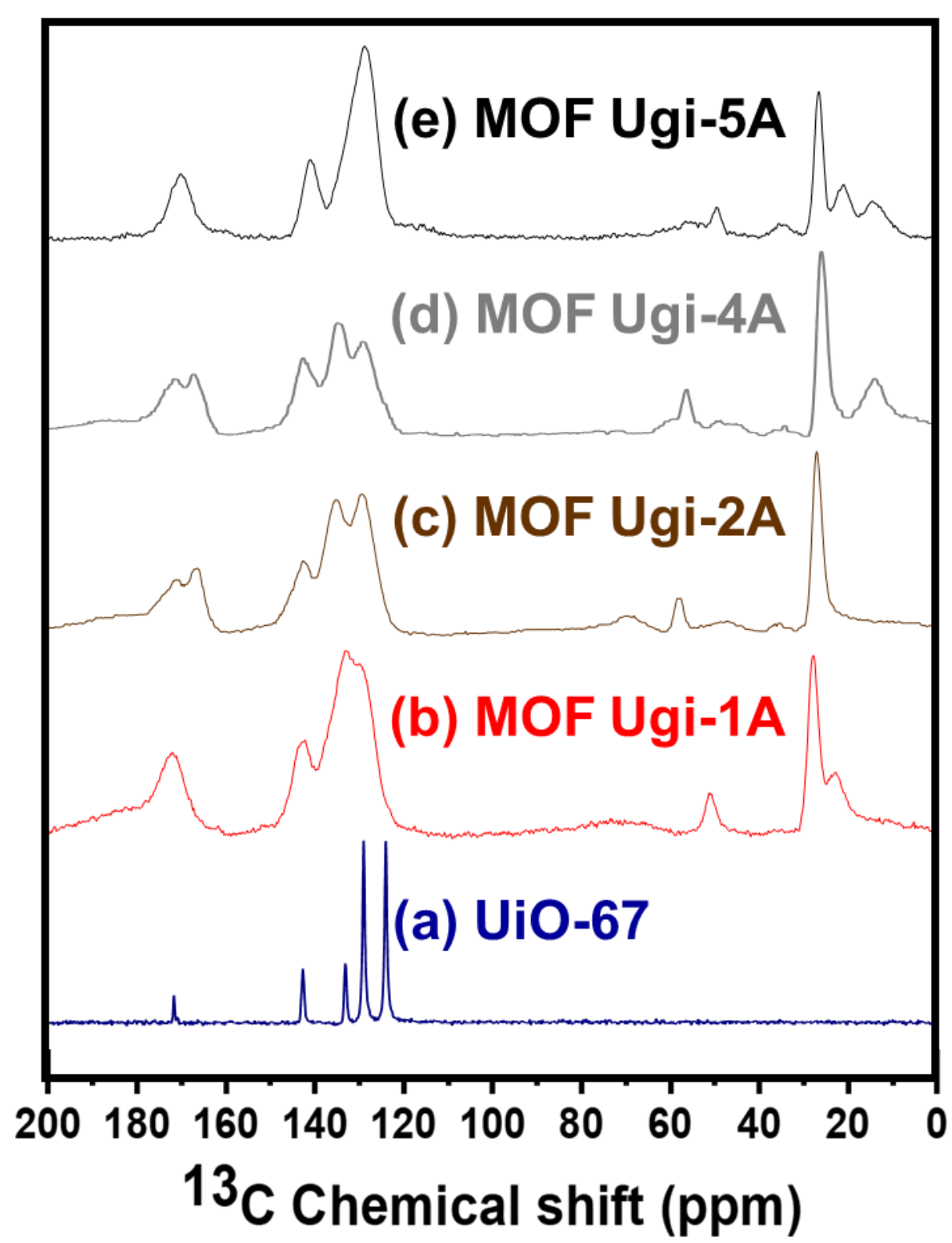

Figure S26. Solid-state ^{13}C CP-MAS NMR spectra of Ugi-functionalized MOFs.

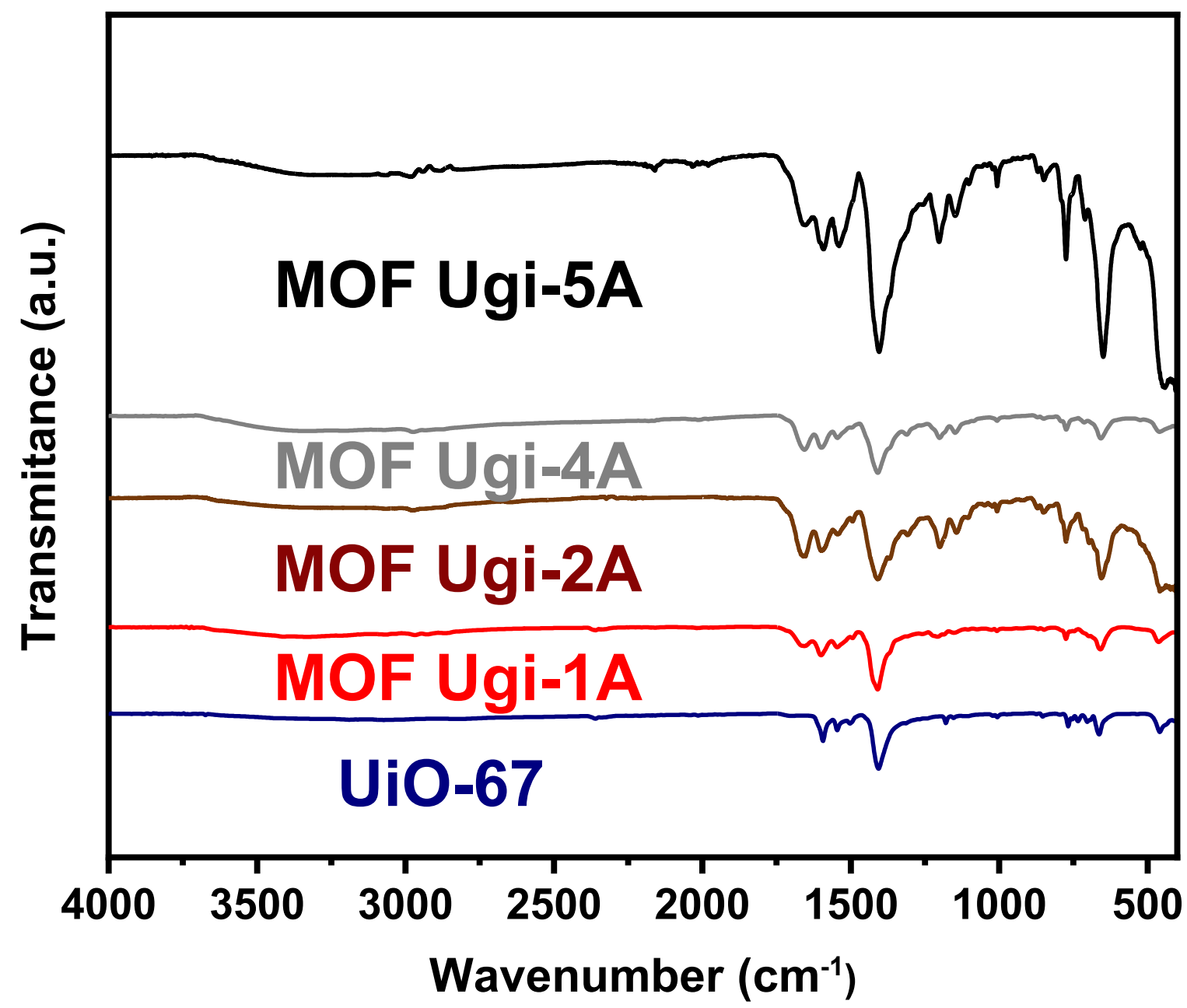

Figure S27. ATR-FTIR spectra of the Ugi-functionalized MOFs.

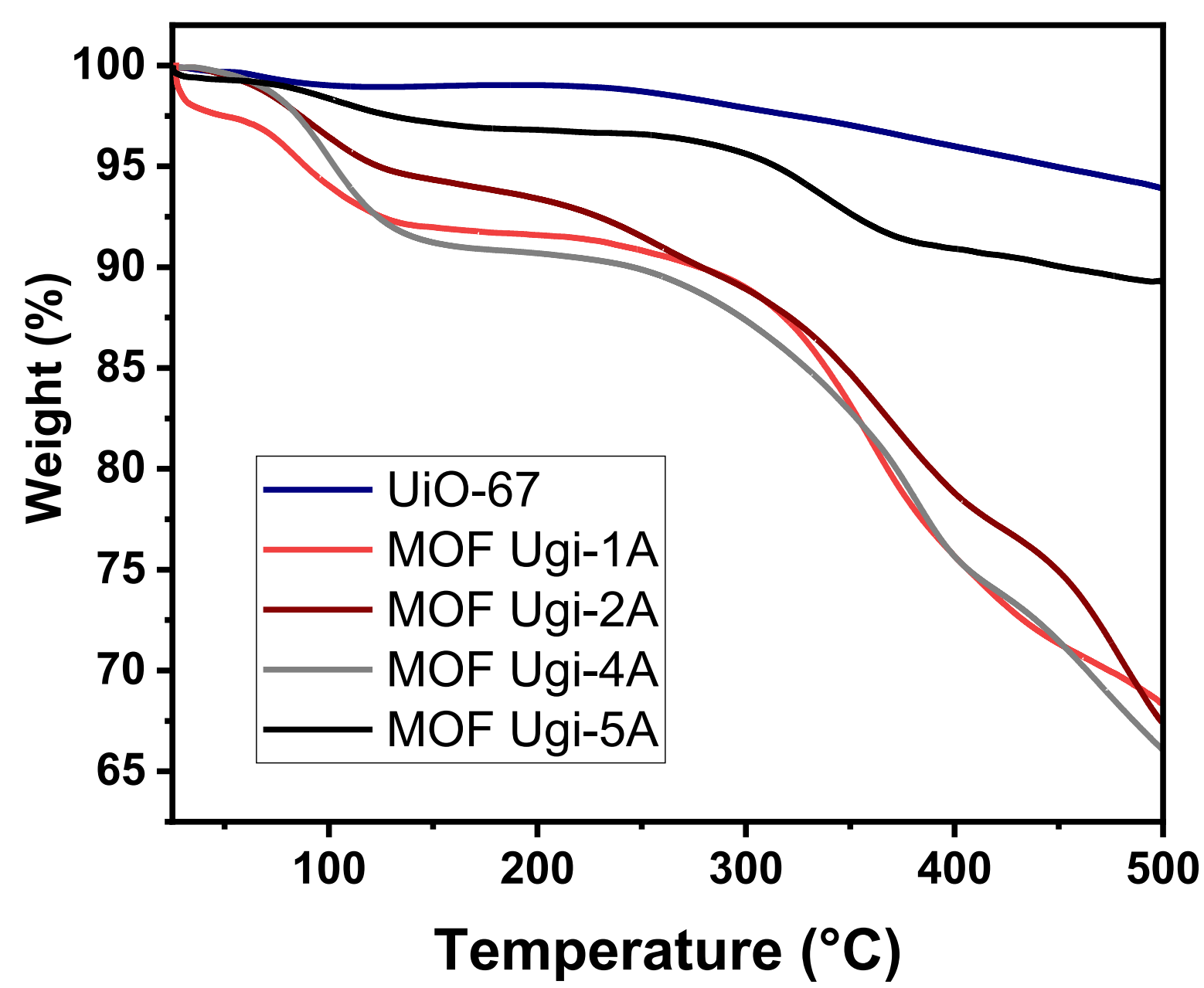

Figure S28. Thermogravimetric analysis (TGA) curves of pristine UiO-67 and Ugi-functionalized MOFs under a nitrogen atmosphere.

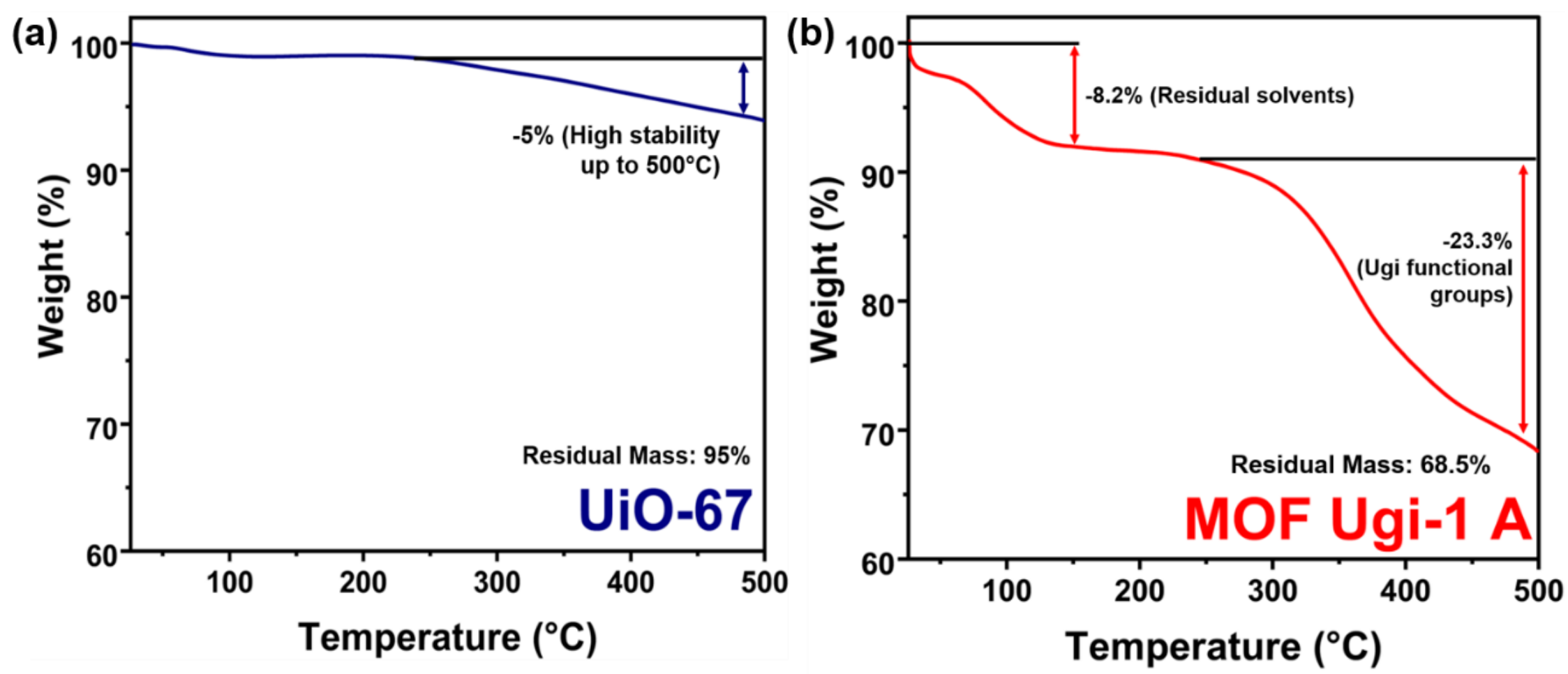

Figure S29. TGA traces (25-500 °C, N_2, 10 °C min^{-1}) of **(a)** UiO-67 and **(b)** MOF Ugi-1A.

TGA (25-500 °C, N_2, 10 °C min^{-1}; Figure S29) shows that pristine UiO-67 loses only ~5% up to 500 °C (residual solvent and water), reflecting its thermal stability, whereas MOF Ugi-1A shows two events: a 8.2% mass loss (~25-150 °C, guest water and solvent) and a 23.3% mass loss (~250-450 °C), assigned to partial decomposition of the appended Ugi moiety; the absence of an equivalent event in UiO-67 confirms this loss originates from the functionalization. As the measurements were run under N_2 up to 500 °C, decomposition is incomplete and the masses at 500 °C (95% and 68.5%) do not correspond to the inorganic ZrO_2 residue. On a desolvated basis, the -23.3% event represents ~25% of the dehydrated framework mass, consistent with the ~37% expected for the pendant Ugi groups at 86% functionalization (Table S7), since only part of the appendage decomposes below 500 °C.

5.2 Ugi MTV-MOFs characterizations

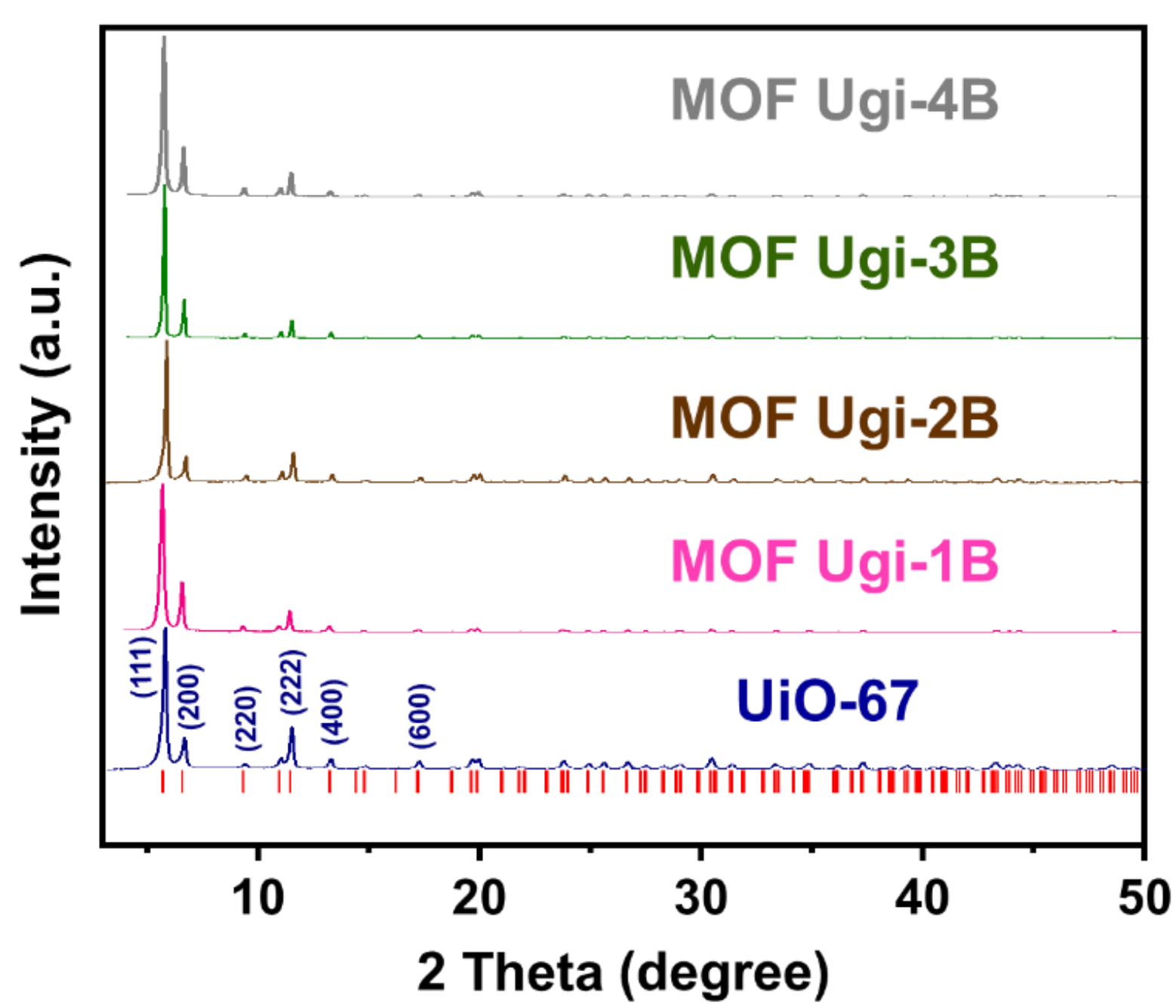

Figure S30. PXRD patterns of Ugi MTV-MOFs recorded in the 2θ range of 5-50°.

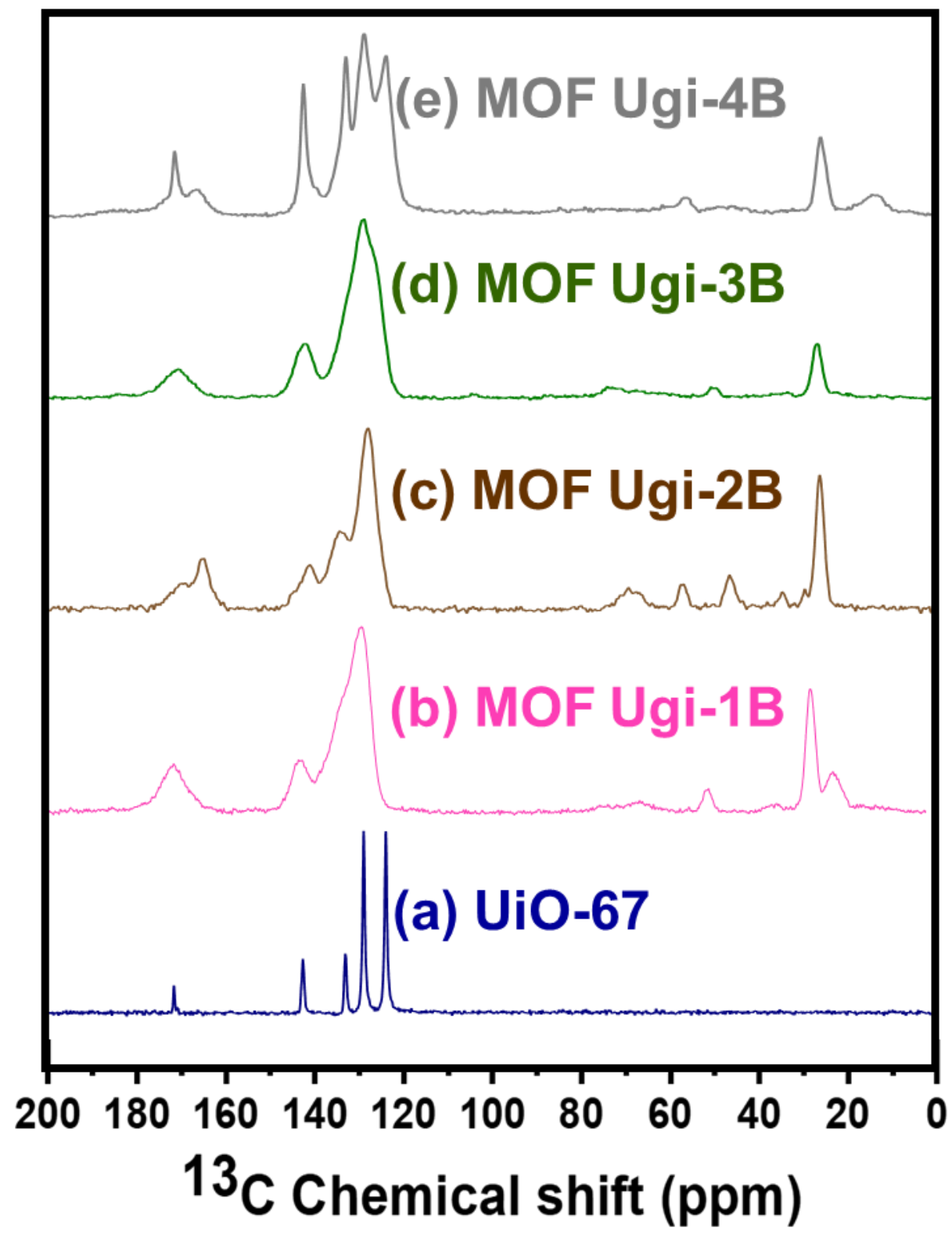

Figure S31. Solid-state ^{13}C CP-MAS NMR spectra of Ugi MTV-MOFs.

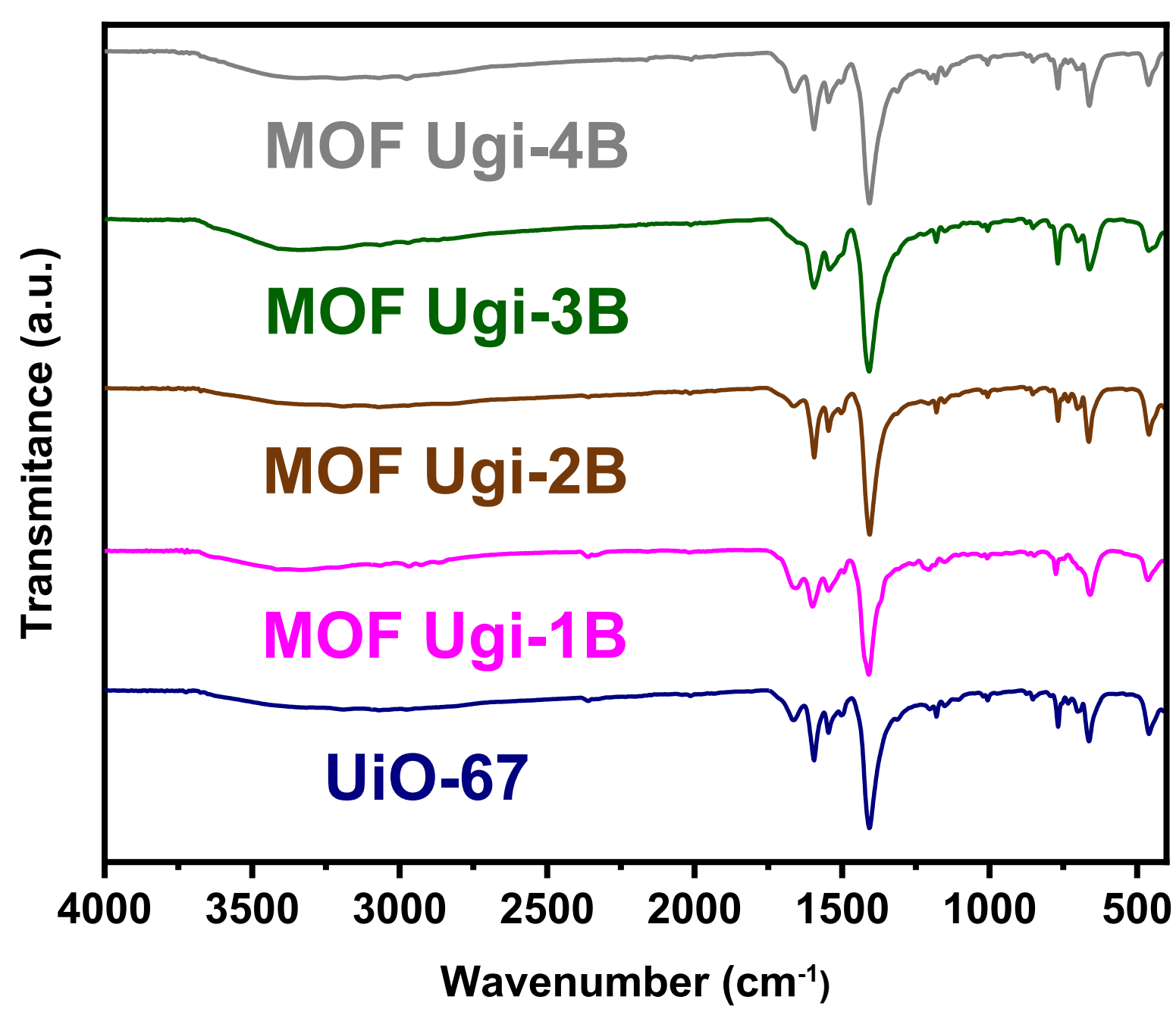

Figure S32. ATR-FTIR spectra of the Ugi MTV-MOFs.

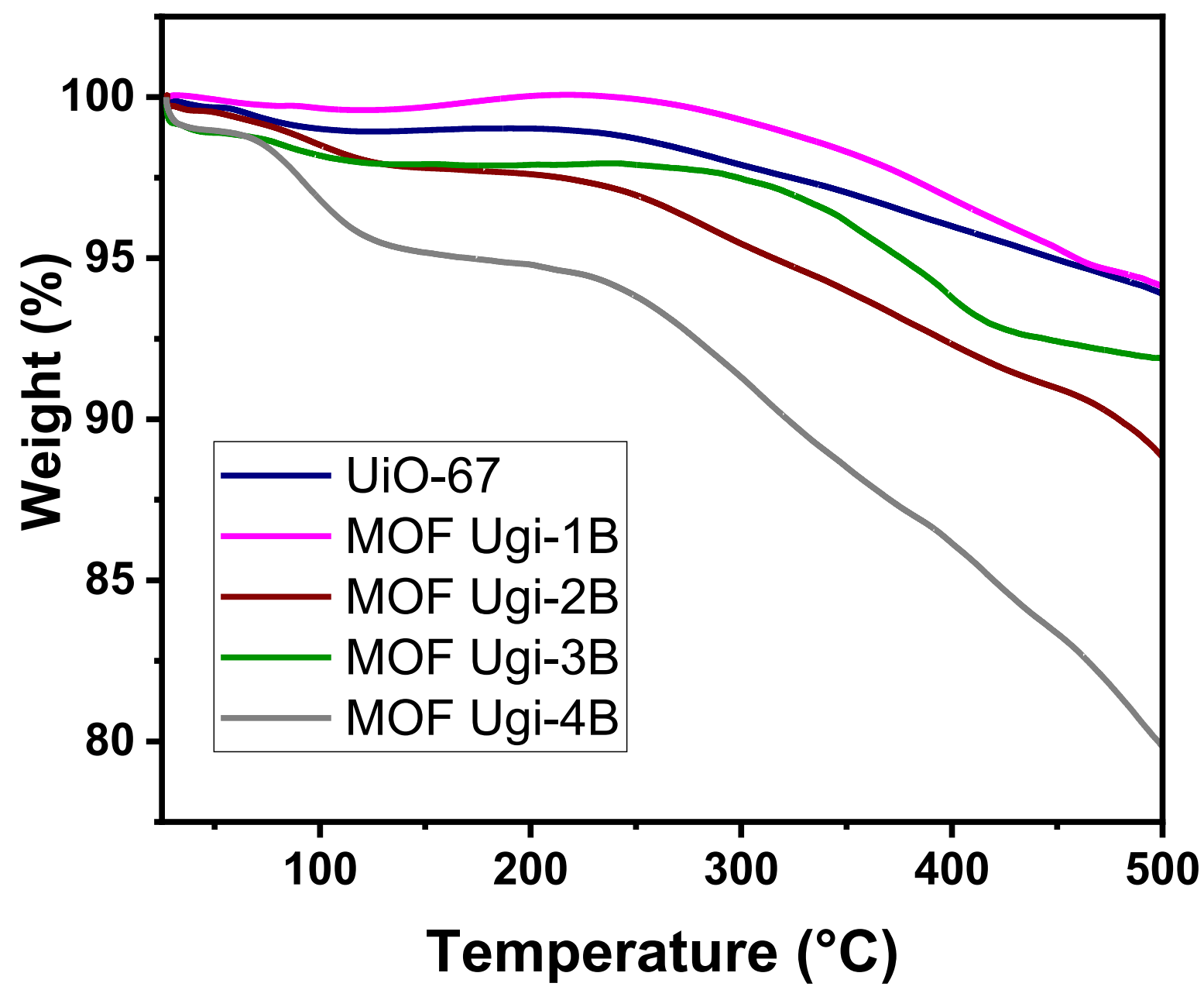

Figure S33. Thermogravimetric analysis (TGA) curves of pristine UiO-67 and Ugi MTV-MOFs under a nitrogen atmosphere.

5.3 Le Bail Refinement of the Ugi-1 Series of MOFs

Le Bail refinements of the PXRD patterns of the functionalized UiO-67 MOFs were performed using the crystallographic information file (CIF) of UiO-67 reported in the literature as the structural reference.[13]

Table S3. Crystallographic parameters obtained from Le Bail refinements of UiO-67 and Ugi-1 series functionalized MOFs.

**UiO-67-reported**	Fm-3m<br>a= 26.7734(2)Å<br>V= 19191.5(5)Å^3	-
**UiO-67**	Fm-3m<br>a= 26.813(1)Å<br>V= 19276(1)Å^3	R_{exp}: 1.85<br>R_B: 4.52<br>R_{wp}: 5.78<br>S: 3.12
**MOF Ugi-1A**	Fm-3m<br>a= 26.674(4)Å<br>V= 18979(5)Å^3	R_{exp}: 8.59<br>R_B: 11.0<br>R_{wp}: 15.1<br>S: 1.76
**MOF Ugi-1B**	Fm-3m<br>a= 26.789(2)Å<br>V= 19226(3)Å^3	R_{exp}: 4.00<br>R_B: 6.03<br>R_{wp}: 7.60<br>S: 1.9

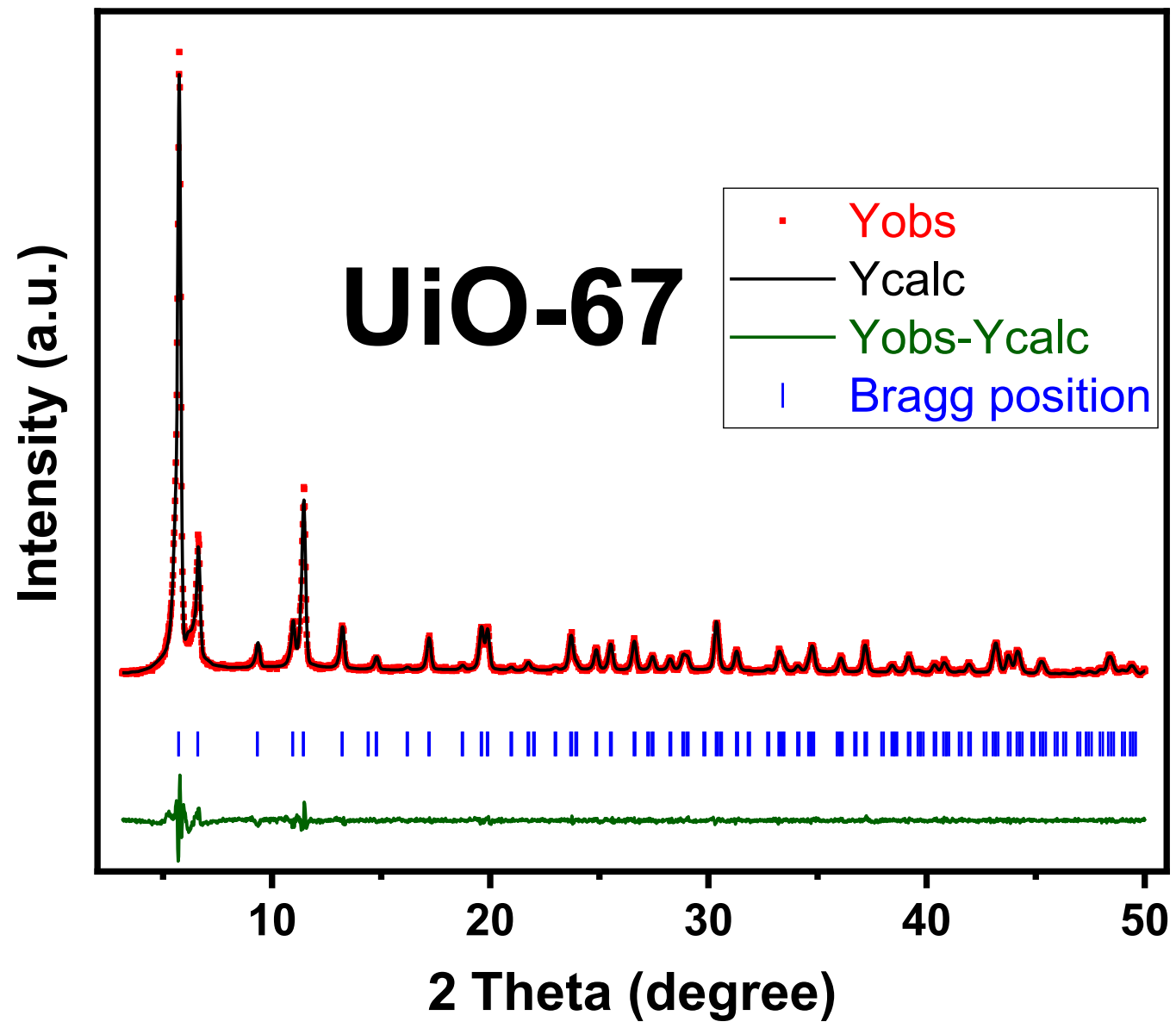

Figure S34. Le Bail refinement of the PXRD pattern for pristine **UiO-67**

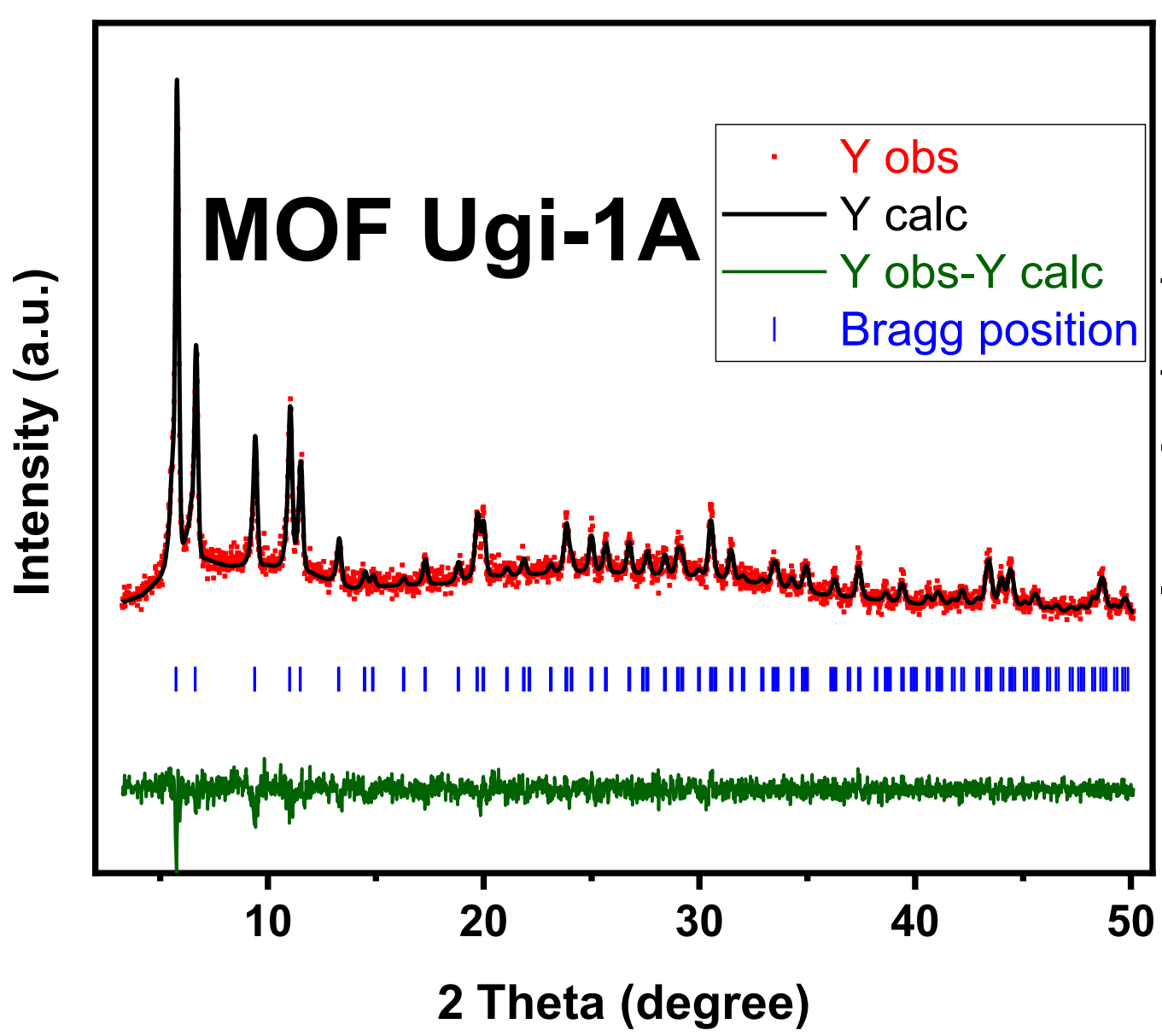

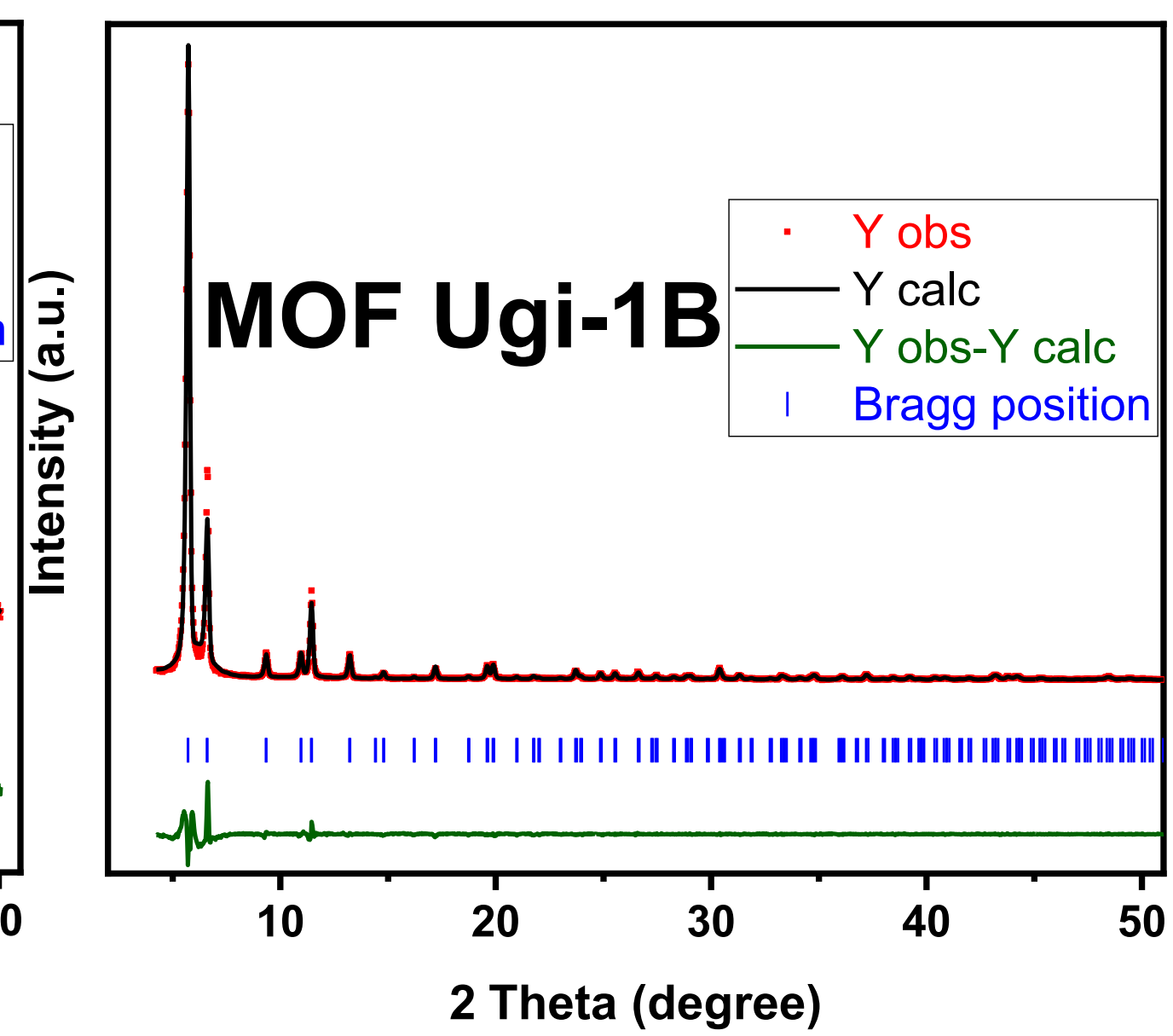

Figure S35. Le Bail refinement of the PXRD patterns for the **MOF Ugi-1A** and **MOF Ugi-1B**.

5.4 Rietveld Refinements of MOF Ugi-1 Series

Table S4. Details of the Rietveld refinement process for the materials under study.

	UiO-67-pristine		MOF Ugi-1A		MOF Ugi-1B		UiO67-reported
Data collection							
Diffractometer	Ultima IV (from Rigaku)						
Detector	Dtex ultra						
Wavelength (Å)	CuK_a: 1.54183						
2θ range (°)	3.0-50.0						
Step size (°)	0.02						
Scan step	10°/min						
Unit cell							
Space Group	Fm-3m		Fm-3m		Fm-3m		Fm-3m
Cell Parameter (Å)	a= 26.811(12)		26.67(4)		26.791(3)		26.7734(2)
V($Å^3$)	19271(14)		18979(9)		19230(3)		19191.5(5)
Z	4		4		4		4
Refinement							
# of reflections	118		118		118		
# of refined parameters	Structural	Profile	Structural	Profile	Structural	Profile	
	30	14	30	14	30	14	
R_{exp}	1.85		8.60		7.01		
R_{wp}	8.54		26.6		24.2		
R_B	5.77		16.4		16.03		
S	4.61		3.09		3.45		

Table S5. Atomic positions and thermal (B_{iso}) and occupation (Occ) factors for the materials under study.

Atom	X	Y	Z	B_{iso}	Occ
Zr1	0	0	0.0916	1.6	1
O1	0.0470	0.0470	0.0470	2.4	1
O2	0.1326	0.0000	0.0722	2.4	1
C4	0.1151	0	0.1151	3.7	0.97
C5	0.1558	0	0.1558	3.7	0.97
C6	0.2302	0	0.2302	3.7	0.97
C2	0.2420	0.0155	0.1813	3.7	0.485
C3	0.2047	0.0155	0.1441	3.7	0.485
H2	0.2798	0.0274	0.1723	5.6	0.485
H3	0.2138	0.0274	0.1062	5.6	0.485
Ow1	0.3002	0.3002	0.3002	3.9	0.69
Ow2	0.1189	0.3811	0.3811	3.9	0.80
Ow3	0.3480	0.3007	0.1520	3.9	0.75

5.5 Nitrogen adsorption analysis

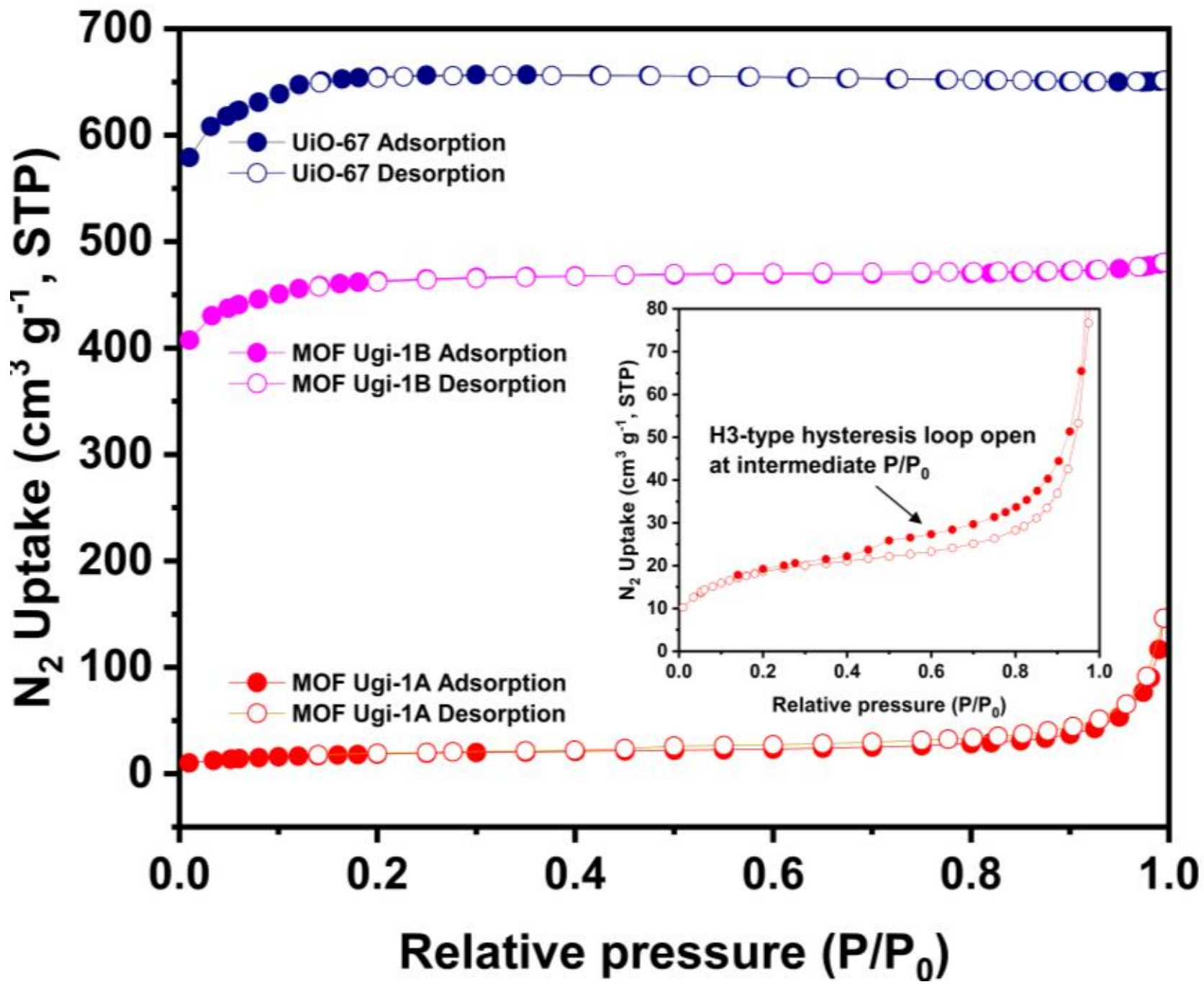

Figure S36. Nitrogen adsorption of every synthesized material with a close-up on the hysteresis loop of MOF Ugi-1A.

Table S6. Surface Area and Porosity Characteristics of **UiO-67** and **Ugi-Functionalized MOFs**.

Porous material	BET Surface Area ($m^2 g^{-1}$)	Langmuir Surface Area ($m^2 g^{-1}$)	Total pore volume ($cm^3 g^{-1}$)	Average pore diameter (nm)
**MOF-Ugi-1A.**	63.2	96.5	0.11	22.3
**MOF-Ugi-1B.**	1417	2062.7	0.73	3.3
**UiO-67**	2000	2905	1.00	2.6

Nitrogen adsorption–desorption measurements at 77 K were performed to evaluate the textural properties of the porous materials, including specific surface area, total pore volume, and average pore diameter.[14] The BET- and Langmuir-derived parameters summarized in Table S_4 reveal pronounced differences in porosity among the fully functionalized, multivariate, and pristine frameworks, highlighting the impact of linker functionalization on the accessible pore space.[14]

MOF-Ugi-1A exhibits a markedly reduced BET surface area (63.2 $m^2 g^{-1}$) and total pore volume (0.11 $cm^3 g^{-1}$), indicating a severe attenuation of permanent porosity. This pronounced decrease in accessible surface area suggests that the bulky Ugi-derived functional groups substantially obstruct the pore channels and internal cavities, thereby limiting nitrogen uptake.[15] The unusually large average pore diameter (22.3 nm) derived from the adsorption data is consistent with a highly restricted adsorption regime, in which gas uptake is dominated by interparticle voids or mesoporous textural contributions rather than by the intrinsic microporosity of the UiO-67 framework.[16]

The nitrogen isotherm of MOF Ugi-1A exhibits a hysteresis loop of type H3 according to the IUPAC classification, this is consistent with the extensive incorporation of bulky Ugi linker blocks in the intrinsic micropores of the UiO-67 framework, the residual porosity arises predominantly from interparticle and external voids formed between irregularly aggregated crystallites, which is in agreement with the loss of facet definition and increased surface roughness observed by optical microscopy (**Figure 3c**).

In contrast, the multivariate material **MOF-Ugi-1B** displays a significantly higher BET surface area (1417 m^2 g^{-1}) and total pore volume (0.73 cm^3 g^{-1}), indicating that a substantial fraction of the intrinsic porosity is preserved upon partial incorporation of the functionalized linker.[17] The average pore diameter (3.3 nm) is slightly larger than that of pristine UiO-67 (2.6 nm), suggesting moderate pore modification while maintaining a predominantly microporous structure.[18] These results demonstrate that dilution of the bulky Ugi-functionalized linker within the framework effectively mitigates pore blockage effects, enabling enhanced accessibility of the internal surface area.[18]

Pristine **UiO-67** exhibits the highest BET and Langmuir surface areas (2000 and 2905 m^2 g^{-1}, respectively) as well as the largest total pore volume (1.00 cm^3 g^{-1}), consistent with a highly porous framework and serving as a benchmark for assessing the impact of linker functionalization.[19] The progressive decrease in surface area and pore volume from UiO-67 to MOF-Ugi-1B and MOF-Ugi-1A underscores the strong dependence of the textural properties on both the nature and proportion of functionalized linkers incorporated into the framework.[14,18] Overall, these gas sorption results demonstrate that while complete functionalization with bulky Ugi-derived linkers leads to a severe loss of accessible porosity, partial incorporation within a multivariate MOF architecture enables a balance between framework functionalization and retention of permanent porosity, thereby providing a tunable platform for controlling pore accessibility in UiO-67-type materials.[18,20]

The broad, featureless BJH distribution of MOF Ugi-1A (~5-200 nm, no defined maximum; **Figure S37**) indicates that the 22.3 nm value is a volume-weighted mean of heterogeneous extra-framework voids rather than discrete cages. This is corroborated by the retention of the *fcu* topology with a slightly contracted unit cell (a = 26.674(4) Å), incompatible with cavities of tens of nanometers, and by the collapse of the BET surface area (63 vs 1417 m^2 g^{-1} for MOF Ugi-1B), indicating that the intrinsic micropores are blocked rather than enlarged. Consistently, the nitrogen isotherm does not plateau even at $P/P_0 = 0.995$, supporting condensation in large external voids.

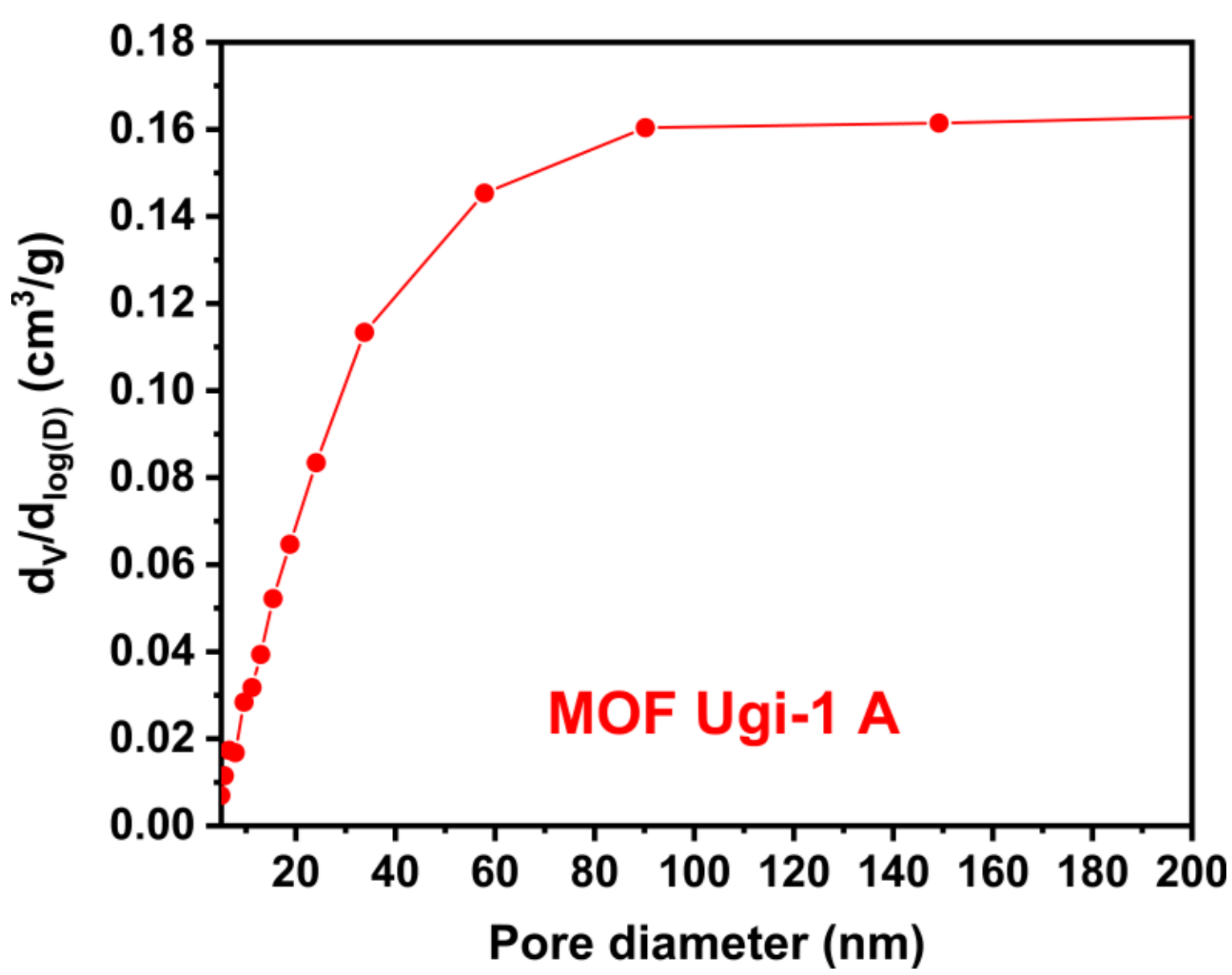

Figure S37. Pore size distribution graph of MOF Ugi-1A.

5.6 Reduction in accessible porosity

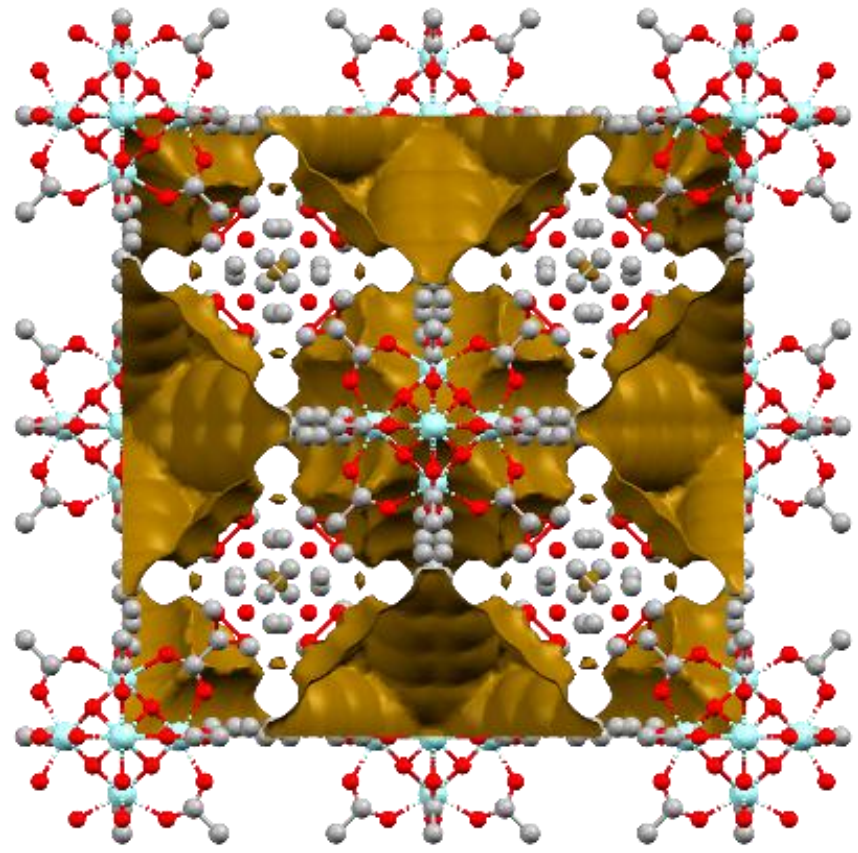

Figure S38. Geometric void analysis and solvent-accessible volume of pristine UiO-67 calculated using a spherical probe radius of 1.2 Å.

To further rationalize the reduction in accessible porosity observed by N_2 adsorption, the solvent-accessible volume of pristine **UiO-67** was evaluated by geometric void analysis using a spherical probe radius of 1.2 Å. This analysis was performed using the crystallographic information file (CIF) of UiO-67 reported[13] and therefore represents an idealized structural model that does not explicitly account for framework defects, linker disorder, or dynamic structural effects. The pristine framework exhibits solvent-accessible voids corresponding to approximately 19.2% of the unit cell volume (3688 Å^3), consistent with the large, well-defined cavities of the UiO-67 topology. The pronounced decrease in BET surface area and pore volume observed upon functionalization can be rationalized by partial occupation of these voids by bulky Ugi-derived linkers, which are expected to reduce both pore accessibility and connectivity.

In the mixed-linker material **MOF-Ugi-1B**, the coexistence of functionalized and pristine linkers is expected to lead to partial pore filling while largely preserving long-range structural order. In contrast, the fully functionalized MOF-Ugi-1A likely exhibits a substantially greater restriction of accessible internal free volume due to more extensive steric occupation of the pore environment. Although the geometric void analysis is based on the ideal parent framework, the progressive reduction in framework-accessible voids is qualitatively consistent with the experimentally observed trends in N_2 adsorption, supporting the conclusion that increasing degrees of Ugi-based functionalization limit pore accessibility in these UiO-67-derived materials. Overall, these results suggest that Ugi-based linker functionalization modulates both porosity and pore architecture while preserving the general adsorption characteristics associated with UiO-type frameworks.

5.7 Elemental analysis of MOFs

Table S7. Elemental analysis (CHN) performed on the pristine **UiO-67** sample and the functionalized **Ugi-MOFs** and **Ugi MTV-MOFs** to confirm the incorporation of the functionalized linkers.

Material	%C	%H	%N	Theoretical %C in the MOF	Theoretical %H in the MOF	Theoretical %N in the MOF	% Degree of functionalization of the MOF
**UiO-67**	40.39	3.04	0	47.29	3.02	0	N/A
**UiO-Ugi-1A**	43.08	4.03	4.01	55.89	4.8	4.66	86.0
**UiO-Ugi-1B**	43.27	3.21	1.39	52.7	4.14	2.93	23.7
**UiO-Ugi-2A**	42.97	3.42	2.43	56.07	4.48	4.67	52.0
**UiO-Ugi-2B**	44.18	3.20	1.48	52.81	3.94	2.93	25.2
**UiO-Ugi-3B**	41.60	3.26	0.90	55.38	4.09	2.75	16.4
**UiO-Ugi-4A**	40.84	3.96	4.11	51.38	4.62	5.21	78.9
**UiO-Ugi-4B**	41.51	3.77	2.74	49.76	3.99	3.14	43.6
**UiO-Ugi-5A**	43.15	3.73	2.96	51.19	4.98	5.19	57.0

Elemental analysis (C, H, and N) was carried out to quantitatively assess the incorporation of the Ugi-functionalized linker into the UiO-67-type frameworks and to determine the actual composition of the resulting materials. In the pristine UiO-67 framework, no nitrogen-containing species are present in the organic linker or inorganic nodes; therefore, any detected nitrogen in the functionalized materials can be unambiguously attributed to the Ugi-derived moiety. As a result, the nitrogen content provides a direct and reliable probe to evaluate the degree of functionalization of the framework and to distinguish between nominal linker feed ratios and the true linker incorporation achieved during synthesis.[21] For the **Ugi-A series**, synthesized exclusively from the Ugi-functionalized linker, the experimentally determined nitrogen contents are in good agreement with the theoretical values calculated for fully functionalized frameworks. Minor deviations between experimental and theoretical C, H, and N percentages are attributed to residual solvent molecules, missing-linker defects, and/or partial framework dehydration, which are commonly observed in Zr-based UiO-type materials.[22] Nevertheless, the relatively high nitrogen contents observed for these samples indicate an efficient incorporation of the Ugi-modified linker within the framework.

In contrast, the **Ugi-B materials** were prepared using a **multivariate (MTV) approach**,[23] employing a 50 wt% mixture of the Ugi-functionalized linker and the non-functionalized H_2BPDC linker. As expected, these materials exhibit significantly lower nitrogen contents compared to their Ugi-A counterparts, reflecting the dilution of the Ugi-derived functionality within the framework. The experimentally determined nitrogen percentages confirm the successful co-incorporation of both linkers and allow the degree of functionalization to be quantified.[24] In all cases, the calculated degrees of functionalization are lower than those of the corresponding Ugi-A materials, yet consistent with partial incorporation of the functionalized linker relative to the idealized feed ratio. Overall, elemental analysis confirms the successful introduction of Ugi-derived

functionalities into the UiO-67 framework and demonstrates that the degree of functionalization can be effectively modulated through either full linker substitution (Ugi-A) or a mixed-linker MTV strategy (Ugi-B).

5.8 MOFs digestion and ^{1}H NMR analysis

A 1 M sodium bicarbonate ($NaHCO_3$) solution was prepared using deuterated water (D_2O). Subsequently, 20 mg of each MOF sample were dispersed in 1 mL of this solution. The resulting suspensions were sonicated for 20 min to ensure complete digestion of the framework. The obtained solutions were then directly analyzed by ^{1}H NMR spectroscopy.[25] The ^{1}H NMR spectra display characteristic signals corresponding to both the parent biphenyl dicarboxylate (BPDC) linker and the Ugi-functionalized linker. The presence of the Ugi moiety was confirmed by the appearance of a singlet at δ ≈ 1.0–1.3 ppm, assigned to the tert-butyl group (9H), while the aromatic region (δ ≈ 7.5–8.0 ppm) corresponds to the biphenyl protons present in both linkers. The residual solvent signal (HOD) was observed at δ ≈ 4.7 ppm.

The relative incorporation of the Ugi-functionalized linker in the MTV-MOF samples was estimated by comparing the integrals of the tert-butyl signal (9H, exclusive to the Ugi linker) and the aromatic region associated with the biphenyl moieties.[26] The fraction of Ugi linker was calculated according to the following expression:

$$\textbf{Ugi fraction} = \frac{(I_{tBu} / 9)}{(I_{Ar} / 8)}$$

where I_{tBu} corresponds to the integral of the tert-butyl signal and I_{Ar} to the integral of the aromatic region. The aromatic signals were integrated over the range δ ≈ 7.5–8.0 ppm, ensuring that only well-defined resonances were considered to minimize baseline contributions. This analysis provided a semiquantitative estimation of the linker composition in the MTV-MOF materials.

Table S8. Quantification of linker composition in MTV UiO-67 materials determined by ^{1}H NMR spectroscopy after digestion.

Material	Ugi-derived linker (%)	H_2BPDC (%)
**MOF Ugi-1B**	19.9	80.1
**MOF Ugi-2B**	26.9	73.1
**MOF Ugi-3B**	11.1	88.9
**MOF Ugi-4B**	43.2	56.8

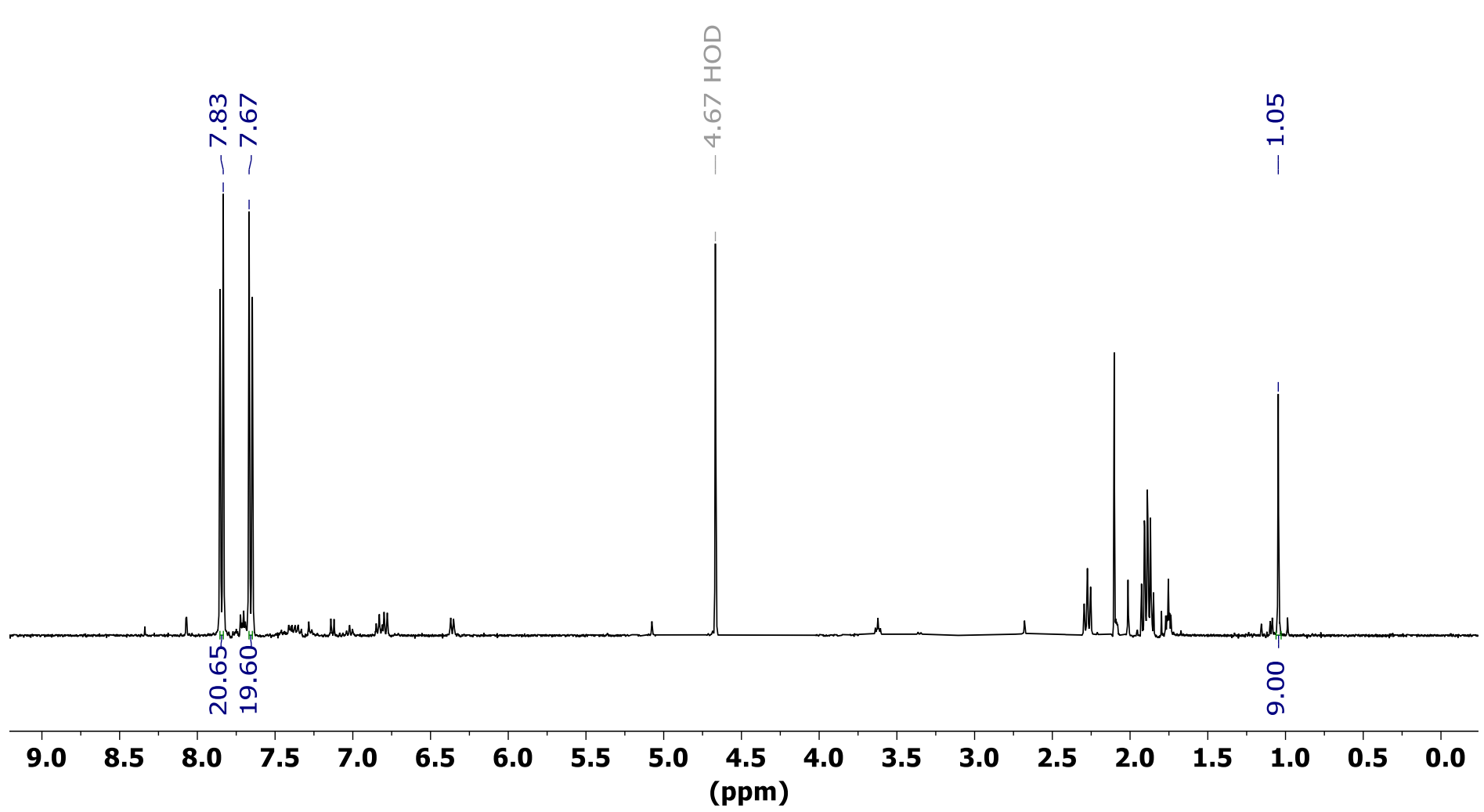

Figure S39. ^{1}H NMR spectra of digested MOF **Ugi-1B** (D_2O, 400 MHz).

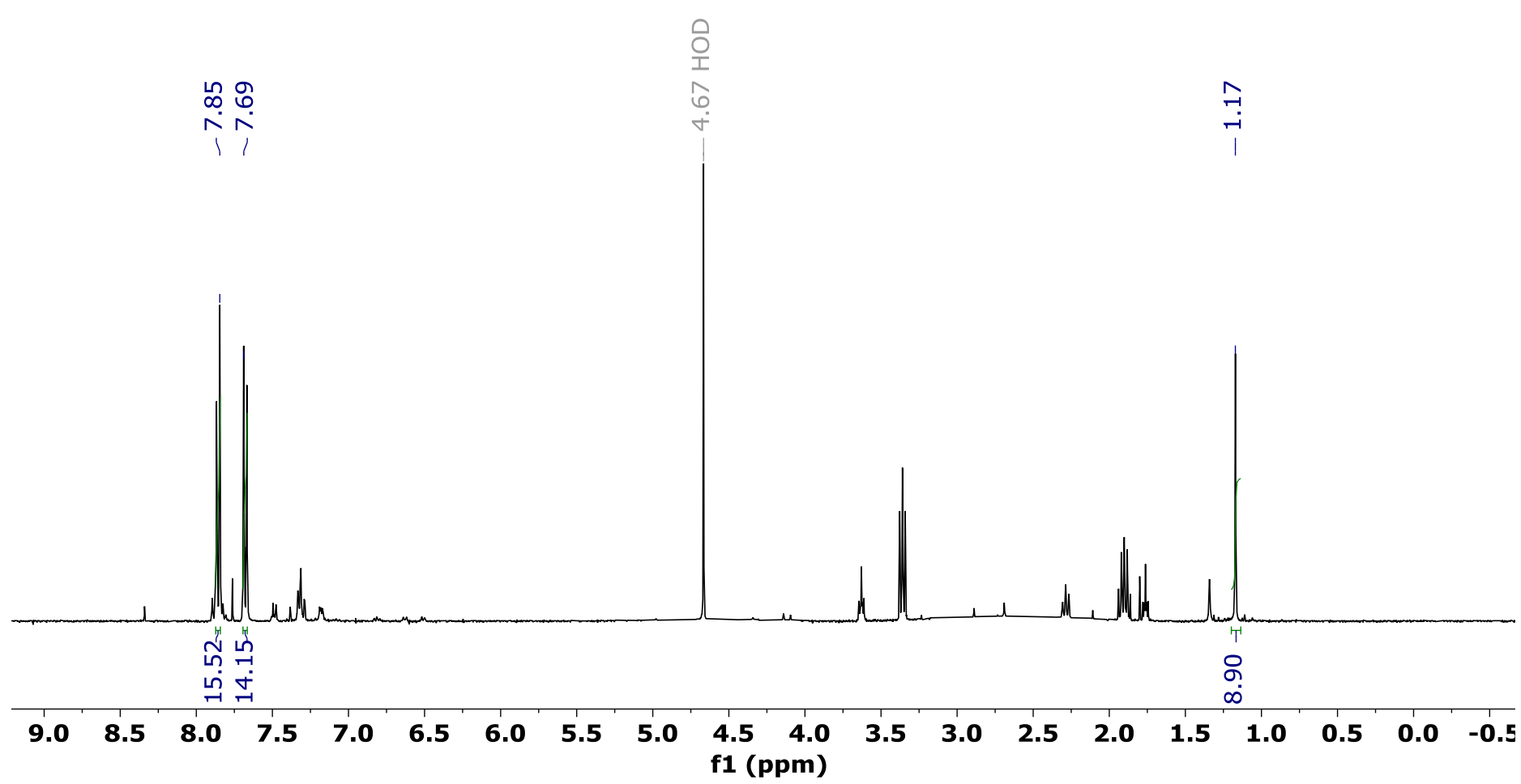

Figure S40. ^{1}H NMR spectra of digested MOF **Ugi-2B** (D_2O, 400 MHz).

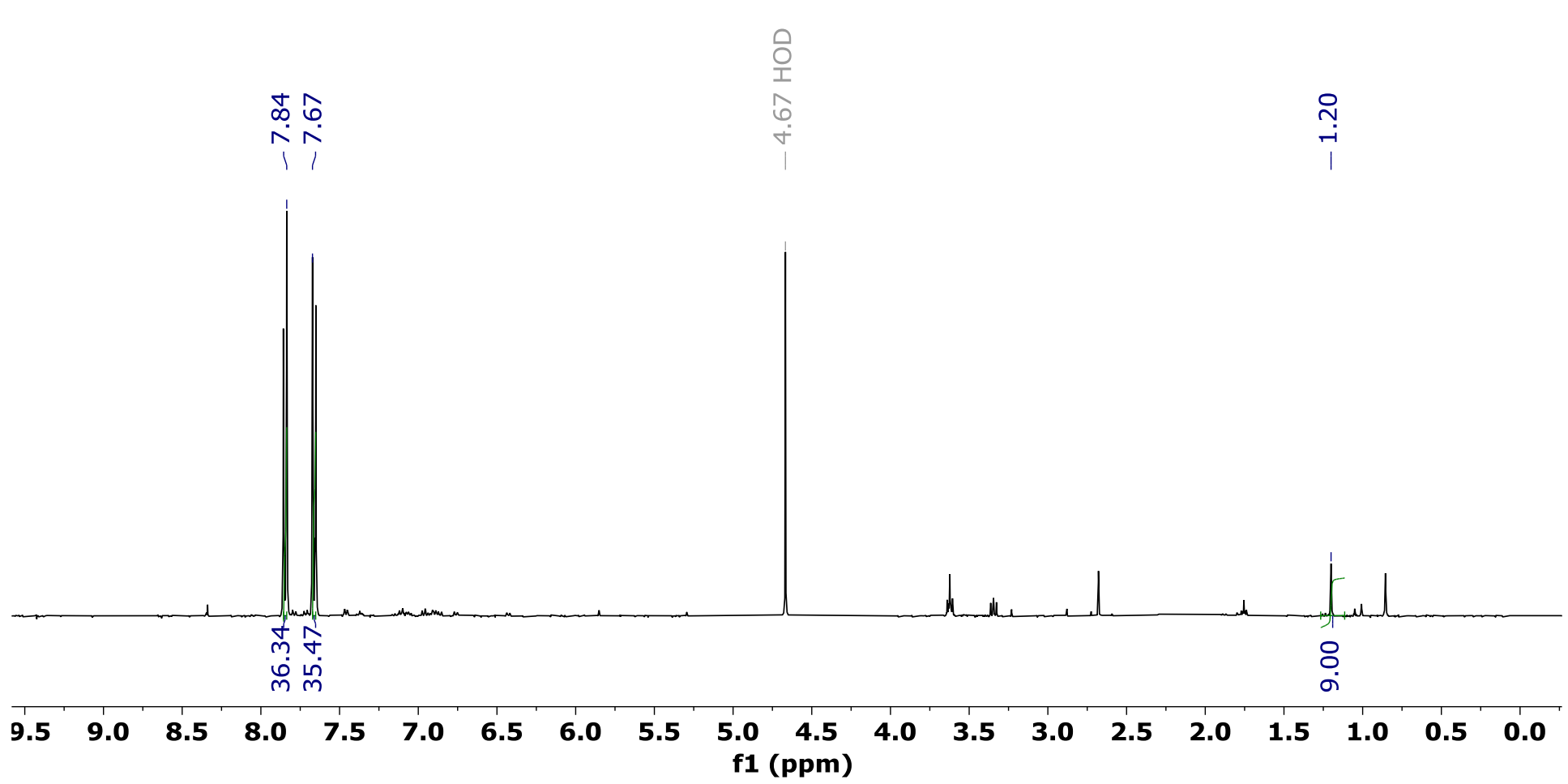

Figure S41. ^{1}H NMR spectra of digested MOF **Ugi-3B** (D_2O, 400 MHz).

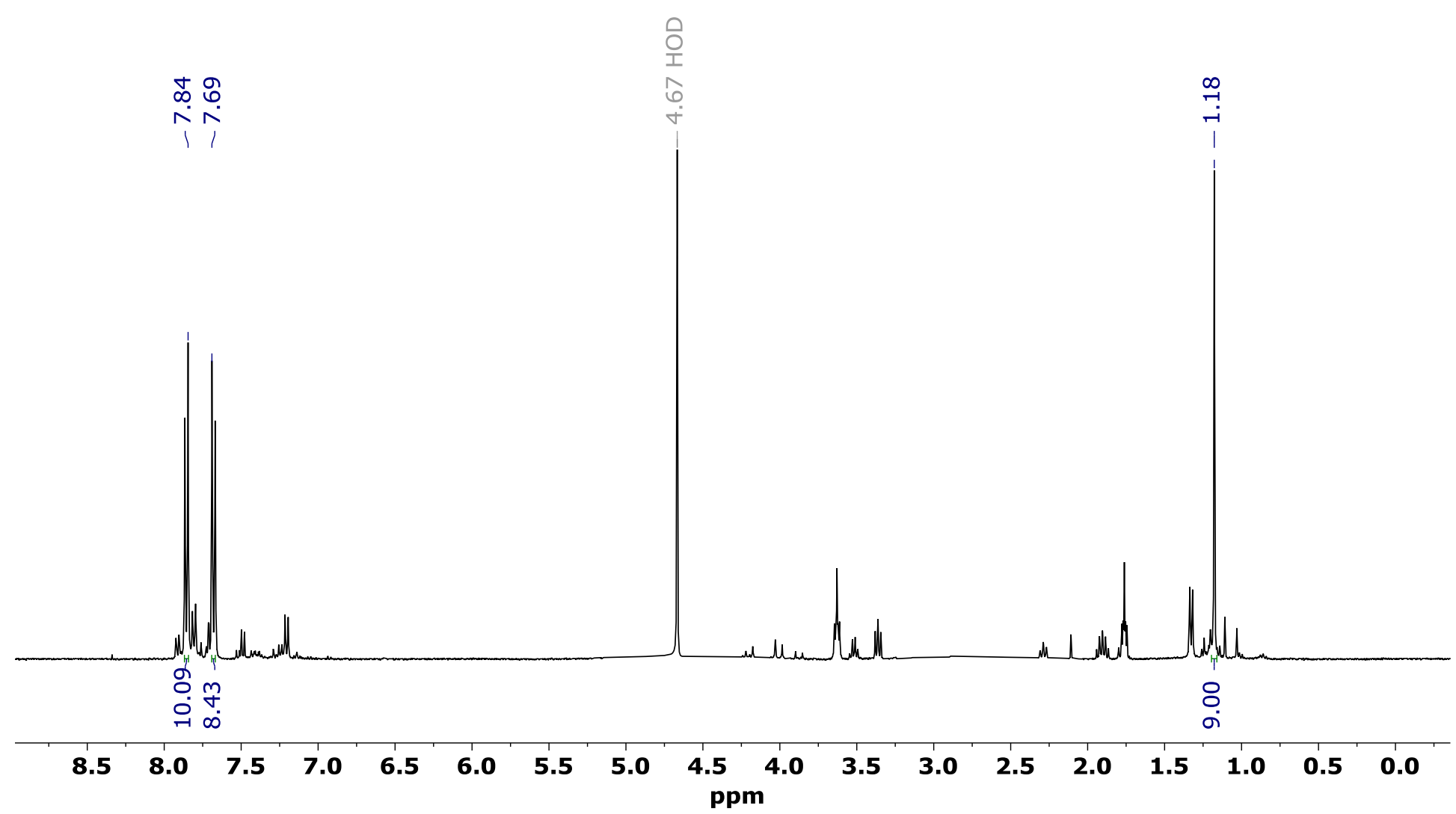

Figure S42. ^{1}H NMR spectra of digested **MOF Ugi-4B** (D_2O, 400 MHz).

The linker composition in the Ugi MTV-MOF materials was quantified by integration of the characteristic signals in the ^{1}H NMR spectra of the digested samples (**Table S8**). As shown, the incorporation of the Ugi-derived linker deviates from the initial synthetic ratios, yielding 19.9%, 26.9%, 11.1% and 43.2% for MOF Ugi-1B, MOF Ugi-2B, MOF Ugi-3B and MOF Ugi-4B, respectively. This behavior suggests a non-statistical incorporation of the functionalized linker, likely governed by differences in solubility, steric demand, and coordination kinetics during MOF crystallization. In particular, the bulkier and more functionalized Ugi-derived linker may experience diffusion or coordination limitations compared to H_2BPDC, leading to preferential incorporation of the latter in certain cases. Notably, MOF Ugi-4B exhibits the highest incorporation of the Ugi-derived linker, whereas MOF Ugi-3B shows a significantly lower content, indicating limited insertion under those conditions. These variations point to a delicate balance between linker reactivity and framework assembly dynamics, which ultimately dictates the final composition of the MTV materials.

The corresponding ^{1}H NMR spectra (**Figure S39-Figure S42**) further corroborate these findings, displaying well-resolved and distinguishable signals attributable to both the Ugi-functionalized linker and H_2BPDC, thereby enabling accurate integration. Importantly, these results are in good agreement with those obtained from elemental analysis (**Table S7**), supporting the reliability of the quantification and the proposed compositions. Overall, these results confirm the successful formation of mixed-linker frameworks while highlighting the inherent challenges in achieving precise compositional control in MTV systems, which is expected to have a direct impact on the physicochemical properties and adsorption behavior of the resulting materials.

6. Catechol Adsorption and HPLC Quantification

6.1 Calibration Curve of catechol

A calibration curve was constructed using standard solutions of catechol at eight different concentrations (0.125–2.5 mM), each analyzed in triplicate by HPLC under the conditions described above. Standard solutions were prepared by dilution of a stock solution of catechol in acetonitrile.

For each concentration level (0.125, 0.25, 0.375, 0.625, 0.875, 1.25, 1.875, and 2.5 mM), three independent injections were performed, and the corresponding peak areas at 275 nm were recorded. The average peak area for each concentration was used to construct the calibration curve.

A linear relationship between catechol concentration and peak area was observed over the studied range. The calibration curve was obtained by linear regression analysis of the averaged data, following the equation:

$$y = mx + b$$

where y is the peak area and x is the catechol concentration (mM). The resulting calibration curve showed excellent linearity, with a correlation coefficient (R^2) of 0.9997.

6.2 Quantification Methodology

The concentration of catechol in solution after adsorption experiments was determined by HPLC using the calibration curve described above. The peak area at 275 nm was converted to concentration (mM) using the linear regression equation obtained from the calibration curve.

The final concentration of catechol (C_f) was calculated from the measured peak area using the equation:

$$C_f = \frac{y-b}{m}$$

where y is the peak area, and m and b are the slope and intercept of the calibration curve, respectively. Adsorption experiments were conducted using an initial catechol concentration (C_0) of 2.5 mM in acetonitrile. After equilibration, the supernatant was analyzed by HPLC to determine C_f.

The amount of catechol adsorbed by the MOF was calculated using the following equation[27]:

$$q = \frac{(C_0 - C_f) \cdot V}{m}$$

where q is the adsorption capacity ($mg \cdot g^{-1}$), C_0 and C_f are the initial and final concentrations ($mg \cdot L^{-1}$), V is the volume of the solution (10 mL), and m is the mass of MOF used (20 mg). Concentrations obtained in mM were converted to $mg \cdot L^{-1}$ (ppm) prior to the calculation of adsorption capacity.

6.3 Adsorption Experiments and HPLC Analysis

Adsorption experiments were performed using previously activated MOFs. **UiO-67, UiO-67-Ugi-1A, and UiO-67-Ugi-1B** were thermally activated at 100 °C for 12 h prior to use.

In a typical experiment, 20 mg of each MOF were dispersed in 10 mL of a 2.5 mM catechol solution in acetonitrile. The suspensions were stirred at room temperature for 24 h to ensure adsorption equilibrium.

After equilibration, the resulting solids were separated by filtration using 0.2 µm syringe filters (Acrodisc), washed with acetonitrile to remove weakly adsorbed or residual catechol species, and subsequently collected for further characterization. The supernatant solutions were directly analyzed by HPLC under the conditions described in Section 1.2.

The concentration of catechol remaining in solution was determined from the corresponding chromatographic peak area using the calibration curve described above. All adsorption experiments and HPLC measurements were performed in triplicate to ensure reproducibility.

Table S9. HPLC Quantification and Adsorption Results of catechol.

Sample	Mean Peak Area	Std Dev (s)	Relative Error (%)	C_f (ppm)	Adsorption ($mg \cdot g^{-1}$)
**UiO-67**	2765.79	50.52	1.83	214.7	30.3
**MOF Ugi-1A**	3054.08	23.48	0.77	236.7	19.3
**MOF Ugi-1B**	2581.59	6.34	0.24	199.9	37.7
**UiO-67-NH$_2$**	3279.37	4.10	0.12	254.3	10.5
**MOF Ugi-4 B**	3188.78	21.16	0.66	246.6	14.3

Final concentrations (C_f) were calculated from the calibration curve (Section 6.1), and adsorption capacities were determined as described in Section 6.2.

7. Post-Adsorption Characterization

7.1 FTIR spectra of Catechol-Loaded MOFs

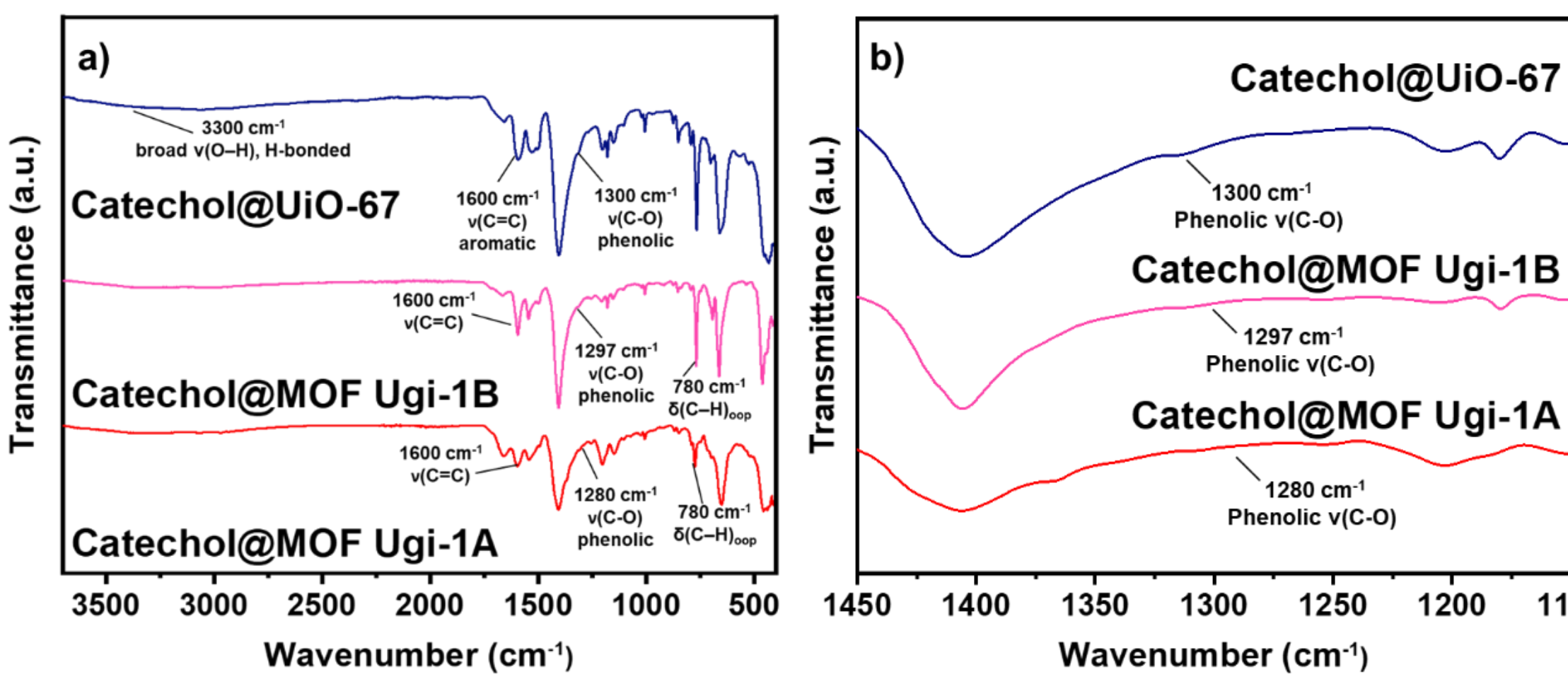

Figure S43. FT-IR analysis of catechol-loaded UiO-67-based materials. **a)** Full FT-IR spectra of Catechol@UiO-67, Catechol@MOF Ugi-1B, and Catechol@MOF Ugi-1A showing characteristic vibrational features associated with adsorbed catechol species. **b)** Expanded view of the 1450–1150 cm^{-1} region highlighting the phenolic ν(C–O) stretching vibrations associated with catechol incorporation within the materials.

The incorporation of catechol within the framework was investigated by FT-IR spectroscopy (**Figure S43 a)**). All materials exhibit characteristic vibrational features consistent with the presence of aromatic species and phenolic functionalities after adsorption. For **Catechol@UiO-67**, a broad band centered at ~3300 cm^{-1} is observed, which is assigned to hydrogen-bonded ν(O–H) stretching vibrations. This feature is consistent with the presence of phenolic hydroxyl groups from catechol within the porous framework. Additionally, a band at ~1600 cm^{-1} corresponds to ν(C=C) stretching of the aromatic ring, while the appearance of a distinct signal at ~1300 cm^{-1}, more clearly observed in the expanded region shown in (**Figure S43 b)**), is attributed to ν(C–O) stretching vibrations of phenolic groups, providing direct evidence of catechol incorporation.28 A further contribution at ~780 cm^{-1}, assigned to out-of-plane δ(C–H) bending vibrations of a 1,2-disubstituted aromatic ring, is consistent with the ortho-substituted structure of catechol.[28]

Similar spectral features are observed for the functionalized materials. In **Catechol@MOF Ugi-1B**, the band at ~1297 cm^{-1}, highlighted in **Figure S43 b)**, confirms the presence of phenolic C–O groups, while the signal at ~1600 cm^{-1} is associated with aromatic ν(C=C) stretching modes. The band at ~780 cm^{-1} further supports the presence of a 1,2-disubstituted aromatic system. Compared to pristine **UiO-67**, these features indicate successful incorporation of catechol within the modified pore environment.

For **Catechol@MOF Ugi-1A**, analogous bands are observed at ~1280 cm^{-1} (ν(C-O), phenolic), ~1600 cm^{-1} (ν(C=C), aromatic), and ~780 cm^{-1} (δ(C–H)oop), confirming the presence of adsorbed catechol in the material. Notably, slight variations in band intensity and definition among the three materials suggest differences in adsorption environments, likely arising from the distinct chemical functionalities introduced via Ugi modification. Overall, the consistent appearance of phenolic C-O stretching bands in the 1280-1300 cm^{-1} region, highlighted in the expanded spectra shown in Figure S40b, together with the broad O-H signal and characteristic aromatic vibrations, provides strong spectroscopic evidence for catechol incorporation in both pristine and functionalized UiO-67-derived materials. The observed spectral features further suggest that functionalization modulates the local adsorption environment and may influence host–guest interactions within the material.[29]

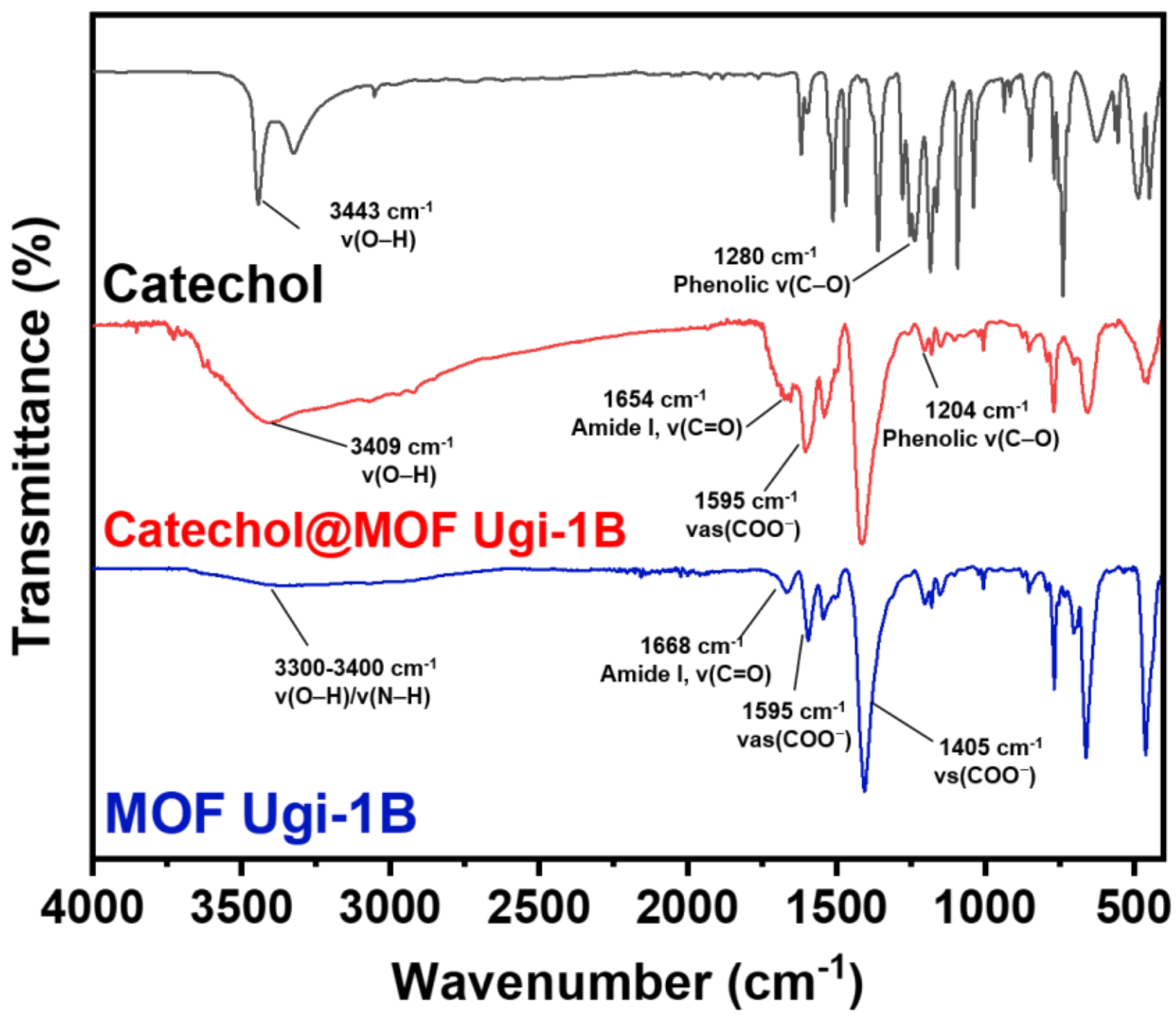

Figure S44. FTIR spectra (KBr) of catechol (black), MOF Ugi-1B (blue), and catechol-loaded MOF Ugi-1B after washing (red). The amide ν(C=O) shifts from ~1668 to ~1654 cm^{-1} upon loading, while the coordinated carboxylate band (~1595 cm^{-1}) remains unchanged as an internal reference.

To probe the amide site within the framework, FTIR spectra (KBr) of MOF Ugi-1B were recorded before and after catechol adsorption, using the loaded material recovered after thorough washing (Figure S44). Upon loading, the amide carbonyl shifts by ~14 cm^{-1} to a lower wavenumber (~1668 → ~1654 cm^{-1}), as expected when the C=O acts as a hydrogen-bond acceptor, while the Zr-coordinated carboxylate band (~1595 cm^{-1}) remains unchanged as an internal reference. A concurrent red-shift of the ν(O-H) region (~3443 → ~3409 cm^{-1}) provides corroborating evidence, though it is interpreted with caution owing to moisture absorption from the KBr matrix. Catechol remained detectable after washing, indicating retention within the framework.

7.2 TGA Analysis of Catechol-Loaded MOFs

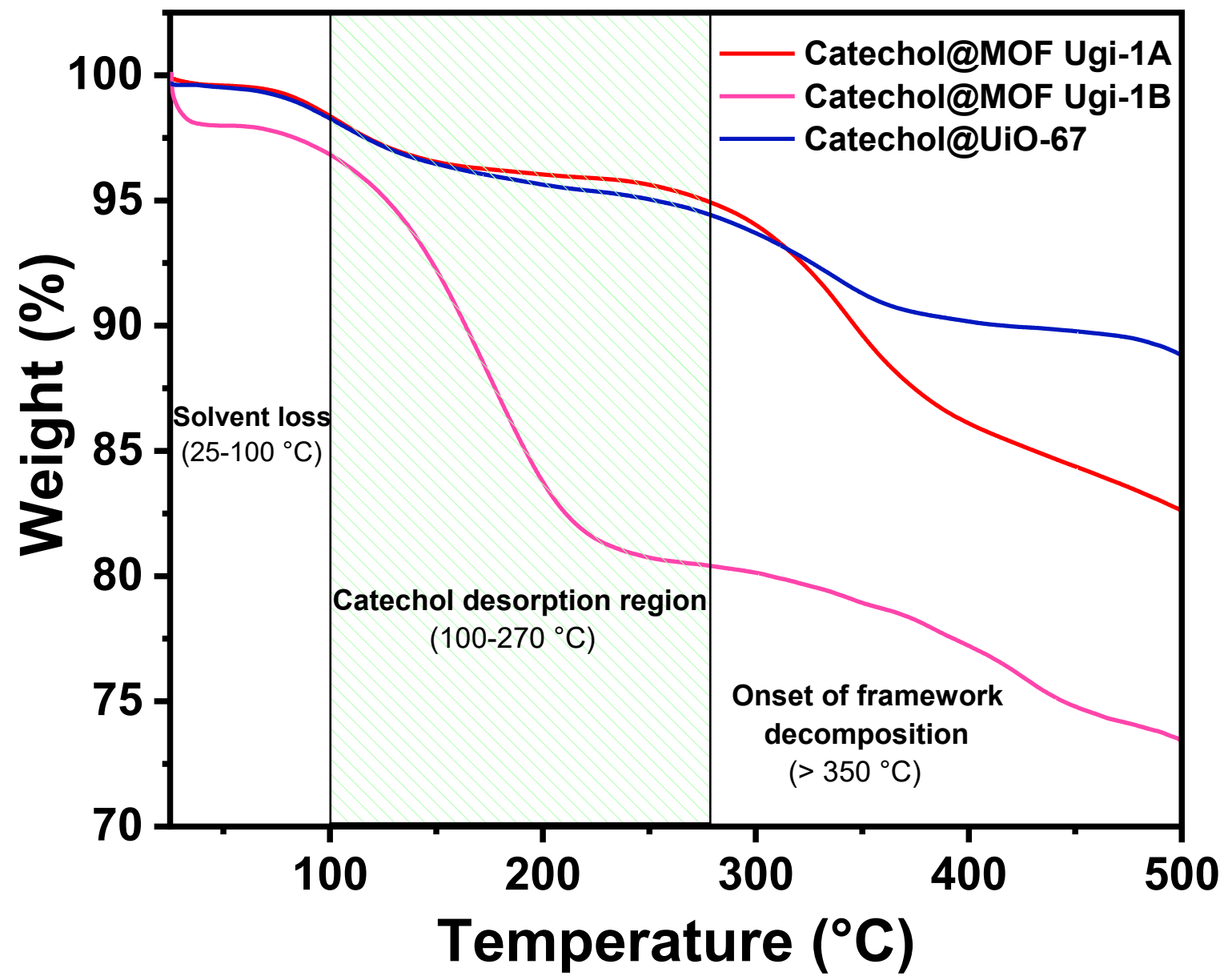

Figure S45. Thermogravimetric analysis (TGA) profiles of **Catechol@UiO-67** and **Ugi-functionalized derivatives** after catechol adsorption, highlighting solvent loss, catechol desorption, and onset of framework decomposition.

Thermogravimetric analysis (TGA) was conducted to evaluate the thermal behavior of catechol-loaded materials and to provide complementary evidence for guest incorporation within the porous frameworks (**Figure S45**). All samples exhibited multistep weight-loss profiles associated with residual solvent removal, catechol desorption, and framework decomposition at elevated temperatures. An initial low-weight-loss event observed between 25 and 100 °C was attributed to the release of weakly physisorbed acetonitrile molecules remaining within the porous framework or adsorbed at the external surface after the adsorption experiments. The relatively small mass variation in this region suggests efficient activation and solvent removal prior to thermal analysis.

A second and substantially more pronounced weight-loss process was observed between 100 and 270 °C, corresponding to the thermal desorption of adsorbed catechol molecules. The extent of mass loss within this temperature range strongly depended on the framework functionality, revealing significant differences in guest retention among the investigated materials. **Catechol@MOF Ugi-1B** exhibited the largest weight loss in this region, indicating enhanced catechol uptake and stronger host–guest interactions relative to the other materials. In contrast, pristine **Catechol@UiO-67** displayed only a comparatively small mass decrease, consistent with weaker interactions and lower catechol retention capacity. **Catechol@MOF Ugi-1A** showed intermediate behavior.

The enhanced catechol retention observed for the Ugi-functionalized derivatives is attributed to the presence of additional polar functionalities introduced through the multicomponent pre-synthetic modification strategy. These functional groups likely promote stronger intermolecular interactions with catechol molecules through hydrogen-bonding and dipolar interactions, thereby increasing guest affinity within the porous structure.[30,31] These observations are consistent with the adsorption studies and further support the influence of framework functionalization on catechol uptake behavior.

Importantly, the primary catechol desorption process occurred prior to the main framework decomposition event, indicating that guest release takes place before significant thermal degradation of the UiO-67-derived materials. The onset of decomposition was observed above approximately 350 °C for all materials, consistent

with the characteristic thermal stability commonly reported for zirconium-based UiO-type MOFs.[13,16,24] Although slight differences in the high-temperature decomposition profiles were observed, all materials maintained substantial thermal resistance following catechol incorporation.

Overall, the TGA results provide additional evidence for successful catechol adsorption and demonstrate that Ugi-derived functionalization significantly modulates guest retention behavior within the UiO-67-derived materials. The larger mass loss observed for **Catechol@MOF Ugi-1B** is consistent with the enhanced adsorption capacity determined by HPLC analysis.

7.3 Morphological Characterization of UiO-67-Based Materials after Catechol Adsorption

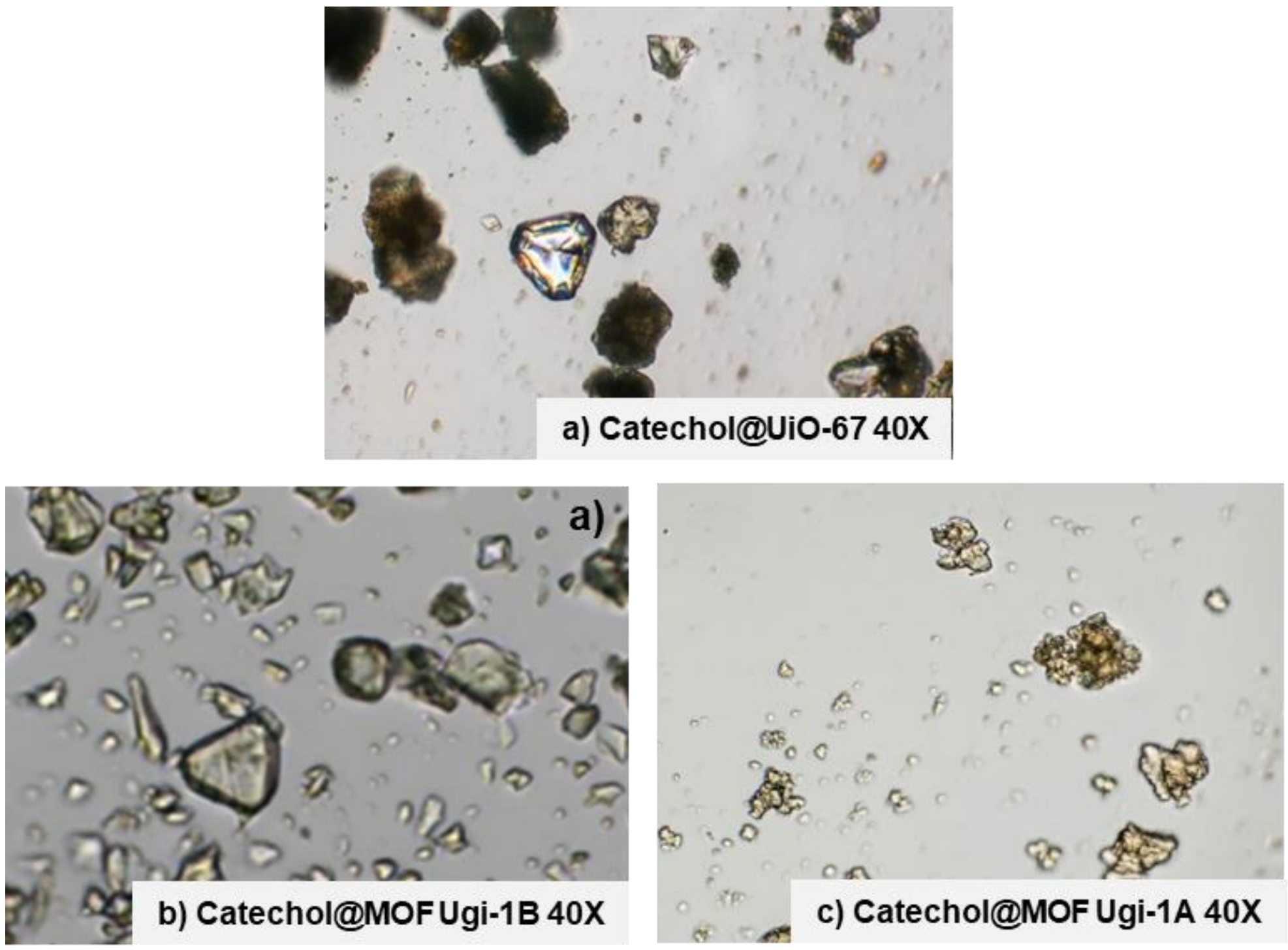

Figure S46. Optical microscopy images of catechol-loaded MOFs: a) **Catechol@UiO-67**, b) **Catechol@MOF Ugi-1B**, and c) **Catechol@MOF Ugi-1A**.

The optical microscopy images obtained after catechol adsorption reveal important differences in the morphological behavior of the evaluated materials. For **Catechol@UiO-67** (**Figure S46 a)**), well-defined crystalline particles with characteristic faceted morphology are still observed after adsorption, indicating that the parent UiO-67 framework preserves its structural integrity under the experimental conditions. Although slight particle aggregation is detected, the presence of distinguishable crystalline domains suggests that catechol incorporation does not significantly affect the morphology of the material.

Similarly, **Catechol@MOF Ugi-1B** (**Figure S46 b)**) exhibits recognizable crystalline particulates after adsorption. The material shows a broader particle size distribution and the presence of fragmented microcrystals; however, discrete crystalline domains remain clearly visible. These observations are consistent with the retention of crystallinity after catechol uptake, in agreement with the PXRD results obtained for this material.

In contrast, the optical micrographs of **Catechol@MOF Ugi-1A** (**Figure S46 c)**) display predominantly irregular agglomerated domains lacking the well-defined faceted morphology observed for **UiO-67** and **MOF Ugi-1B**. The particles appear highly clustered and poorly defined, suggesting a substantial loss of long-range structural order after adsorption. These observations are consistent with the PXRD analysis, where **MOF Ugi-**

1A exhibited an essentially amorphous pattern following catechol exposure. The altered morphology may indicate that the framework is less stable under the adsorption conditions, possibly due to the nature of the incorporated Ugi-derived functionality.

Overall, the optical microscopy analysis supports the higher structural stability of **UiO-67** and **MOF Ugi-1B** during catechol adsorption, whereas **MOF Ugi-1A** undergoes significant morphological and crystallographic deterioration under the same conditions.

8. References

(1) Brunauer, S.; Emmett, P. H.; Teller, E. Adsorption of Gases in Multimolecular Layers. J. Am. Chem. Soc. 1938, 60, 309–319. https://doi.org/10.1021/ja01269a023

(2) Pines, A.; Gibby, M. G.; Waugh, J. S. Proton-Enhanced Nuclear Induction Spectroscopy. A Method for High Resolution NMR of Dilute Spins in Solids. J. Chem. Phys. 1973, 59, 569–590. https://doi.org/10.1063/1.1680061

(3) Hartmann, S. R.; Hahn, E. L. Nuclear Double Resonance in the Rotating Frame. Phys. Rev. 1962, 128, 2042–2053. https://doi.org/10.1103/PhysRev.128.2042

(4) Fung, B. M.; Khitrin, A. K.; Ermolaev, K. An Improved Broadband Decoupling Sequence for Liquid Crystals and Solids. J. Magn. Reson. 2000, 142, 97–101. https://doi.org/10.1006/jmre.1999.1896

(5) Vogel, A. I. Vogel's Textbook of Practical Organic Chemistry, 5th ed.; Longman Scientific & Technical: Harlow, U.K., 1989.

(6) Dömling, A. Recent Developments in Isocyanide Based Multicomponent Reactions in Applied Chemistry. Chem. Rev. 2006, 106, 17–89. https://doi.org/10.1021/cr0505728

(7) Dömling, A.; Wang, W.; Wang, K. Chemistry and Biology of Multicomponent Reactions. Chem. Rev. 2012, 112, 3083–3135. https://doi.org/10.1021/cr100233r

(8) Nógrádi, M.; Tóth, A. Stereoselective Multicomponent Reactions and Their Synthetic Applications. Chem. Soc. Rev. 2014, 43, 6229–6246. https://doi.org/10.1039/C4CS00015F

(9) Quintanilla-Licea, R.; Colunga-Valladares, J. F.; Caballero-Quintero, A.; Rodríguez-Padilla, C.; Tamez-Guerra, R.; Gómez-Flores, R.; Waksman, N. NMR Detection of Isomers Arising from Restricted Rotation of the C–N Amide Bond of N-Formyl-o-toluidine and N,N′-bis-Formyl-o-tolidine. Molecules 2002, 7, 662–673. https://doi.org/10.3390/70900662

(10) Thommes, M.; Kaneko, K.; Neimark, A. V.; Olivier, J. P.; Rodriguez-Reinoso, F.; Rouquerol, J.; Sing, K. S. W. Physisorption of Gases, with Special Reference to the Evaluation of Surface Area and Pore Size Distribution. Pure Appl. Chem. 2015, 87, 1051–1069. https://doi.org/10.1515/pac-2014-1117

(11) Furukawa, H.; Cordova, K. E.; O'Keeffe, M.; Yaghi, O. M. The Chemistry and Applications of Metal–Organic Frameworks. Science 2013, 341, 1230444. https://doi.org/10.1126/science.1230444

(12) Deria, P.; Mondloch, J. E.; Hupp, J. T.; Farha, O. K. Enhancing the Rate of Ligand Exchange in Zirconium-Based Metal–Organic Frameworks through Linker Defect Engineering. J. Am. Chem. Soc. 2013, 135, 16801–16804. https://doi.org/10.1021/ja408939c

(13) Cavka, J. H.; Jakobsen, S.; Olsbye, U.; Guillou, N.; Lamberti, C.; Bordiga, S.; Lillerud, K. P. A New Zirconium Inorganic Building Brick Forming Metal–Organic Frameworks with Exceptional Stability. J. Am. Chem. Soc. 2008, 130, 13850–13851. https://doi.org/10.1021/ja8057953

(14) Furukawa, H.; Müller, U.; Yaghi, O. M. Heterogeneity within Order in Metal–Organic Frameworks. Angew. Chem. Int. Ed. 2015, 54, 3417–3430. https://doi.org/10.1002/anie.201410252

(15) Cliffe, M. J.; Wan, W.; Zou, X.; Chater, P. A.; Kleppe, A. K.; Tucker, M. G.; Wilhelm, H.; Funnell, N. P.; Coudert, F.-X.; Goodwin, A. L. Correlated Defect Nanoregions in a Metal–Organic Framework. Nat. Commun. 2014, 5, 4176. https://doi.org/10.1038/ncomms5176

(16) Shearer, G. C.; Chavan, S.; Ethiraj, J.; Vitillo, J. G.; Svelle, S.; Olsbye, U.; Lamberti, C.; Bordiga, S.; Lillerud, K. P. Defect Engineering: Tuning the Porosity and Composition of the UiO-66 Framework. Chem. Mater. 2016, 28, 3749–3761. https://doi.org/10.1021/acs.chemmater.6b00602

(17) Katz, M. J.; Brown, Z. J.; Colón, Y. J.; Siu, P. W.; Scheidt, K. A.; Snurr, R. Q.; Hupp, J. T.; Farha, O. K. A Facile Synthesis of UiO-66, UiO-67 and Their Derivatives. Chem. Commun. 2013, 49, 9449–9451. https://doi.org/10.1039/C3CC46105J

(18) Li, J.-R.; Kuppler, R. J.; Zhou, H.-C. Selective Gas Adsorption and Separation in Metal–Organic Frameworks. Chem. Soc. Rev. 2009, 38, 1477–1504. https://doi.org/10.1039/B802426J

(19) Silverstein, R. M.; Webster, F. X.; Kiemle, D. J. Spectrometric Identification of Organic Compounds; Wiley: Hoboken, NJ, 2005. https://doi.org/10.1002/0471656040

(20) Kitagawa, S.; Kitaura, R.; Noro, S.-I. Functional Porous Coordination Polymers. Angew. Chem. Int. Ed. 2004, 43, 2334–2375. https://doi.org/10.1002/anie.200300610

(21) Valenzano, L.; Civalleri, B.; Chavan, S.; Bordiga, S.; Nilsen, M. H.; Jakobsen, S.; Lillerud, K. P.; Lamberti, C. Disclosing the Complex Structure of UiO-66 Metal Organic Framework: A Synergic Combination of Experiment and Theory. Chem. Mater. 2011, 23, 1700–1718. https://doi.org/10.1021/cm1022882

(22) Bueken, B.; Van Velthoven, N.; Willhammar, T.; Stassin, T.; Stassen, I.; Keen, D. A.; Baron, G. V.; Denayer, J. F. M.; Ameloot, R.; Bals, S.; De Vos, D. Post-Synthetic Defect Exchange in the Zr-Based Metal–Organic Framework UiO-66. Chem. Sci. 2017, 8, 3939–3948. https://doi.org/10.1039/C7SC00811B

(23) Furukawa, H.; Gandara, F.; Zhang, Y.-B.; Jiang, J.; Queen, W. L.; Hudson, M. R.; Yaghi, O. M. Water Adsorption in Porous Metal–Organic Frameworks and Related Materials. J. Am. Chem. Soc. 2014, 136, 4369–4381. https://doi.org/10.1021/ja500330a

(24) Yuan, S.; Qin, J.-S.; Lollar, C. T.; Zhou, H.-C. Stable Metal–Organic Frameworks with Group 4 Metals: Current Status and Trends. ACS Cent. Sci. 2018, 4, 440–450. https://doi.org/10.1021/acscentsci.8b00044

(25) Katz, M. J.; Mondloch, J. E.; Totten, R. K.; Park, J. K.; Nguyen, S. T.; Farha, O. K.; Hupp, J. T. Simple and Compelling Biomimetic Metal–Organic Framework Catalyst for the Degradation of Nerve Agent Simulants. Angew. Chem. Int. Ed. 2014, 53, 497–501. https://doi.org/10.1002/anie.201309307

(26) Garibay, S. J.; Cohen, S. M. Isoreticular Synthesis and Modification of Frameworks with the UiO-66 Topology. Chem. Commun. 2010, 46, 7700–7702. https://doi.org/10.1039/C0CC02990D

(27) Foo, K. Y.; Hameed, B. H. Insights into the Modeling of Adsorption Isotherm Systems. Chem. Eng. J. 2010, 156, 2–10. https://doi.org/10.1016/j.cej.2009.09.013

(28) Socrates, G. Infrared and Raman Characteristic Group Frequencies: Tables and Charts, 3rd ed.; Wiley: Chichester, U.K., 2001.

(29) Li, B.; Wen, H.-M.; Zhou, W.; Chen, B. Porous Metal–Organic Frameworks for Gas Storage and Separation: Status and Challenges. J. Phys. Chem. Lett. 2014, 5, 3468–3479. https://doi.org/10.1021/jz5015869

(30) Kaskel, S. The Chemistry of Metal–Organic Frameworks: Synthesis, Characterization, and Applications; Wiley-VCH: Weinheim, Germany, 2016. https://doi.org/10.1002/9783527693076

(31) Li, B.; Wen, H.-M.; Zhou, W.; Chen, B. Porous Metal–Organic Frameworks for Gas Storage and Separation: Status and Challenges. J. Phys. Chem. Lett. 2014, 5, 3468–3479. https://doi.org/10.1021/jz5015869
